# Four-Dimensional Lipidomic Analysis Using Comprehensive Online UHPLC × UHPSFC/Tandem Mass Spectrometry

**DOI:** 10.1021/acs.analchem.4c03946

**Published:** 2024-11-27

**Authors:** Zuzana Lásko, Tomáš Hájek, Robert Jirásko, Ondřej Peterka, Petr Šimek, Peter J. Schoenmakers, Michal Holčapek

**Affiliations:** † Department of Analytical Chemistry, University of Pardubice, Faculty of Chemical Technology, Studentská 573, Pardubice 53210, Czech Republic; ‡ Biology Centre of the Czech Academy of Sciences, České Budějovice 370 05, Czech Republic; § van ’t Hoff Institute for Molecular Sciences, Analytical Chemistry Group, University of Amsterdam, Science Park, 904, Amsterdam 1098 XH, The Netherlands

## Abstract

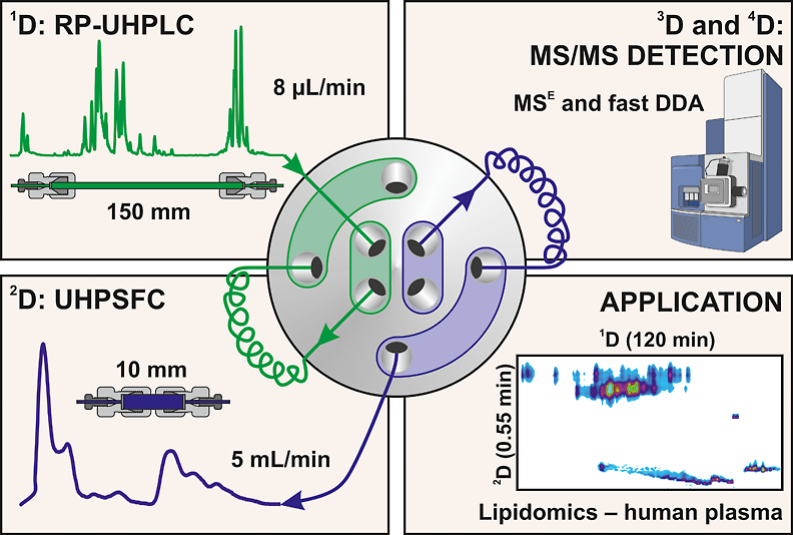

Multidimensional chromatography offers enhanced chromatographic resolution and peak capacity, which are crucial for analyzing complex samples. This study presents a novel comprehensive online multidimensional chromatography method for the lipidomic analysis of biological samples, combining lipid class and lipid species separation approaches. The method combines optimized reversed-phase ultrahigh-performance liquid chromatography (RP-UHPLC) in the first dimension, utilizing a 150 mm long C18 column, with ultrahigh-performance supercritical fluid chromatography (UHPSFC) in the second dimension, using a 10 mm long silica column, both with sub-2 μm particles. A key advantage of employing UHPSFC in the second dimension is its ability to perform ultrafast analysis using gradient elution with a sampling time of 0.55 min. This approach offers a significant increase in the peak capacity. Compared to our routinely used 1D methods, the peak capacity of the 4D system is 10 times higher than RP-UHPLC and 18 times higher than UHPSFC. The entire chromatographic system is coupled with a high-resolution quadrupole-time-of-flight (QTOF) mass analyzer using electrospray ionization (ESI) in both full-scan and tandem mass spectrometry (MS/MS) and with positive- and negative-ion polarities, enabling the detailed characterization of the lipidome. The confident identification of lipid species is achieved through characteristic ions in both polarity modes, information from MS elevated energy (MS^E^) and fast data-dependent analysis scans, and mass accuracy below 5 ppm. This analytical method has been used to characterize the lipidomic profile of the total lipid extract from human plasma, which has led to the identification of 298 lipid species from 16 lipid subclasses.

## Introduction

Two-dimensional chromatography (2D) is a powerful analytical technique that combines two different chromatographic modes in two consecutive separation dimensions and separates analytes according to distinct chemical properties. The primary aims of 2D are to achieve higher peak capacity and resolution and exceptional selectivity by using orthogonal separation mechanisms in two dimensions. This enables a more thorough separation and analysis of complex biological samples compared to conventional one-dimensional chromatography.^1^ The implementation of 2D chromatography requires careful planning and optimization of both separation dimensions. This may involve the selection of appropriate chromatographic modes, columns, mobile phases, detection methods for both dimensions, and finally, the interface between the first and second dimensions. Each dimension can employ one of the various separation mechanisms. These include ultrahigh-performance liquid chromatography (UHPLC) in normal-phase (NP),^2^ reversed-phase (RP),^2−6^ silver-ion,^5^ or hydrophobic interaction liquid chromatography (HILIC)^6,7^ modes, or gas chromatography.^8−10^ Recently, there has been an increasing trend in the literature to replace conventional LC with ultrahigh-performance supercritical fluid chromatography (UHPSFC).^3,4,11^

Lipidomics covers a vast array of lipid classes and subclasses, including numerous isomers that vary in their structures, as well as in their biological and chemical properties.^12,13^ The comprehensive characterization of human lipidome can provide critical insights in the pathophysiology of various diseases, such as cardiovascular diseases,^14−16^ diabetes,^17,18^ neurodegenerative diseases,^18−20^ and cancer.^21,22^ Investigating lipids facilitates the identification of specific lipid biomarkers associated with particular diseases and enhances our understanding of their roles in pathogenesis and disease progression. Chromatographic separations with mass spectrometry (MS) detection represent the most widely used approach for lipidomic analysis, but the conventional one-dimensional chromatographic techniques have limited ability to separate and identify all diverse lipids. Multidimensional techniques offer the possibility of combining various separation mechanisms to improve the separation of these compounds.

The lipid class separation approach, represented by NP-UHPLC, HILIC, and UHPSFC, separates lipids according to the polarity of the headgroup interacting with the polar stationary phase, while the lipid species separation approach using RP-UHPLC or RP-UHPSFC separates lipids according to the hydrophobic part of the molecule, i.e., the length of fatty acyl chains and the number and position of double bonds, resulting in the possible resolution of isomers.^23,24^ The combination of these two most common approaches in lipidomic analysis leads to the highest degree of orthogonality of the 2D lipidomic analysis.^2,6^ The characteristic conditions for the second dimension (^2^D) involve ultrafast separation, high flow rates, and the use of very short columns with particles down to sub-2 μm for upholding chromatographic resolution. UHPSFC is becoming an increasingly used chromatographic technique as ^2^D separation in 2D chromatography, particularly following RP-UHPLC in the first dimension (^1^D),^25−28^ due to high diffusion coefficients, low viscosity, and zero surface tension. Supercritical carbon dioxide (scCO_2_) enables rapid analysis with a high flow rate without loss of chromatographic resolution and relatively low pressure drops. Furthermore, the combination of scCO_2_ with various polar solvents used as modifiers ensures the comprehensive analysis of both polar and hydrophobic analytes.^29,30^ However, the application of UHPSFC in ^1^D requires the implementation of measures, such as the use of trapping columns, solvent exchange techniques, or specialized interfaces designed to remove CO_2_. Furthermore, the combination of a 2D separation system with MS can provide additional structural information, and the fragmentation of individual lipid species may be considered as additional dimensions for the comprehensive lipidomic analysis.^31^

Previous studies on 2D analysis in lipidomics describe the coupling of NP-UHPLC × RP-UHPLC,^32^ HILIC × RP-UHPLC,^33−35^ RP-UHPLC × HILIC,^6,36,37^ HILIC × nonaqueous RP-HPLC,^2^ silver-ion HPLC × NARP-HPLC,^38^ nonaqueous RP-HPLC × silver-ion HPLC,^5,39^ and UHPSFC × RP-UPHPC.^3,4,40^ Offline multidimensional coupling offers a technically simpler solution, allows for full optimization of both dimensions, and can reduce some issues related to the mobile phase incompatibility, although at the expense of longer analysis times. In this approach, samples are analyzed in ^1^D, the effluent is usually collected by the fraction collector and then stored or immediately transferred to the ^2^D system.^2,26,41^ In the online configuration, samples are transferred to the second dimension during the ^1^D separation, eliminating the need for intermediate steps. However, the biggest limitation of online separation is the very short separation time in the second dimension. The second dimension must complete its separation rapidly enough to handle the continuous flow of analytes from the first dimension, which can limit the resolution and peak capacity. Solutions to this problem include the use of short columns and high flow rates. The use of isocratic elution is the simplest way for rapid separation of analytes as it eliminates the need for column equilibration or pressure adjustment that are required in the gradient elution. However, it may not effectively separate complex samples, leading to long analysis times and dilution of strongly retained compounds. Gradient elution covers a broader range of analytes, providing more uniform peak widths and higher peak capacities.^2,6,42^

The main goal of this study is the development of a new online comprehensive RP-UHPLC × UHPSFC/MS/MS method applicable for the characterization of a wide range of lipids in biological samples. To the best of our knowledge, this is the first connection of UHPLC and UHPSFC in this configuration for lipidomics analysis, using a very short ^2^D column (10 × 2.1 mm; 1.7 μm), short modulation time (0.55 min), and gradient elution in both dimensions. The individual lipid species are identified according to their retention behaviors in both dimensions, mass accuracy of molecular adducts, and characteristic fragment ions measured by data-independent analysis (DIA) using MS^E^ and the fast-DDA MS acquisition mode.

## Experimental Section

### Materials

LiChrosolv chloroform stabilized with 2-methyl-2-butene was purchased from Merck (Darmstadt, Germany). Acetonitrile, 2-propanol, methanol, water, ammonium formate, formic acid (all LC/MS grade), and ammonium carbonate (≥30.0% NH_3_ basis) were obtained from Honeywell (Riedel-de Haën, Hamburg, Germany) or Sigma-Aldrich (St. Louis, MO, USA). Carbon dioxide of 4.5 grade (99.995%) was purchased from Messer Group (Bad Soden, Germany). Deionized water was prepared by a Milli-Q Reference Water Purification System (Molsheim, France). Endogenous lipid standards containing oleoyl fatty acyls (18:1) and internal standards were purchased from Merck. Nu-Chek (Elysian, MN, USA) and Avanti Polar Lipids (Alabaster, AL, USA). The final concentrations of all lipid standards in the mixture are shown in Table S1.

### Plasma Samples

The pooled human plasma was prepared by mixing aliquots of 200 human plasma samples from healthy volunteers (age 44–66 years and body mass index of 18–39) and used for method optimization and lipid identification. Plasma samples of 100 male and 100 female volunteers were collected from the Transfusion Department, University Hospital Olomouc, Czech Republic. The study was approved by the institutional ethical committee, and all subjects signed informed consent. All plasma samples were stored at −80 °C.

### Sample Preparation

The modified Folch extraction method^29^ was carried out as follows: 25 μL of plasma was mixed with 2 mL of chloroform and 1 mL of methanol and ultrasonicated for 15 min at ambient temperature. Next, 600 μL of 250 mM ammonium carbonate buffer was added, followed by ultrasonication for 15 min and centrifugation for 3 min (886×*g*). The collected organic phase was evaporated under a gentle steam of nitrogen, and the residue was dissolved in 50 μL mixture of chloroform/methanol (1:1, v/v) and vortexed for 1 min.

### UHPLC × UHPSFC/MS/MS Conditions

**Figure 1:**
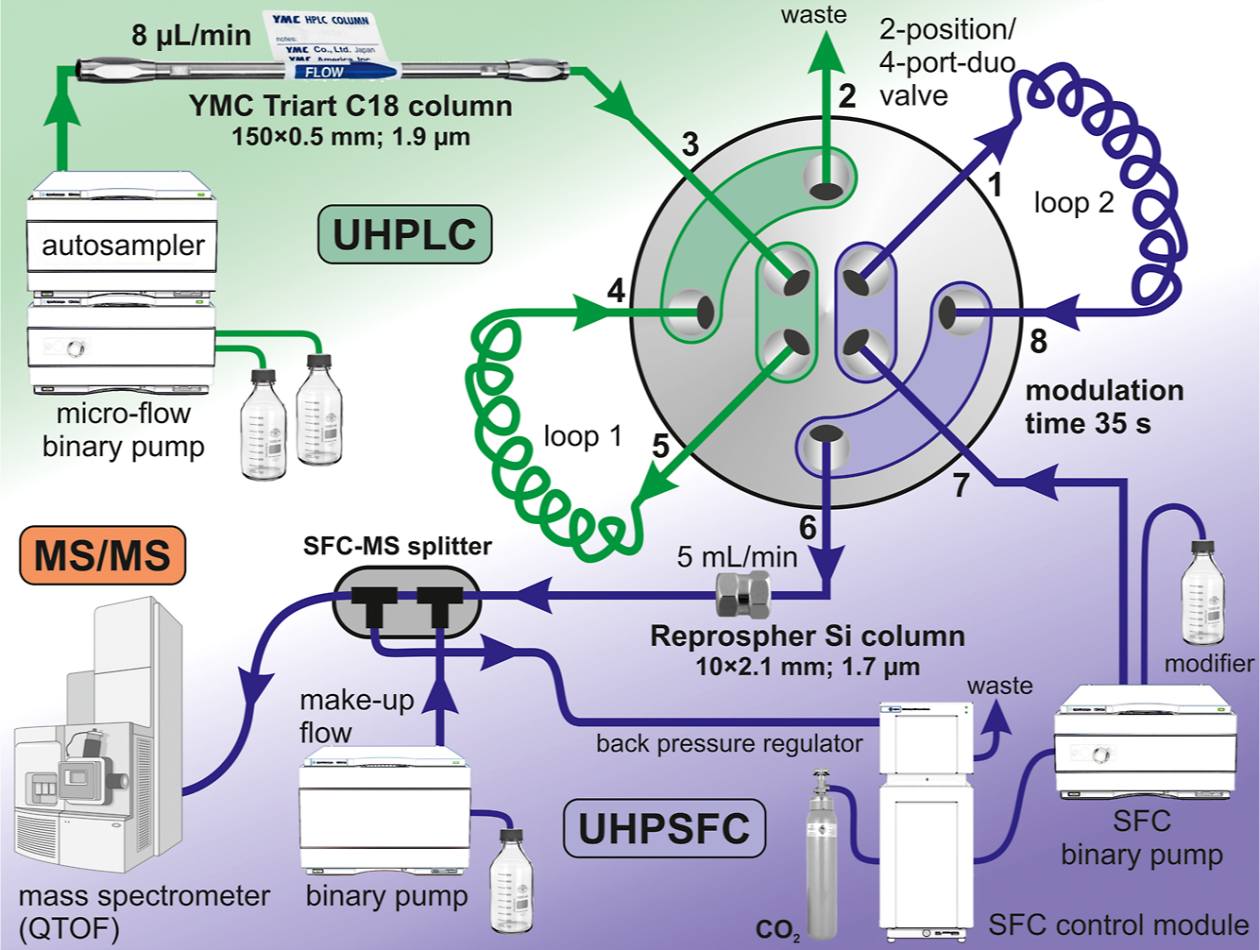
Schematic diagram of comprehensive online RP-UHPLC × UHPSFC/MS/MS system.

An Agilent 1260 Infinity capillary system, including a degasser, a microflow binary pump, and an autosampler in the first dimension, and an Agilent 1260 supercritical fluid chromatograph, containing an SFC binary pump, an SFC Control Module, an SFC-MS Splitter Kit, a binary pump for makeup flow in the second dimension, were used. Each dimension was individually controlled by ChemStation software (version B.04.03). The whole effluent from the first dimension microcolumn was transferred inline to the second dimension column in subsequent fractions collected alternately in two loops (5 μL) using a 2-position/4-port-duo valve interface between the first UHPLC and the second UHPSFC dimension columns (Figure 1). Both possible valve positions are shown in Figure S1A,B. All parts of the chromatographic configuration were produced by Agilent Technologies (Santa Clara, CA, USA). A YMC Triart C18 column (150 × 0.5 mm, 1.9 μm, YMC Co., Kyoto, Japan) was used in the first dimension under the following conditions: flow rate 8 μL/min, injection volume 0.5 μL, column temperature 55 °C, mobile phase gradient 0 min −35% MF(B)_D1_, 38 min −50% MF(B)_D1_, 100–110 min 95% MF(B)_D1_, and 115–120 min–35% MF(B)_D1_, where MF(A)_D1_ was 5 mmol/L ammonium formate and 0.1% formic acid in acetonitrile/water (60:40, v/v) and MF(B) was 5 mmol/L ammonium formate and 0.1% formic acid in isopropanol/water (90:10, v/v). A Reprospher Si column (10 × 2.1 mm, 1.7 μm, Dr. Maisch, Ammerbuch-Entringen, Germany) was used in the second dimension under the following conditions: flow rate 5 mL/min, column temperature 50 °C; mobile phase gradient (repeated for each fraction) 0 min–3% MF(B)_D2_, 0.15 min–50% MF(B)_D2_, 0.30 min–50% MF(B)_D2_, 0.31 min–3% MF(B)_D2_, and 0.55 min–3% MF(B)_D2_, where MF(A)_D2_ was pure carbon dioxide and MF(B)_D2_ was 30 mmol/L ammonium formate in methanol +1% of water. The back pressure regulator was set to 250 bar. In addition, a makeup flow splitter was connected behind the ^2^D column to enhance the ionization. MeOH with 30 mmol/L ammonium formate and 1% of H_2_O was used as a makeup solvent at a flow rate of 0.3 mL/min. The makeup flow was added isocratically from the binary pump of the second dimension system to SFC-MS Splitter Kit from Agilent Technologies The splitter was connected to the MS by a PEEKsil capillary (50 μm ID, 29.5 LG; Waters, Milford, MA, USA).

The second dimension gradient was controlled via a sequence of ChemStation software. Two identical methods were created, differing in the position of the valve that transferred the eluate from the first to the second dimension (position 1 → 3 or 1 → 7). In the sequence, methods with different valve positions were alternated, thereby the valve was turned, and the fraction was injected on the second dimension column at the same time as the start of the ^2^D gradient.

The hybrid quadrupole–time-of-flight (QTOF) Xevo G2-XS mass spectrometer (Waters) was used with the following parameters: sensitivity mode, capillary voltage 3 kV in the positive-ion mode and −1.5 kV in the negative-ion mode, sampling cone 20 V, source offset 90 V, source temperature 150 °C, desolvation temperature 500 °C, cone gas flow 50 L/h, desolvation gas flow 1000 L/h, acquisition range *m*/*z* 150–1200, scan time 10 Hz, and continuum profile mode. Peptide leucine enkephalin was used as the lock mass. MS/MS spectra were obtained by using MS^E^ and fast data-dependent analysis (DDA) approaches using argon as the collision gas. MS^E^ in continuum mode handling high energy collision ramp from 20 to 35 eV. The low collision energy was switched off to obtain nonfragmented data. Fast DDA was used for analyzing the fatty acyl composition mainly of triacylglycerols (TG), and the fragmentation of the five most intensive precursors was triggered at a collision ramp energy of 20–35 eV for low mass ramp and high mass ramp settings.

### Data Processing

The method acquisition and data evaluation were performed using MassLynx software (Waters) with subsequent noise reduction using the Waters Compression Tool. To reach higher mass accuracy, the lock mass correction was applied, and the data were converted from continuum to centroid mode using the Accurate Mass Measure tool in the MassLynx software. The peak areas were exported using the QuanLynx tool with tolerances for *m*/*z* ± 15 mDa and for retention times ±0.5 min. MS/MS spectra were measured without lock mass correction. The 2D data visualization was performed by GCImage (University of Nebraska, Lincoln, NE, USA). The plots were constructed using GraphPad Prism (version 10.2.1, GraphPad Software, Boston, MA, USA).

## Results and Discussion

### Development of RP-UHPLC Method for the First Dimension

The optimization in the first dimension is crucial for effectively distinguishing the analytes. This involves careful selection of an appropriate separation technique and optimization of its conditions, including the mobile phase composition (compatibility with the second dimension), gradient elution, flow rate, and suitable column parameters (length, diameter, and stationary phase). For the comprehensive 2D coupling, a relatively long column (e.g., 150 mm) packed with sub-2 μm particles and a small diameter (e.g., 1 mm) is commonly chosen in ^1^D. These parameters are essential to ensure a low flow rate of the mobile phase (typically below 50 μL/min), which must be optimized to achieve a sufficiently small peak volume and allow its division into at least two fractions in the second dimension.

The RP-UHPLC method used in the ^1^D was developed based on our previously published work.^23^ The mobile phase consisted of water–acetonitrile-2-propanol, enhanced with ammonium formate and formic acid, to achieve sufficient separation selectivity and improved ionization in the ion source of the mass spectrometer. The gradient program was adjusted to match the column parameters and a low flow rate of 8 μL/min. For the optimization of chromatographic conditions, a mixture of 26 lipid standards (StdMix) from 14 lipid classes was used, including various polar and nonpolar species (Table S1). Two different columns were tested for their separation performance of the lipid standard mixture and maximum possible injection volume: YMC Triart C18 column (150 × 0.5 mm; 1.9 μm) with fully porous particles, and Kinetex XB-C18 column (150 × 0.5 mm; 2.9 μm) filled with core–shell particles. For both columns, injection volumes from 0.1 to 0.8 μL were evaluated. The YMC Triart column offered better peak shapes with a higher possible injection volume (0.5 μL). The capacity of the Kinetex XB-C18 column was exceeded at the injection volume of 0.3 μL, leading to band broadening, reduced retention, and partial elution in the dead volume. The comparison of the effect of injection volume for six representative polar and nonpolar lipid standards is illustrated in Figure S2A–F. Other standards showed the same trend (not shown).

### Development of UHPSFC Method for the Second Dimension

UHPSFC with scCO_2_ as the main component of the mobile phase provides unique selectivity and faster separation than conventional liquid chromatography. In addition, the compatibility of scCO_2_ with the modifier (most commonly methanol with the addition of water and ionic additives) allows the analysis of compounds across a wide range of polarities. The isocratic elution offers more stable and reproducible conditions for separation in ^2^D and can simplify the setup and maintenance of the chromatographic system because it does not require gradient programs or additional gradient generation hardware. However, gradient elution effectively separates analytes with different polarities, from very polar to very nonpolar compounds, and shortens the overall analysis time by eluting strongly bound analytes more quickly.

Based on the peak widths in the first dimension, the separation in the second dimension had to be completed in about 0.5 min. This time includes both the gradient and subsequent equilibration of the column before injection of the next fraction from the first dimension. Only a very short column allows a fast analysis with high flow rate (4–5 mL/min), such as the Reprospher Si column (10 × 2.1 mm; 1.7 μm).

Another issue was the volume of fractions and the volume of loops in the modulator transferred between dimensions. The suitable fraction volume is important to achieve the best possible separation and resolution in the second dimension as the mobile phases in RP and SFC are poorly compatible. The mobile phase in SFC is pressurized throughout the system to keep carbon dioxide in a condensed form. When the modulator is switched, the pressure is lost (the loop is behind the column of the first dimension), and carbon dioxide is evaporated. Subsequently, this empty loop is filled with eluate from the first dimension, and the size of the loops must correspond to the size of the fraction. With a much larger volume of loops than the fraction, a significant amount of gas would be injected into the SFC system, together with the sample.

First, the fast gradient on the short ^2^D column was optimized using the standard mixture. The total gradient time was 0.5 min, including 0.2 min for equilibration of the column. Then, the optimal ^2^D injection volume was investigated by injecting from 1 to 8 μL of standards. The StdMix was dissolved in a mixture of solvents that corresponds to the composition of the mobile phase effluent from the first dimension at different separation times. The composition of solvents is described in Table S2. Initially, the Acquity UPC^2^ system was used for the UHPSFC method development. Later, Acquity UPC^2^ system was replaced by the Agilent SFC system. The effect of the injection volume on individual lipid classes exhibited varying behavior. While standards from the group of polar lipids (e.g., phospholipids or sphingolipids) showed no issues even at higher injection volumes, nonpolar lipids [e.g., cholesterol esters (CEs) or acylglycerols] experienced peak broadening and exceeded the column capacity at an injection volume of 3 μL (Figure S3A–F). Due to the availability of 5 μL loops, the mobile phase flow rate of 8 μL/min from the first dimension, and the sampling time of 0.55 min, a 5 μL injection volume was selected as the optimal choice.

### RP-UHPLC × UHPSFC/MS/MS Connection

The biggest challenge in method development was connecting two different chromatographic systems from two different manufacturers (^1^D Agilent and ^2^D Waters). The second dimension of the 2D system was initially tested using a Waters SFC chromatographic system, on which the optimization of the second dimension was performed. The connection had to ensure that the fast gradient of the mobile phase in the second dimension occurred every 0.5 min, while simultaneously having the modulator controlled by the first dimension switch at the start of the ^2^D gradient. If the start time of each gradient in the second dimension did not coincide with the change in the modulator position, it would result in the shift of retention times in the second dimension and the change in the ^2^D separation. Furthermore, the collection of fractions was not be repeatable. Unfortunately, this SFC system could not be used due to its pressure limit (6000 psi, 413 bar) at the maximum flow rate (4 mL/min), the inability to achieve the separation in less than 1 min in the second dimension, and the problematic gradient synchronization with the modulator switching. Another option was to connect the Agilent 1260 Infinity SFC system. The Agilent SFC system allowed flow rates of up to 5 mL/min at the pressure of 600 bar. The disadvantage was a larger gradient delay compared to that of the Waters SFC system, which was reduced by disconnecting the mixer and using the shortest possible capillaries. Moreover, the valve specifically designed for 2D chromatography from Agilent was used, which allows for easy control of 2D gradients.

Initially, we planned the integration of UHPLC and SFC into a single system controlled by one ChemStation implementation, including 2D-LC add-on software. Unfortunately, 2D-LC add-on software did not support the use of the SFC pump in the second dimension. Finally, two ChemStation software setups were created. The first one controlled the ^1^D pump and the autosampler, while the second one was configured to manage the ^2^D SFC pump, the SFC control module, the SFC makeup flow, and the column thermostat containing the 2D-LC valve equipped with sampling loops. The simultaneous switching of the modulator and the start of the gradient in the second dimension for each fraction was solved using two methods differing only in the position of the 2D-LC valve (1 → 3 or 1 → 7). These methods were alternated in one sequence (200 methods in sequence).

To ensure precise and reproducible analyses, the back pressure value was optimized (Figure S4A). The value of 250 bar was found to best maintain a consistent pressure within the chromatographic system, providing the highest analyte response. The high-resolution Xevo G2-XS QTOF mass spectrometer was used for the untargeted analysis of lipid species. Individual parameters were extensively investigated in relation to the ^2^D conditions, including ion source temperature, desolvation temperature, cone gas flow rate, and desolvation gas flow. For the comprehensive analysis of human plasma samples, two different approaches to data collection were implemented. Data-independent analysis, provided by MS^E^ scanning mode, allows for the simultaneous acquisition of data at both low and high collision energies within a single analysis cycle, capturing information on molecular ions and their fragments. This approach is particularly useful in metabolomics and lipidomics, where identifying and quantifying a large number of substances in a single analysis is essential.^43^ For this purpose, the high collision energy of 25 eV was selected as the optimal value for fragmentation of a wide range of lipid species in the lipidomic analysis.^23^ To prevent the fragmentation of more labile analytes, the low fragmentation energy setting was turned off. During DDA represented by the fastDDA mode, the software selects ions for fragmentation based on their abundances, focusing on the most relevant analytes.^43^ For a more detailed characterization of TG, the fragmentation was set to target the five most abundant ions. This approach led to the distinction of individual TG species and its fragments. To ensure optimal ionization in the source, an SFC-MS Splitter Kit was added, delivering a flow of makeup solvent with the same composition as the modifier used in the second dimension. Various flow rates were tested (Figure S4B). Polar lipids ionized best at the lowest flow rates tested, with the ionization efficiency decreasing as the flow rate increased. In contrast, nonpolar lipids experienced inadequate ionization at low flow rates, with the ionization efficiency improving as the flow rate increased. The flow rate of 300 μL/min was determined to be optimal for the full range of polarities. Another approach to improve ionization was to establish the optimal capillary voltage in the ion source (Figure S4C). The voltage adjustment had the most significant impact on nonpolar lipids, where the response decreased with an increasing voltage. For polar lipids, the changes in ionization were negligible. The optimal voltage of 3 kV was selected.

Multidimensional chromatography offers a significant advantage in achieving a much higher peak capacity compared with one-dimensional chromatography, allowing for better resolution of complex mixtures. The effective peak capacity of our newly developed multidimensional method was calculated according to the procedure described by Davis et al.,^44^ which included the effect of the undersampling of peaks from the first dimension. Compared to our previously published methods,^23,29^ the RP-UHPLC × UHPSFC approach demonstrates over 10-fold increase in the peak capacity compared to RP-UHPLC (526 vs 50) and 18-fold increase over UHPSFC (526 vs 29). One drawback of the new method is the longer analysis time, which is nearly five times that of the routinely used RP-UHPLC method. Another limitation is the sample dilution in two dimensions, which leads to a decrease in the sensitivity. This issue may be mitigated in the future through the use of trapping columns between dimensions or by employing the active solvent modulation method.^45^ Despite these challenges, the two-dimensional approach offers a key advantage in higher confidence in identification, thanks to retention times in both dimensions.

### Lipidomics Application of RP-UHPLC × UHPSFC/MS/MS

The optimized comprehensive RP-UHPLC × UHPSFC/MS/MS method allows a combination of both the lipid class and lipid species analysis. In total, 298 lipid species from 15 lipid classes were identified, including TG, diacylglycerols (DG), fatty acids (FA), phosphatidylcholines (PC and PC P-), lysophosphatidylcholines (LPC), phosphatidylethanolamines (PE and PE P-), phosphatidylinositols (PI), sphingomyelins (SM), ceramides (Cer), hexosylceramides (HexCer), dihexosylceramides (Hex_2_Cer), monosialodihexosylgangliosides (GM3), CE, and cholesterol (Chol).

**Figure 2:**
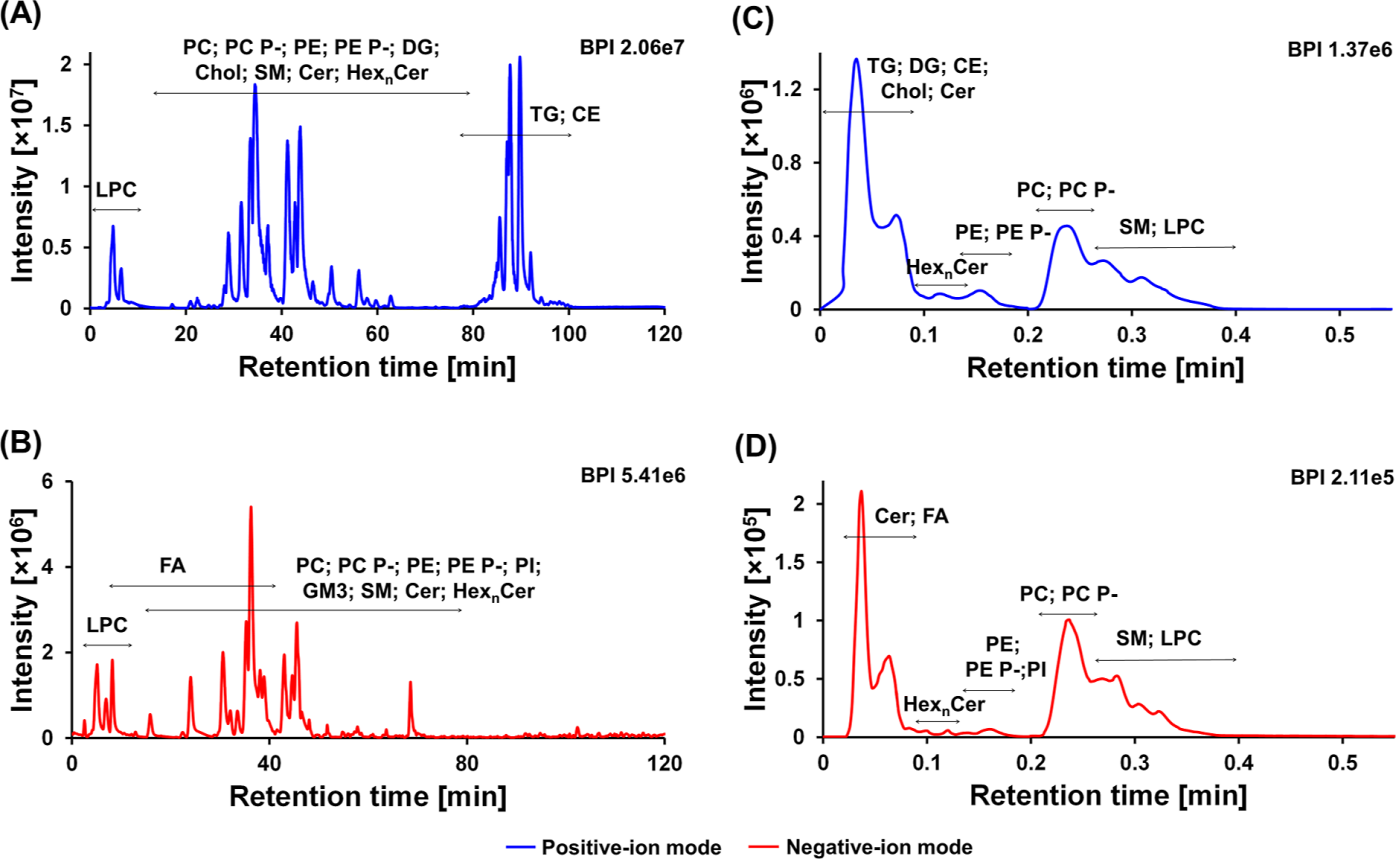
ESI-MS base peak chromatograms of human plasma showing the retention of individual lipid subclasses: (A) RP-UHPLC method in the positive-ion mode, (B) RP-UHPLC method in the negative-ion mode, (C) UHPSFC method in the positive-ion mode, and (D) UHPSFC method in the negative-ion mode.

Lipid separation in the lipid species separation approach can be characterized by the equivalent carbon number (ECN), which is calculated as the total number of carbons in all fatty acyl chains minus twice the number of double bonds (ECN = CN–2DB).^23^ This method effectively characterizes lipids by considering both the chain length and the degree of unsaturation within the molecular structure. The most polar lipids (LPC, FA) are eluted at the beginning of the chromatogram, followed by polar and less polar lipids (Chol, SM, GM3, Hex_n_Cer, Cer, PI, PC, PE, and DG), and finally nonpolar lipids (TG and CE). Figure 2 shows the base peak intensity chromatogram of a human plasma sample in positive-ion (A) and negative-ion (B) modes. The chromatograms of lipid standards are shown in Figure S5A,B.

In UHPSFC, the lipids are separated according to the polarity of the headgroup (lipid class separation approach), resulting in the coelution of all lipid species within one lipid class in one chromatographic peak. Nonpolar lipid classes (TG and CE) together with cholesterol are eluted close to the void volume of the system, while polar lipid classes (PE, PC, SM, and LPC) exhibit acceptable separation according to their polarity. The analysis of human plasma using the UHPSFC/MS method is illustrated in Figure 2C,D. Figure S6A,B shows the chromatographic separation of lipid standards.

For the identification of lipids in human plasma samples, the characteristic precursor and fragment ions were searched in both positive- and negative-ion modes using fast DDA and MS^E^ approaches. The ionization and fragmentation behavior of individual lipid species was described in our recently published papers.^22−24,46^ The list of identified analytes is summarized in Table S3, including their retention times and characteristic ions in MS and MS/MS modes along with mass accuracy. DG, TG, CE, and Chol are detected only in the positive-ion mode, while PI, GM3, and FA are observed only in the negative-ion mode. The shorthand notation and nomenclature of lipids follow the updated guidelines by Liebisch et al.^13^ The characteristic adduct and fragment ions observed for individual lipid classes are detailed in Table S4, and raw annotated tandem mass spectra of selected isobaric molecules are given in Figure S7.

**Figure 3:**
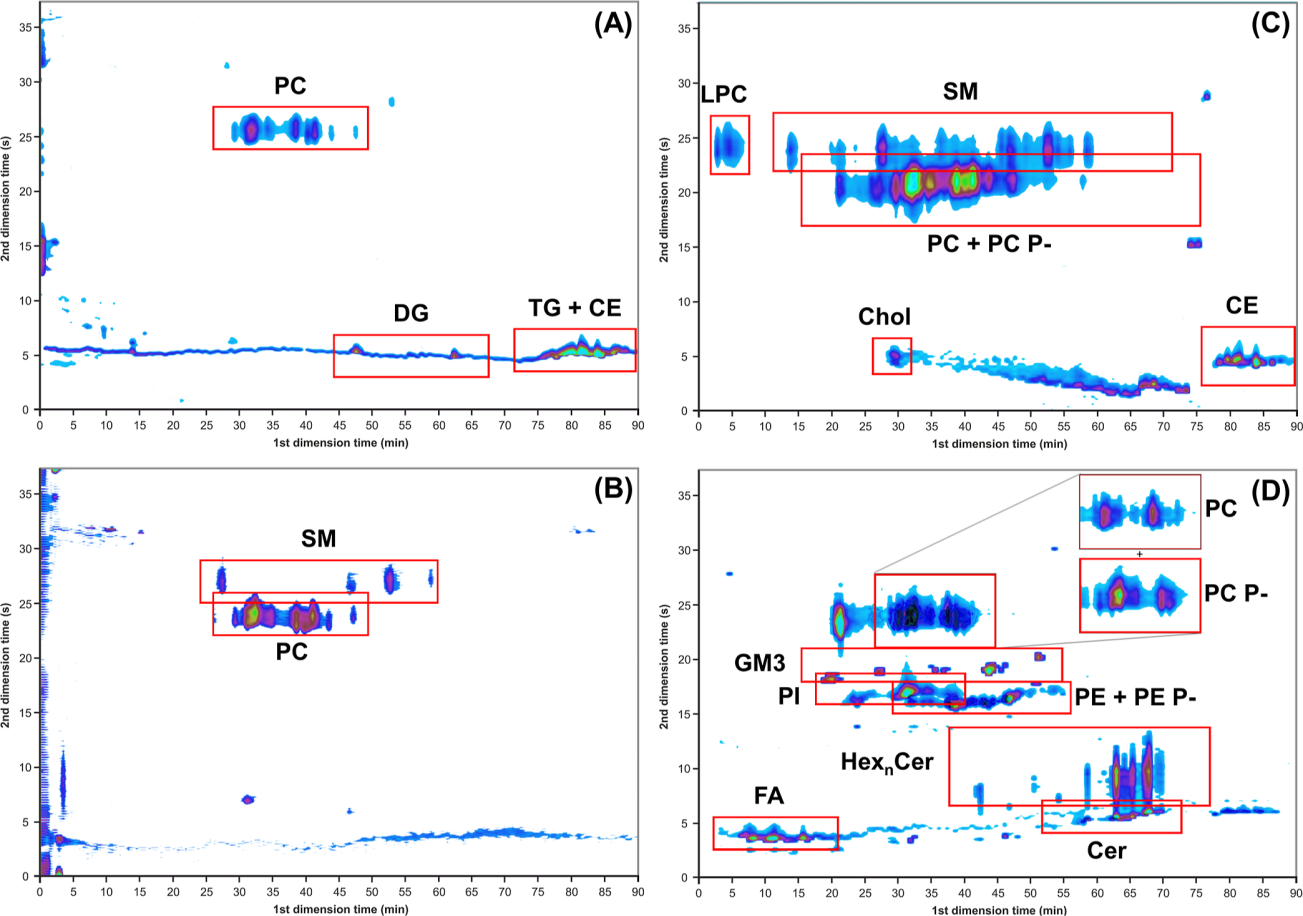
Multidimensional separation of lipid extract from human plasma samples with the final method conditions showing: base peak chromatograms in positive- (A) and negative (B)-ion modes, RIC chromatograms of *m*/*z* 184 characteristic for PC, SM, and LPC classes, and *m*/*z* 369 characteristic for cholesterol and CE using the positive-ion mode (C), and RIC of low abundant lipid classes measured in the negative-ion mode (D), including FA, Cer, HexCer, Hex_2_Cer, GM3, PE and PE P-, PI, and PC P-.

GCImage software was used for the visualization of 2D chromatograms, where red and green dots correspond to the most abundant species and blue correspond to species of lower abundance. Figure 3 shows the base peak chromatogram in positive- (A) and negative- (B) ion modes, but only the most abundant lipids (TG, some DG, CE, PC, and SM) are visible because human plasma contains lipids at widely varying concentrations.^47^ For better visualization, individual reconstructed ion current chromatograms (RICs) of selected compound classes were produced. Figure 3C highlights the lipid classes that produce the precursor ion *m*/*z* 184, which is characteristic for the molecules containing a phosphatidylcholine part (LPC, PC, and SM), and *m*/*z* 369, which is characteristic for cholesterol and CE, measured in the positive-ion mode. Figure 3D illustrates the RIC of lower abundant lipids measured in the negative-ion mode including FA, Cer, HexCer, Hex2Cer, GM3, PE and PE P-, PI, and PC P-.

## Conclusions

This work demonstrates the development of a novel comprehensive online multidimensional RP-UHPLC × UHPSFC/MS/MS method for the analysis of lipid extracts from biological samples. In lipidomics, a logical combination involves the use of lipid class separation (HILIC or NP-UHPLC) in one dimension and lipid species separation (RP-UHPLC) in another dimension. Compared to typical 2D conditions in previously published 2D-LC papers, the low viscosity and high diffusivity of scCO_2_, used as the mobile phase in UHPSFC, offer the advantage of faster analysis in the second dimension. In the first dimension, the RP-UHPLC method with 150 mm C18 column and sub-2 μm particles was used to separate lipids based on their polarity, fatty acyl chain length, and number of double bonds. Coeluting lipids were further separated in the second dimension using UHPSFC, employing 10 mm silica column and the flow rate of 5 mL/min. Individual fractions eluting from the first dimension were accumulated in two 5 μL loops and subsequently transferred to the second dimension via controlled switching of 2-position/4-port-duo valve interface. Connecting this configuration to the mass spectrometer capable of DDA and DIA scanning modes added two further dimensions to the analytical system. The present work is a proof of concept of the possible application of continuous comprehensive multidimensional chromatography in lipidomics, but the method still has limitations, including the need for more elegant solution for valve programming, which could be addressed by developing 2D-LC add-on software in ChemStation software that includes SFC pump control. Another issue is the sensitivity of the method, which requires large injection volumes and more concentrated samples. Nonetheless, we successfully identified 298 lipid species from both polar and nonpolar subclasses in human plasma samples, illustrating the potential of this approach for comprehensive lipidomic profiling.

## Supporting Information Available

Two possible positions of the valve, comparison of injection volumes, optimization diagrams, chromatograms of StdMix (PDF), row annotated mass spectra, composition of StdMix, composition of solvents for method optimization, list of identified lipid species, and characteristic ions (PDF)

## Notes

All volunteers signed informed consent, and the ethical committee approved the blood collection.
