# Supplementary material for: Four-Dimensional Lipidomic Analysis Using Comprehensive Online UHPLC × UHPSFC/Tandem Mass Spectrometry
Source: Anal Chem. 2024 Nov 27;96(49):19439–46. doi: 10.1021/acs.analchem.4c03946 (PMC11635755; doi:10.1021/acs.analchem.4c03946)
Supplement: Supplementary file 1 — ac4c03946_si_001.pdf [file ac4c03946_si_001.pdf]

SUPPORTING INFORMATION

Four-Dimensional Lipidomic Analysis Using Comprehensive Online UHPLC×UHPSFC/MS/MS

Zuzana Lásko[1,#,] Tomáš Hájek[1,#], Robert Jirásko[1], Ondřej Peterka[1], Petr Šimek[2], Peter J. Schoenmakers[3], Michal Holčapek[1*]

[1]*University of Pardubice, Faculty of Chemical Technology, Department of Analytical Chemistry, Studentská 573, 53210 Pardubice, Czech Republic*

[2]*Biology Centre of the Czech Academy of Sciences, České Budějovice, 370 05, Czech Republic*

[3]*University of Amsterdam, van 't Hoff Institute for Molecular Sciences, Analytical Chemistry Group, Science Park, 904, 1098 XH Amsterdam, The Netherlands*

* Corresponding author: Michal Holčapek, Tel.: +420466037087; Fax: +420466037068; Email: Michal.Holcapek@upce.cz

# Shared first authors.

Content:

Number of figures: 7

Number of tables: 4

Table of Contents:

Figure S1. Two positions of the 2-position/4-port duo valve 3

Figure S2. Effect of injection volume for RP-UHPLC method. 4

Figure S3. Effect of injection volume for UHPSFC method... 5

Figure S4. Optimization of UHPLC×UHPSFC/MS/MS conditions for selected lipid standards.. 6

Figure S5. Base peak chromatograms of the lipid standard mixture measured by RP-UHPLC/MS 8

Figure S6. Base peak chromatograms of the lipid standard mixture measured by UHPSFC/MS 9

Figure S7. Raw annotated tandem mass spectra of selected isobaric molecules 10

Table S1. Calculation of the lipid standard mix concentrations in body fluid. 52

Table S2. Compositions of the solvents used for optimization of maximal injection volume in second-dimension. 54

Table S3. List of lipid species identified in pooled human plasma sample with their retention time, and observed ions in positive and negative ESI mode...................... 55

Table S4. Characteristic ions of individual lipid classes used for the identification of lipid species observed in positive and negative ESI mode... 67

Figure S1. Two positions of the 2-position/4-port duo valve. (A) - position 1→7; (B) - position 1→3.

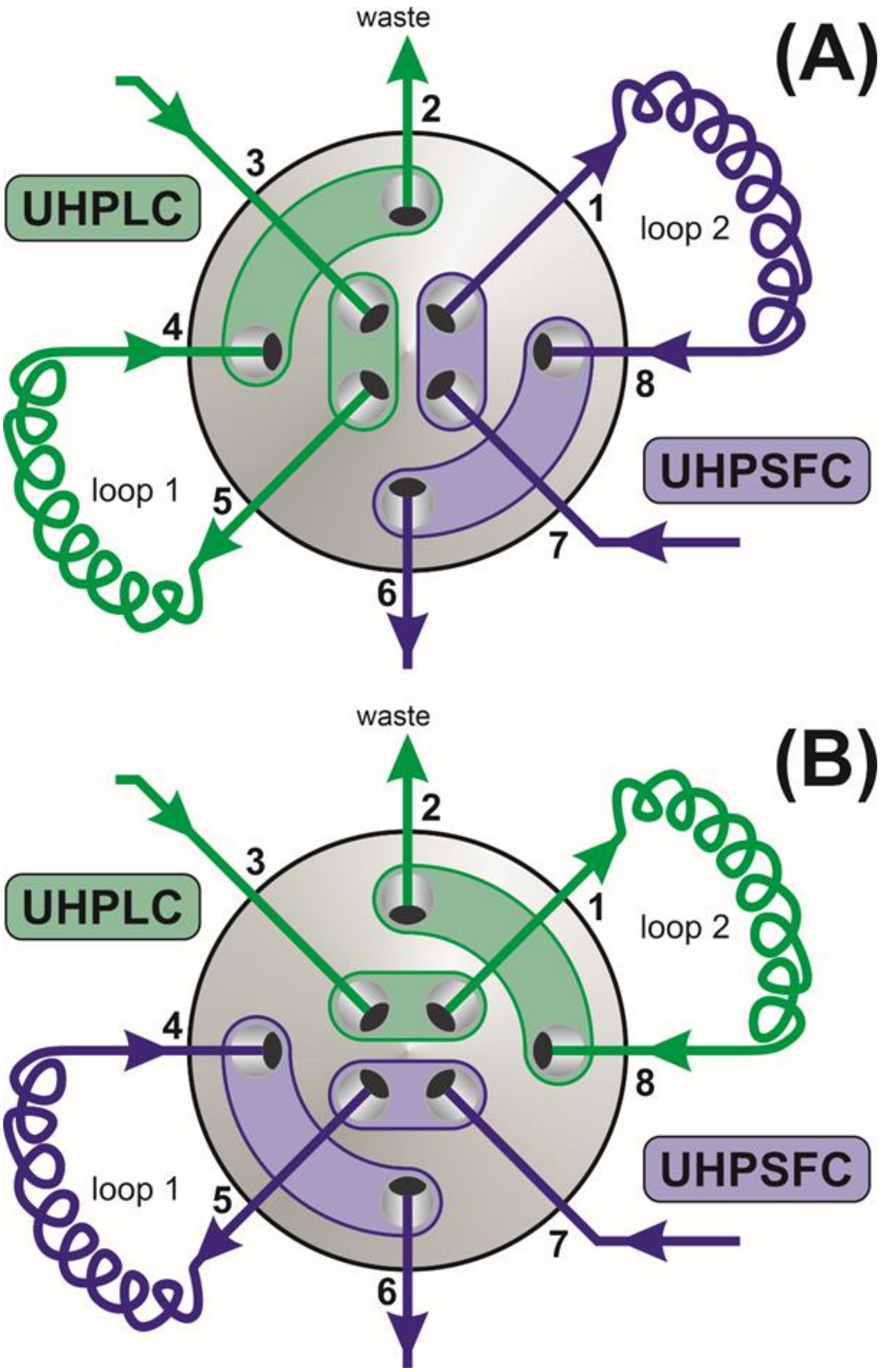

Figure S2. Effect of injection volume for RP-UHPLC method using YMC Triart C18 column (150×0.5 mm; 1.9 µm) for selected lipid standards: (A) LPC 13:0, (B) SM 18:1;O2/12:0, (C) PC 14:0/14:0, (D) DG 12:0/12:0, (E) CE 19:0, and (F) TG 19:1/19:1/19:1.

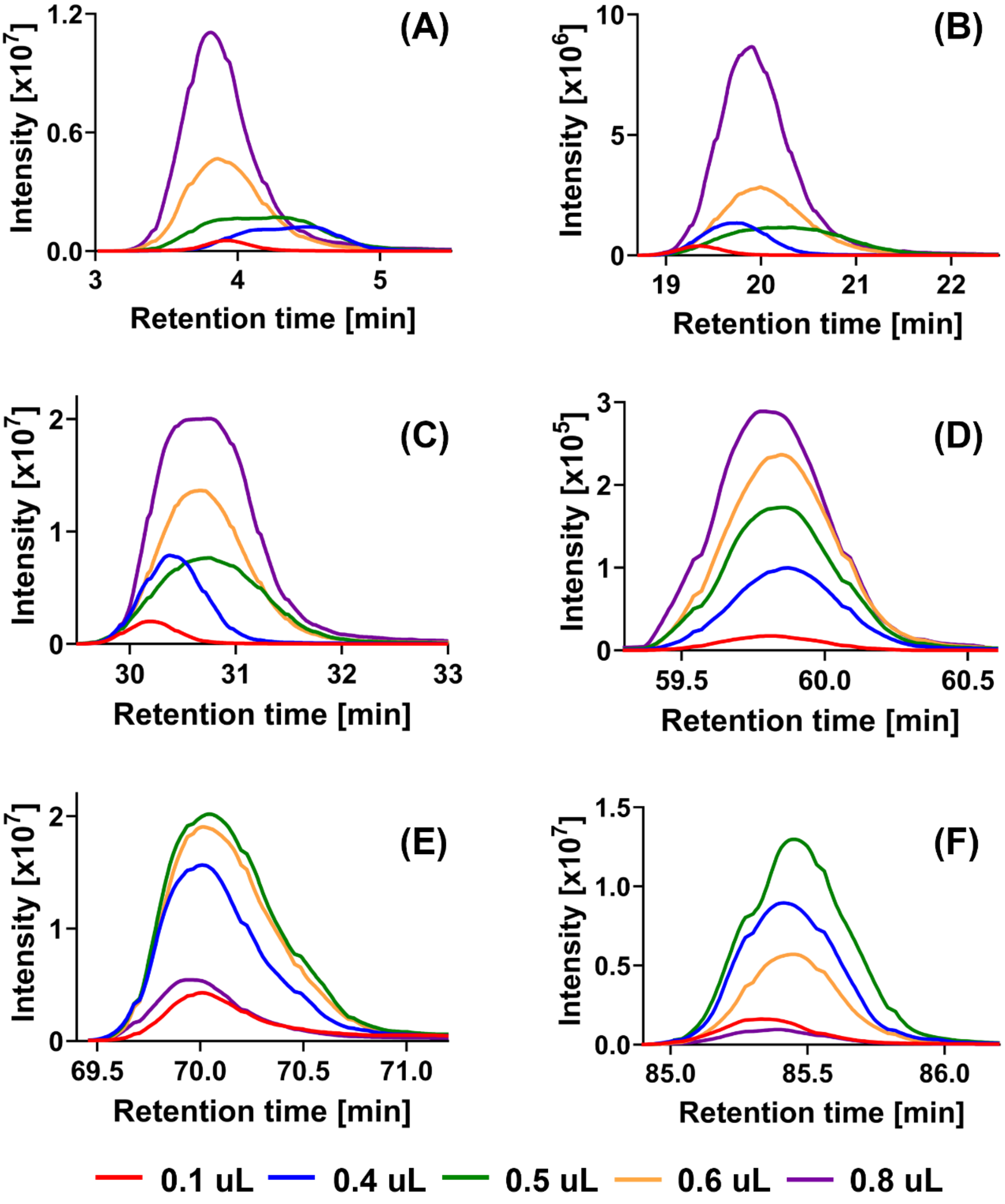

Figure S3. Effect of injection volume for UHPSFC method using Reprospher Si column (10×2.1 mm; 1.7 µm) for selected lipid standards: (A) LPC 13:0, (B) SM 18:1;O2/12:0, (C) PC 14:0/14:0, (D) DG 12:0/12:0, (E) CE 19:0, and (F) TG 19:1/19:1/19:1.

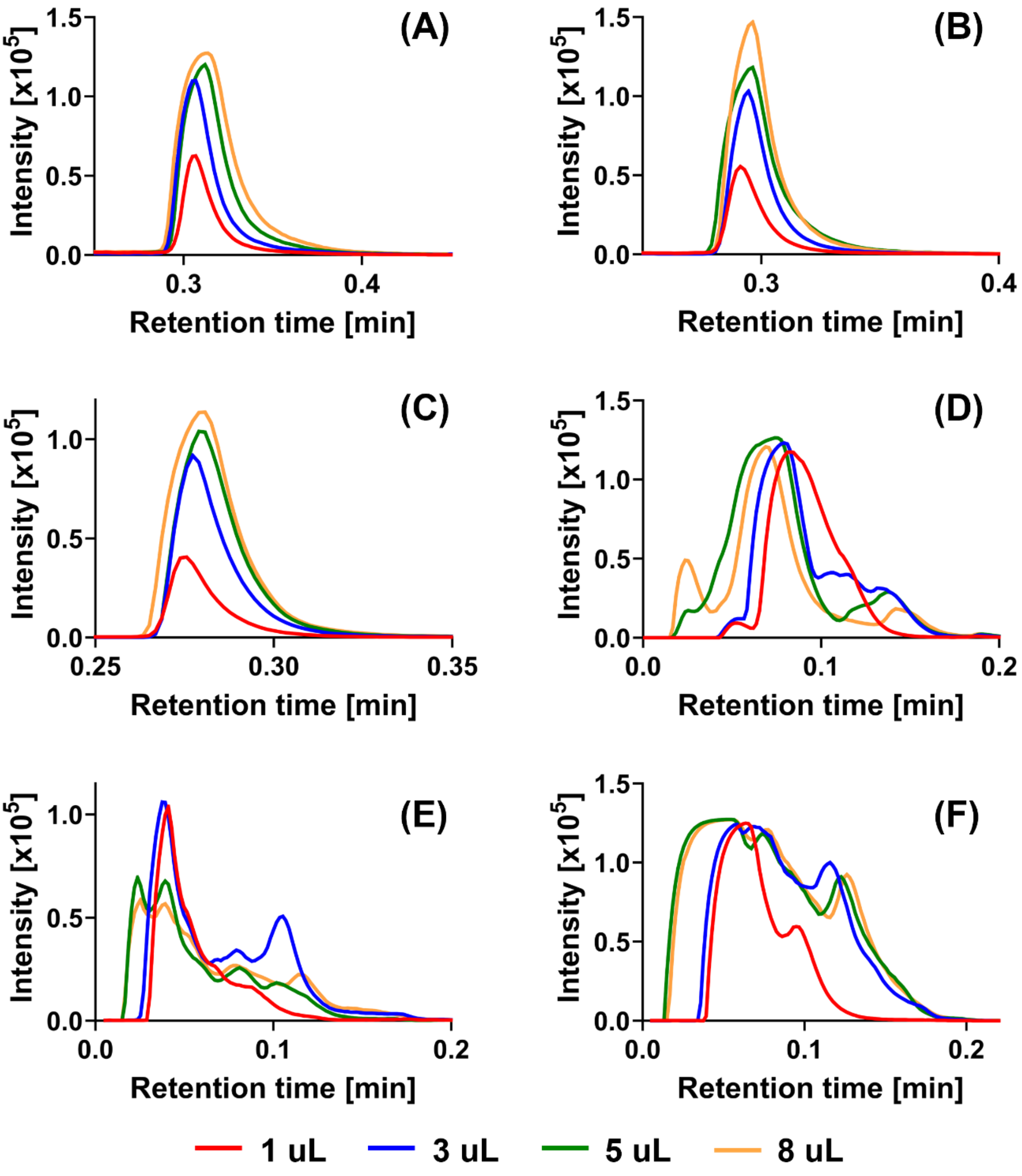

Figure S4. Optimization of UHPLC×UHPSFC/MS/MS conditions for selected lipid standards, whereas other standards follow the same trends: (A) UHPSFC back pressure, (B) make-up flow, and (C) ion source capillary voltage. Data are presented as the mean value ± SD from three independent experiments.

(A)

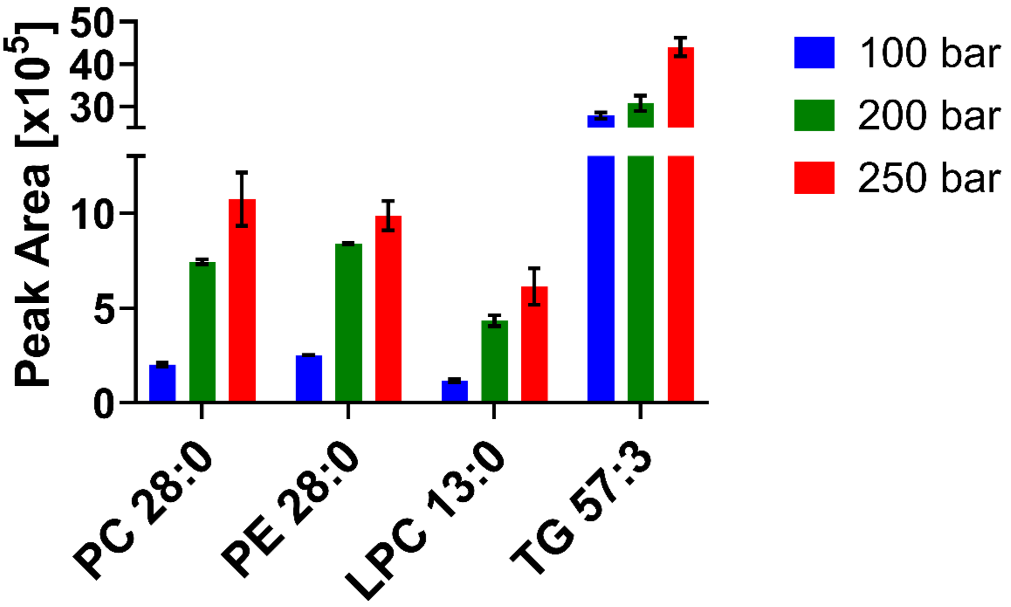

(B)

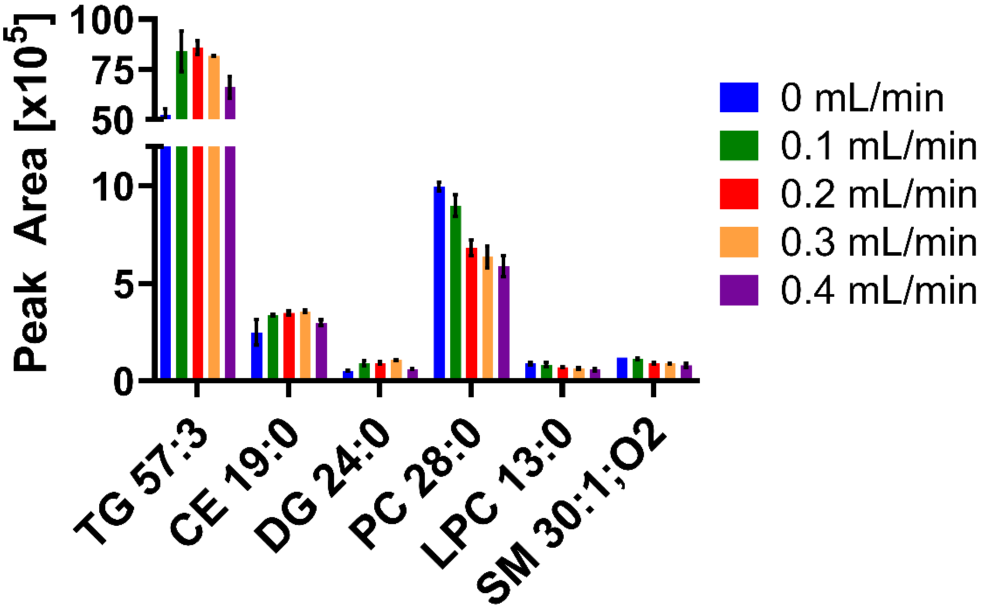

(C)

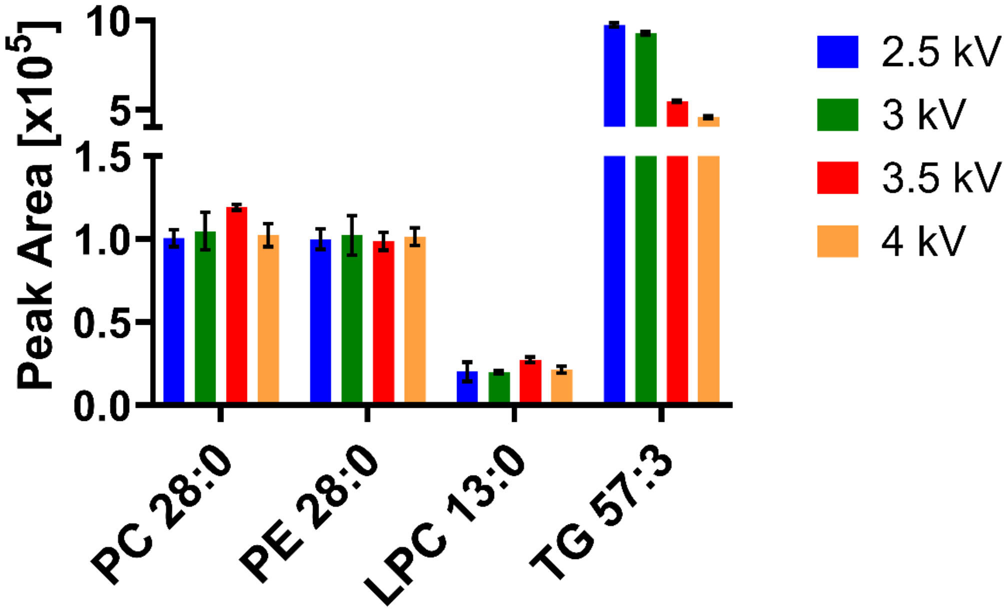
Peak Area [x10^5]
10
5
1.5
1.0
0.5
0.0
PC 28:0
PE 28:0
LPC 13:0
TG 57:3
2.5 kV
3 kV
3.5 kV
4 kV

Figure S5. Base peak chromatograms of the lipid standard mixture measured by RP-UHPLC/MS in: (A) positive ion mode and (B) negative ion mode. The conditions of analysis are reported in Materials and methods.

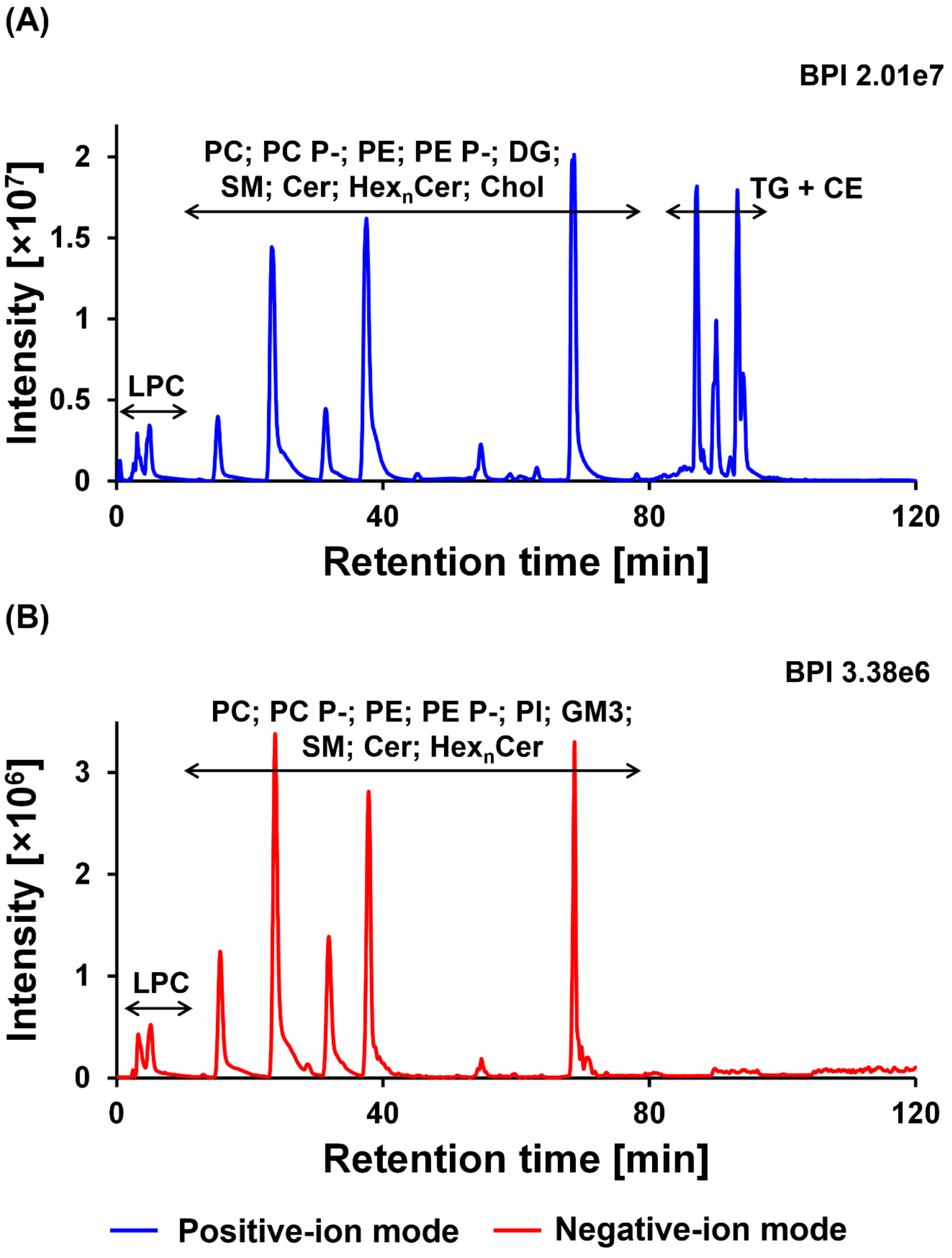

Figure S6. Base peak chromatograms of the lipid standard mixture measured by UHPSFC/MS in: (A) positive ion mode and (B) negative ion mode. The conditions of analysis are reported in Materials and methods.

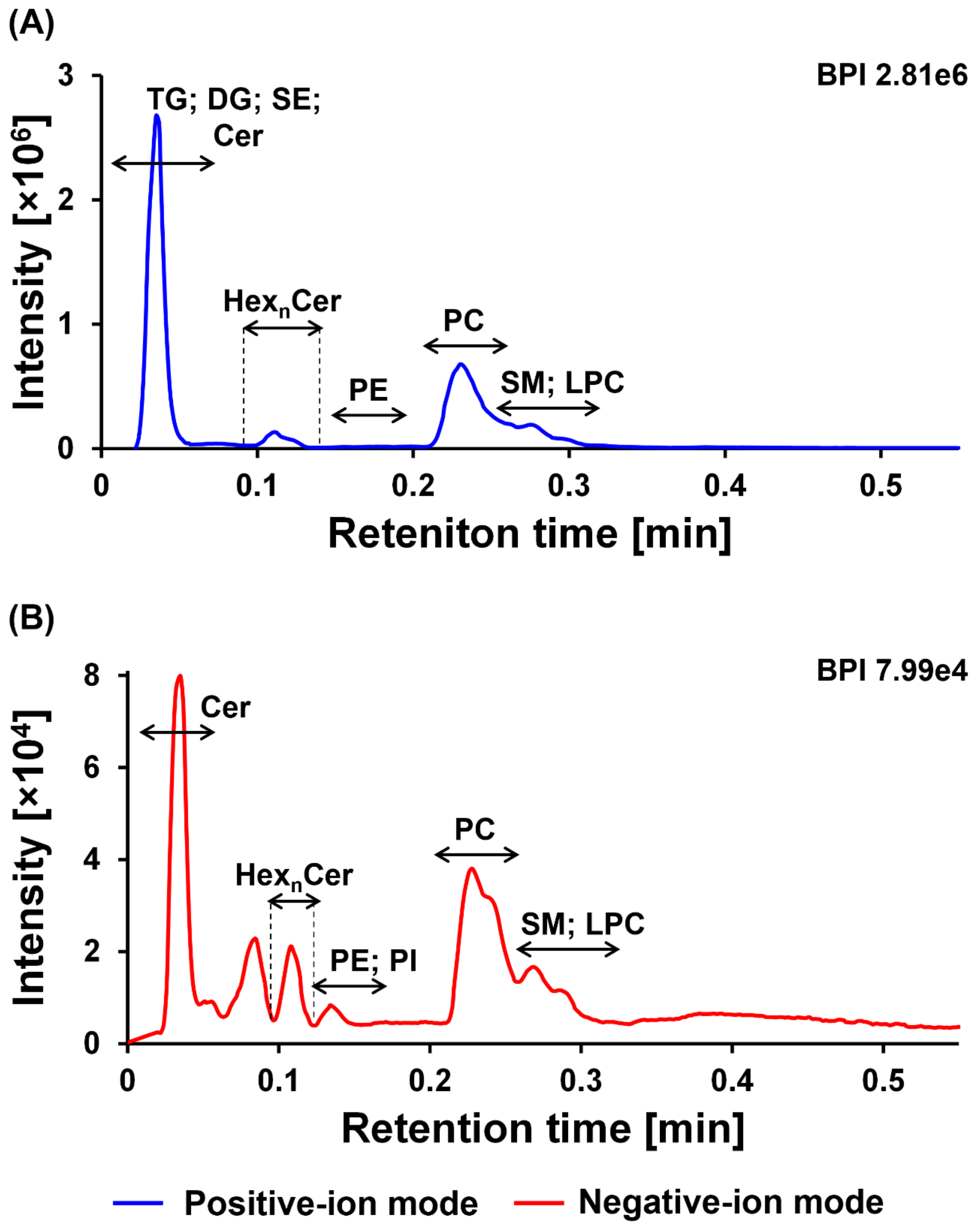

Figure S7. Raw annotated tandem mass spectra of selected isobaric molecules

PC 36:3; PC 16:0/20:3; ESI-, fast DDA

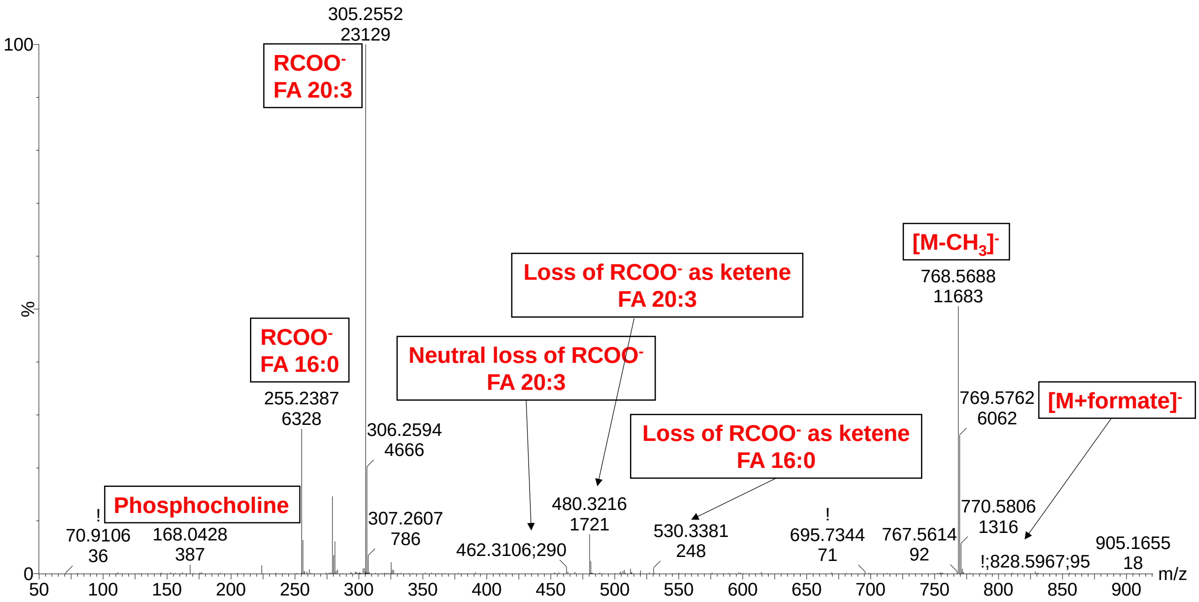

PC 36:3; PC 18:1/18:2; ESI-, fast DDA

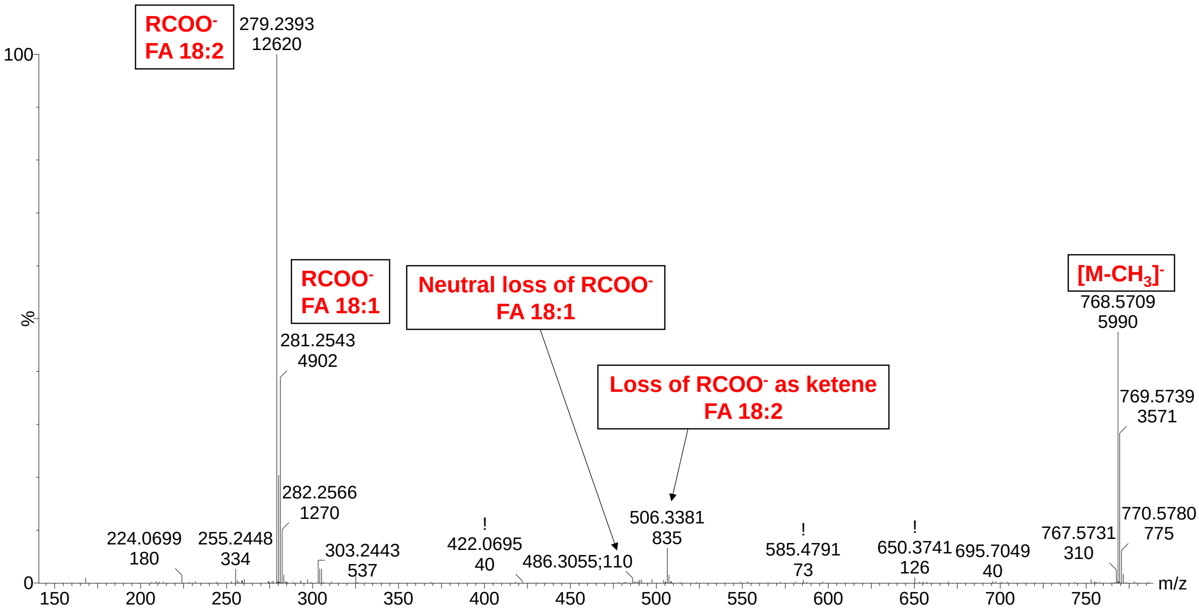

PC 36:4; PC 18:2/18:2; ESI-, fast DDA

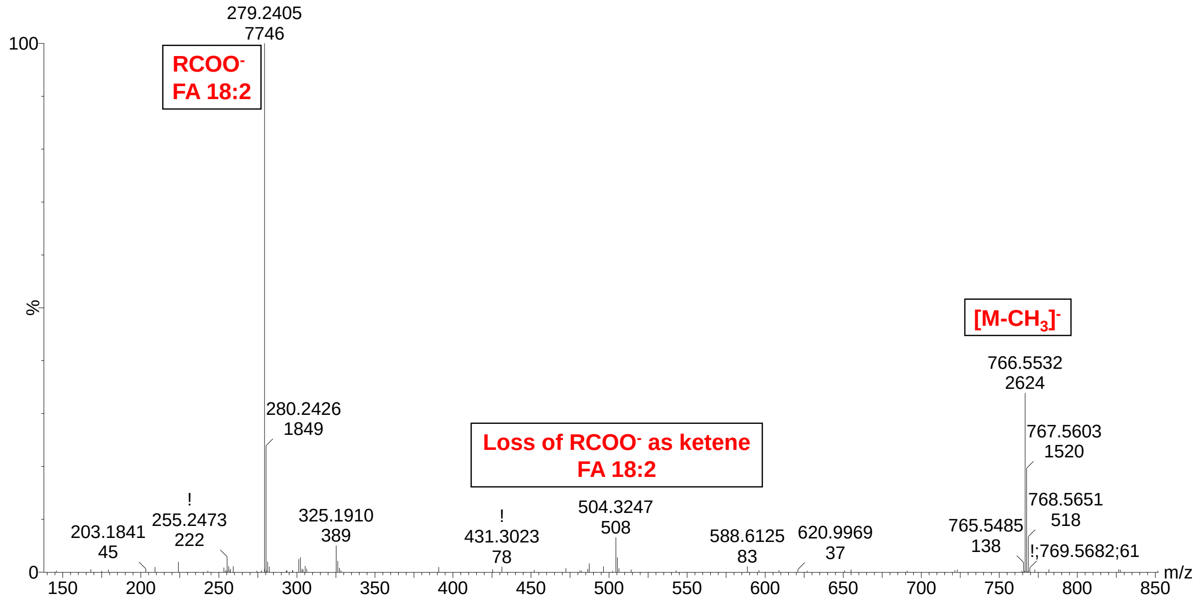

PC 36:4; PC 16:0/20:4; ESI-, fast DDA

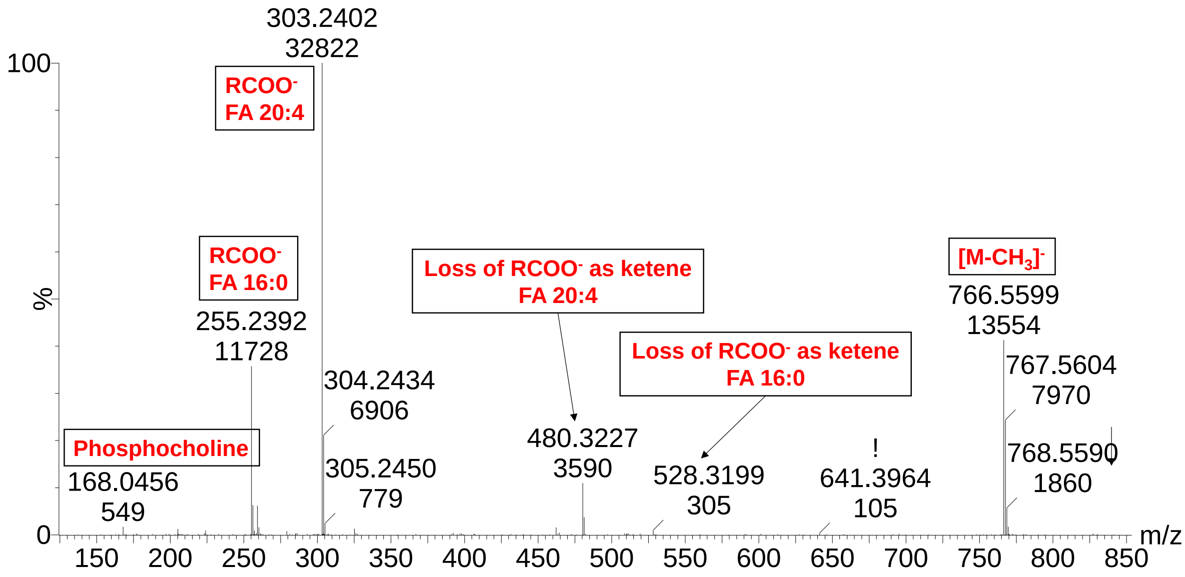

PC 38:4; PC 18:1/20:3; ESI-, MS^E

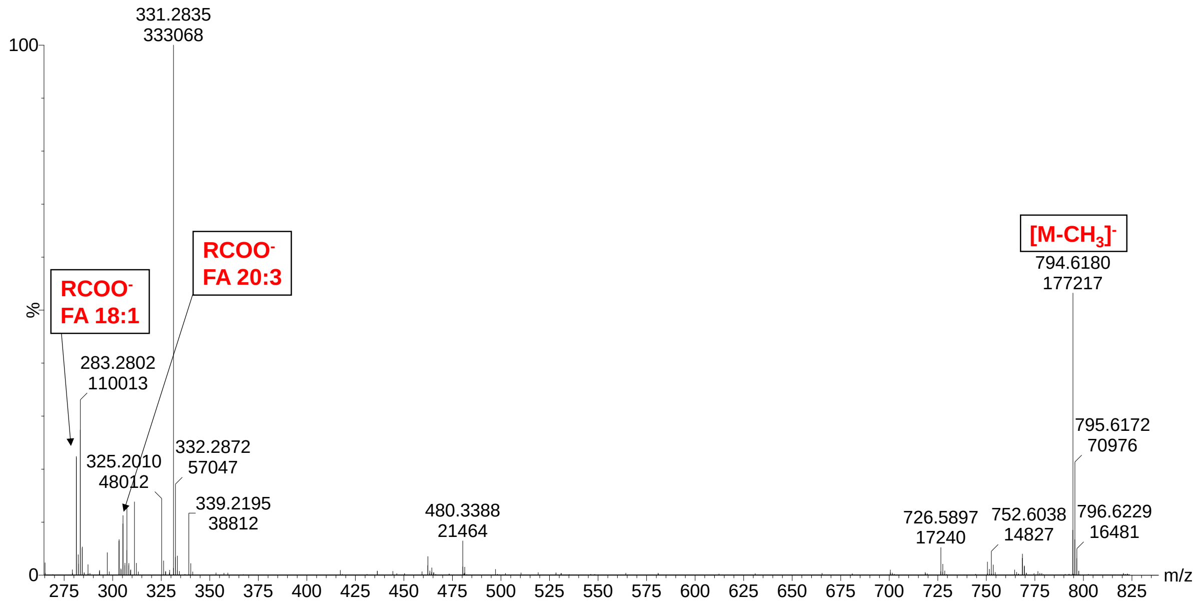

PC 38:4; PC 18:0/20:4; ESI-, fast DDA

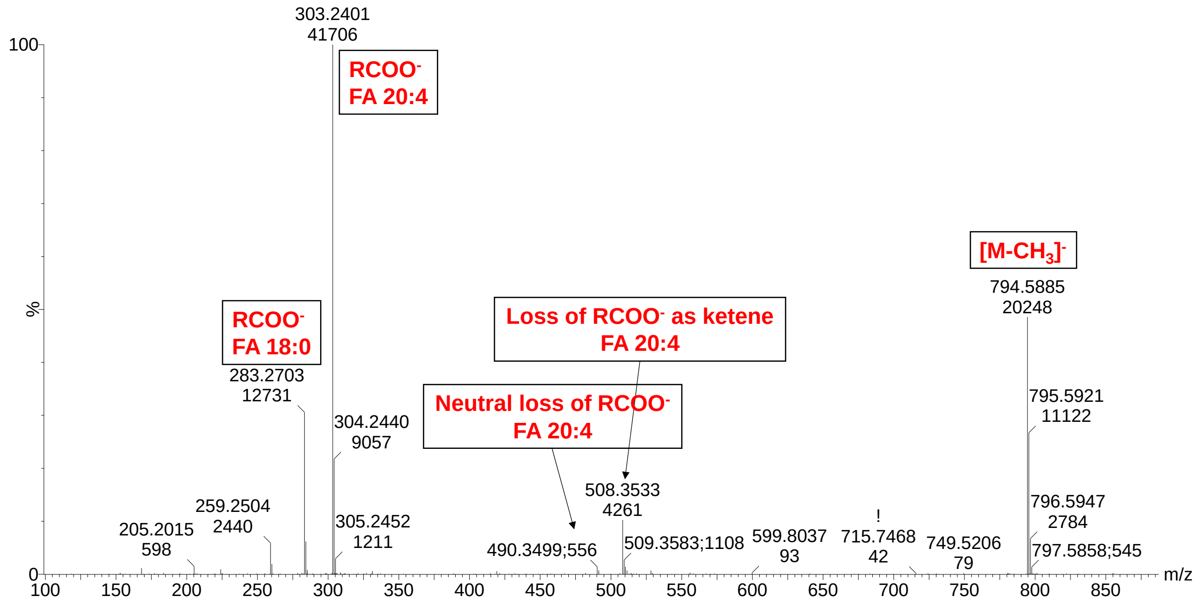

PC 38:5; PC 16:0/22:5; ESI-, fast DDA

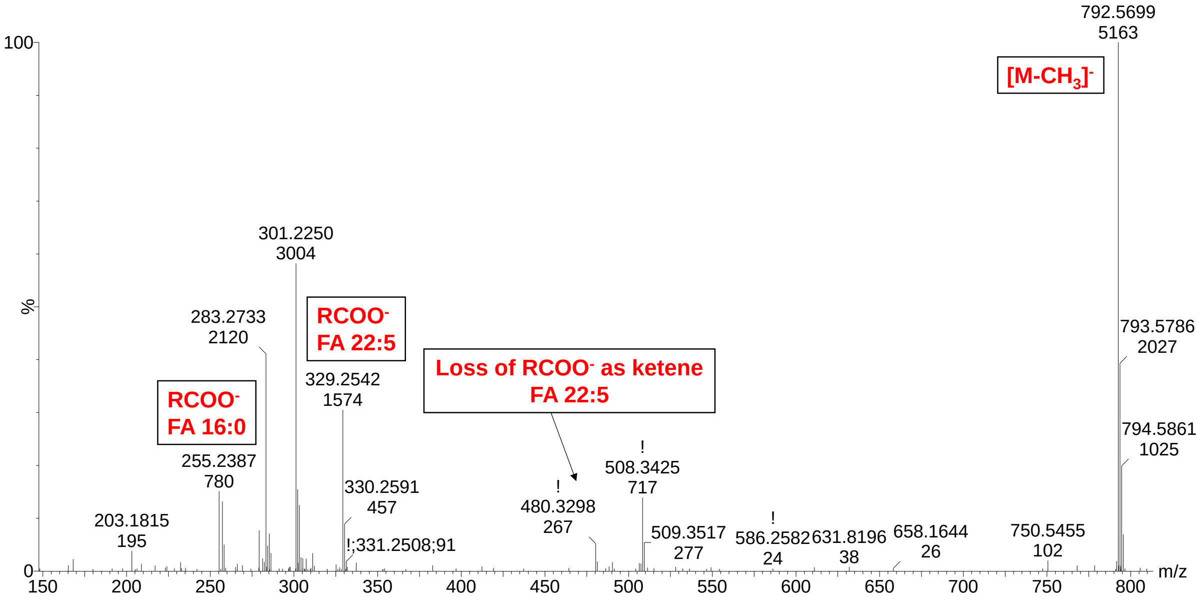

PC 38:5; PC 18:1/20:4; ESI-, fast DDA

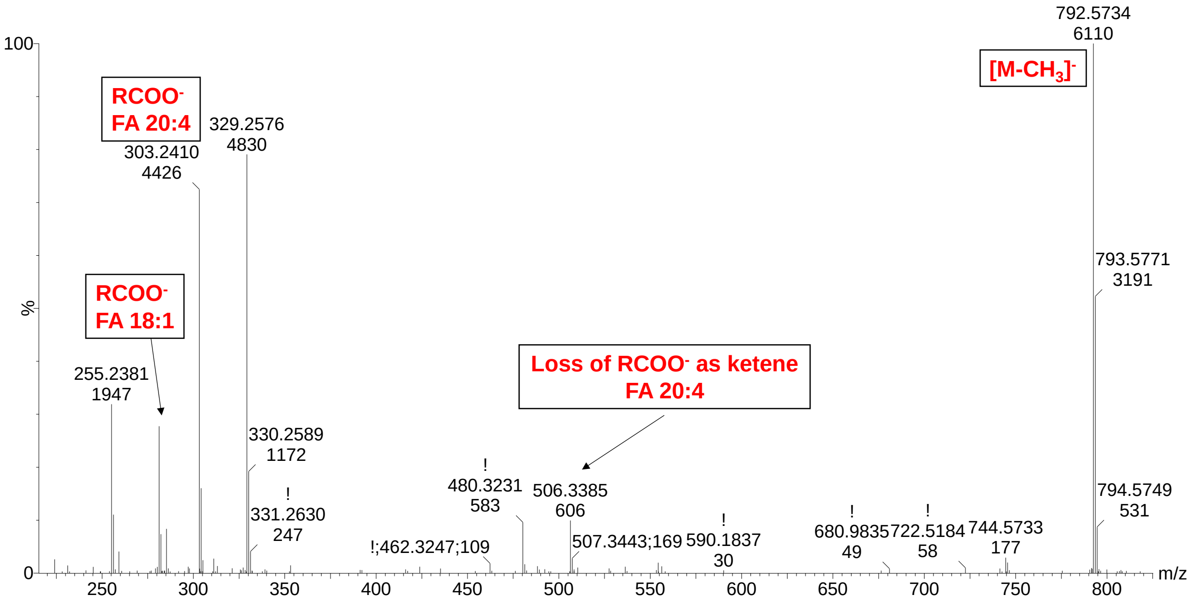

PC 38:5; PC 18:0/20:5; ESI-, fast DDA

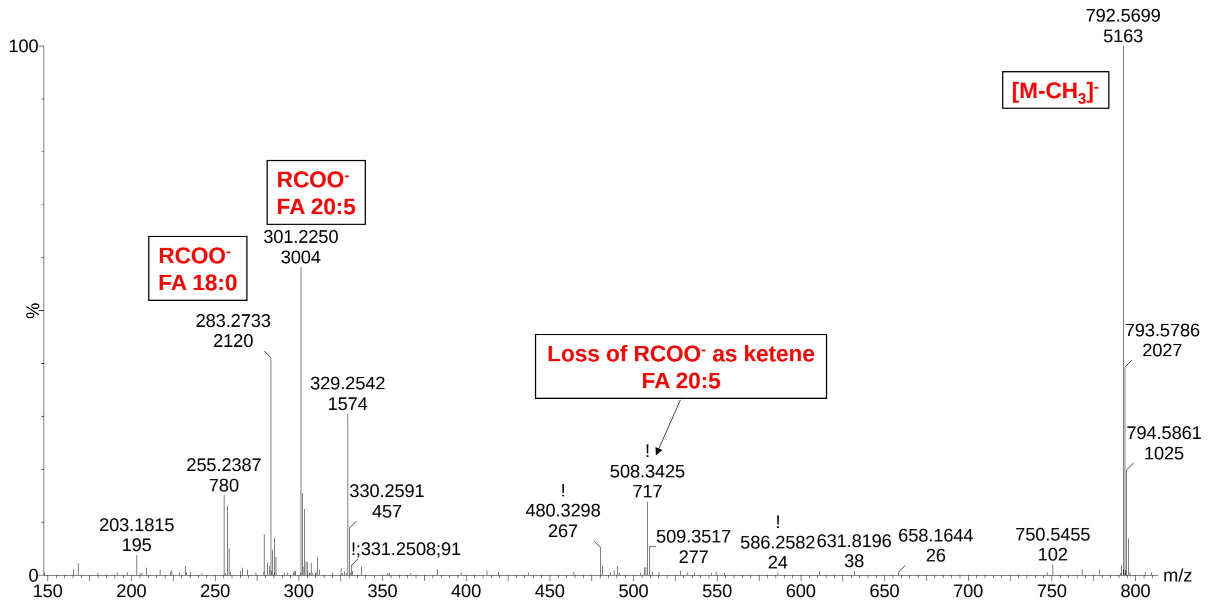

PC 38:6; PC 16:0/22:6; ESI-, fast DDA

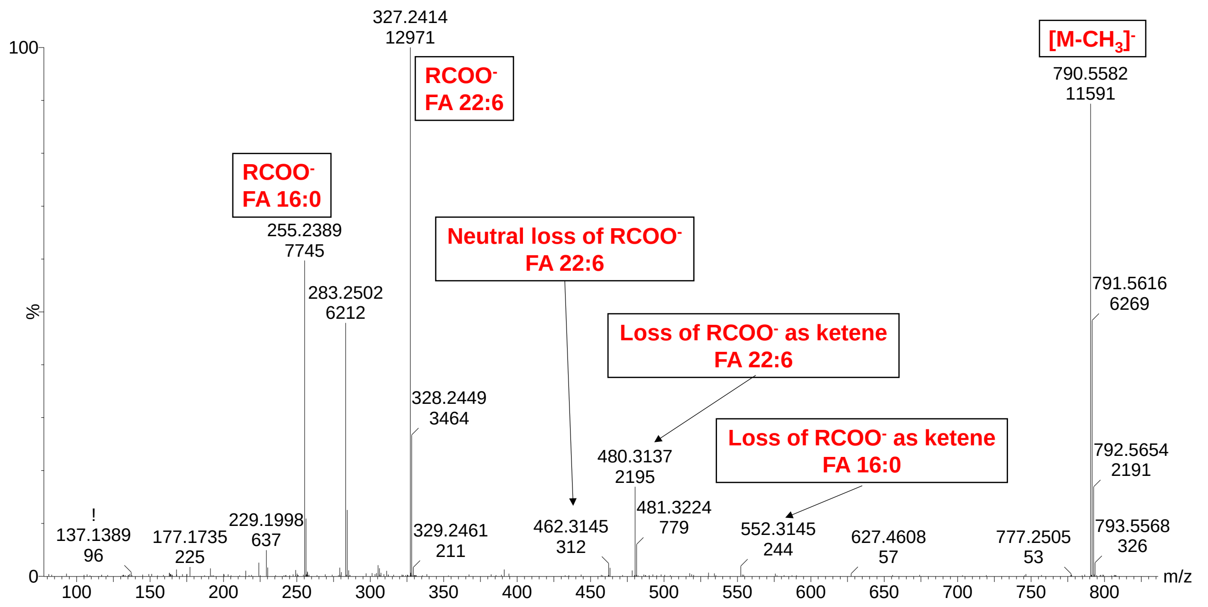

PC 38:6; PC 18:2/20:4; ESI-, MS^E

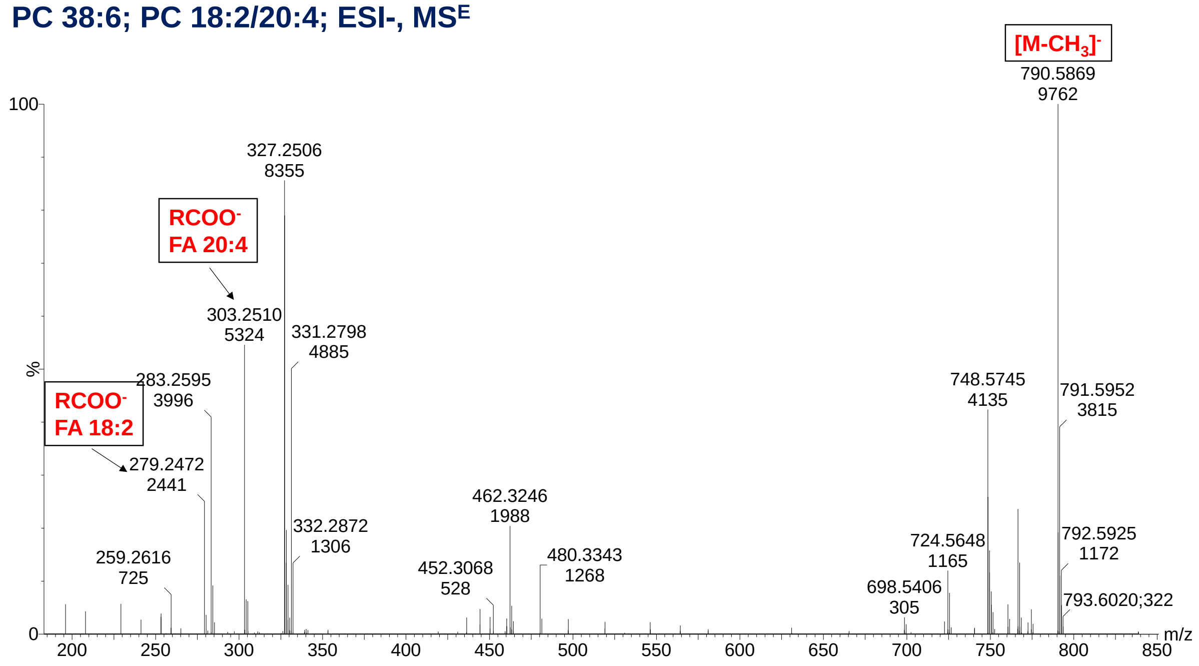

DG 32:1; DG 16:0_16:1; ESI+, fast DDA

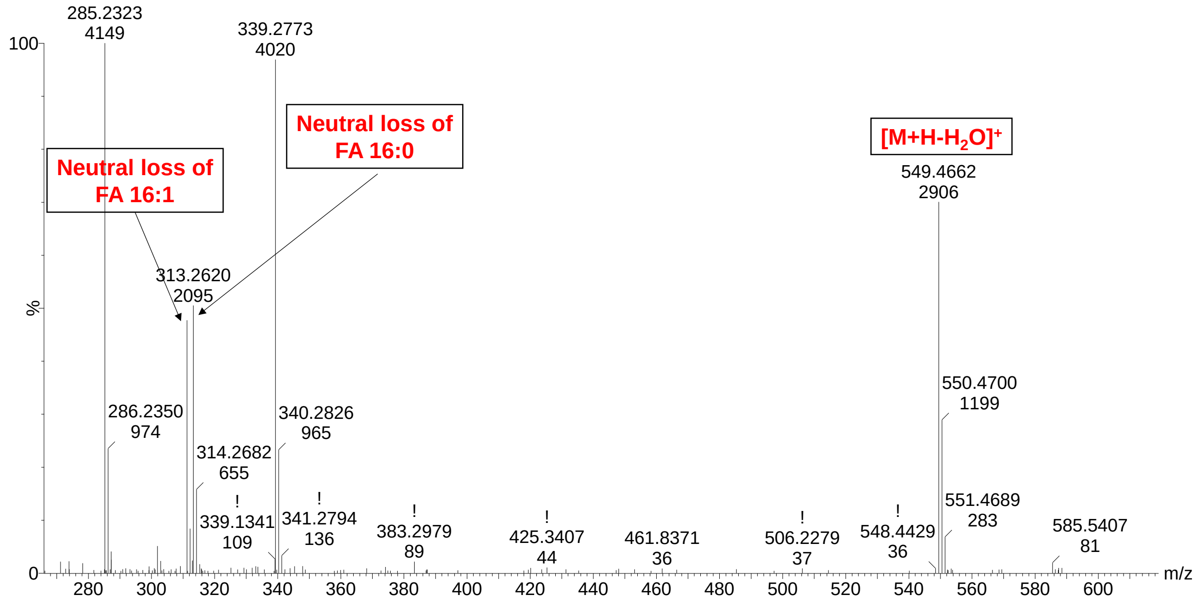

DG 32:1; DG 18:1_14:0; ESI+, fast DDA

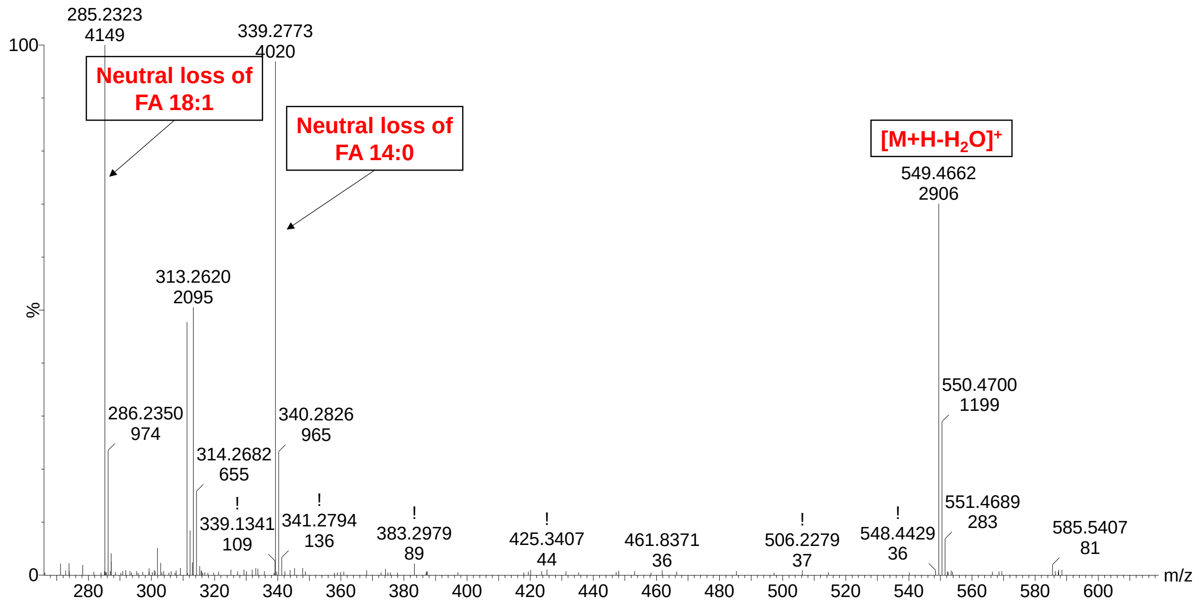

DG 34:2; DG 16:1_18:1; ESI+, fast DDA

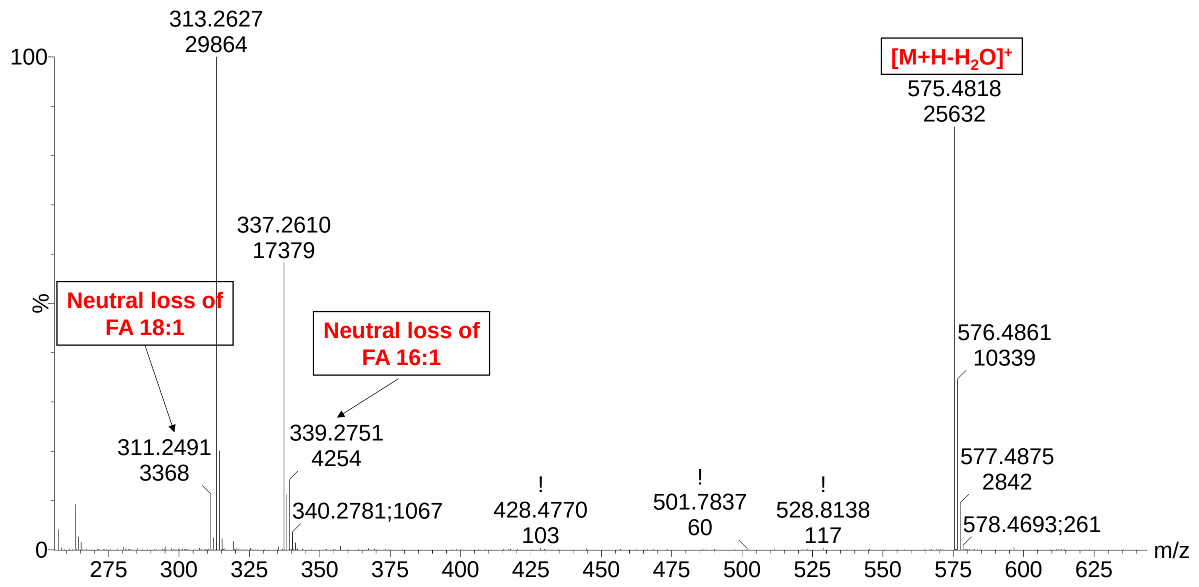

DG 34:2; DG 16:0_18:2; ESI+, fast DDA

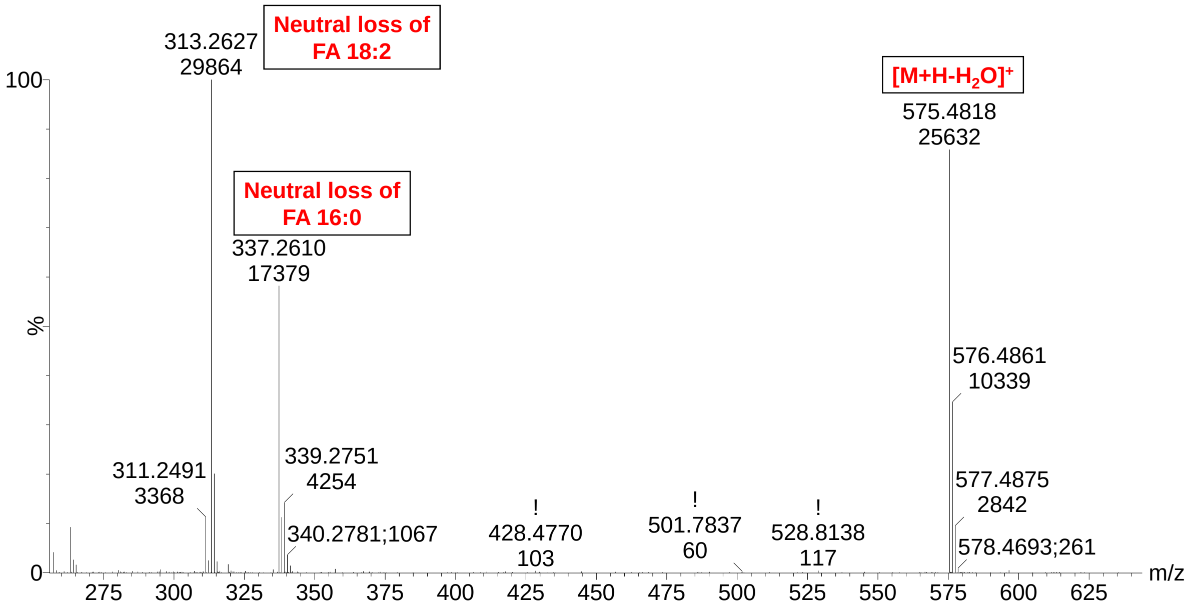

DG 36:4; DG 18:1_18:3; ESI+, fast DDA

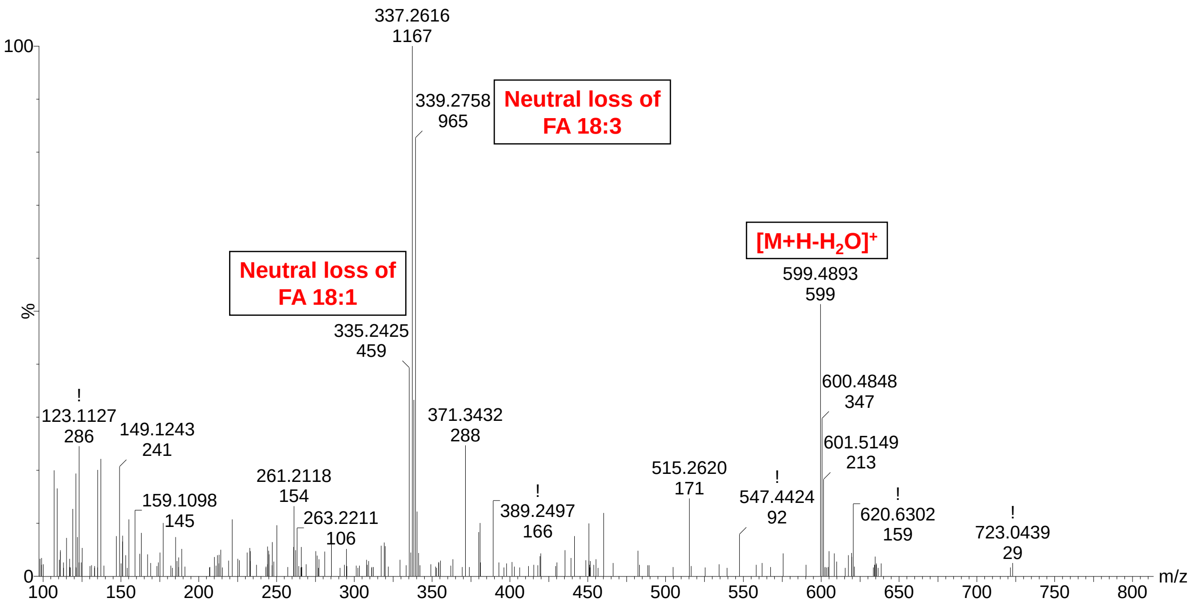

DG 34:2; DG 18:2_18:2; ESI+, fast DDA

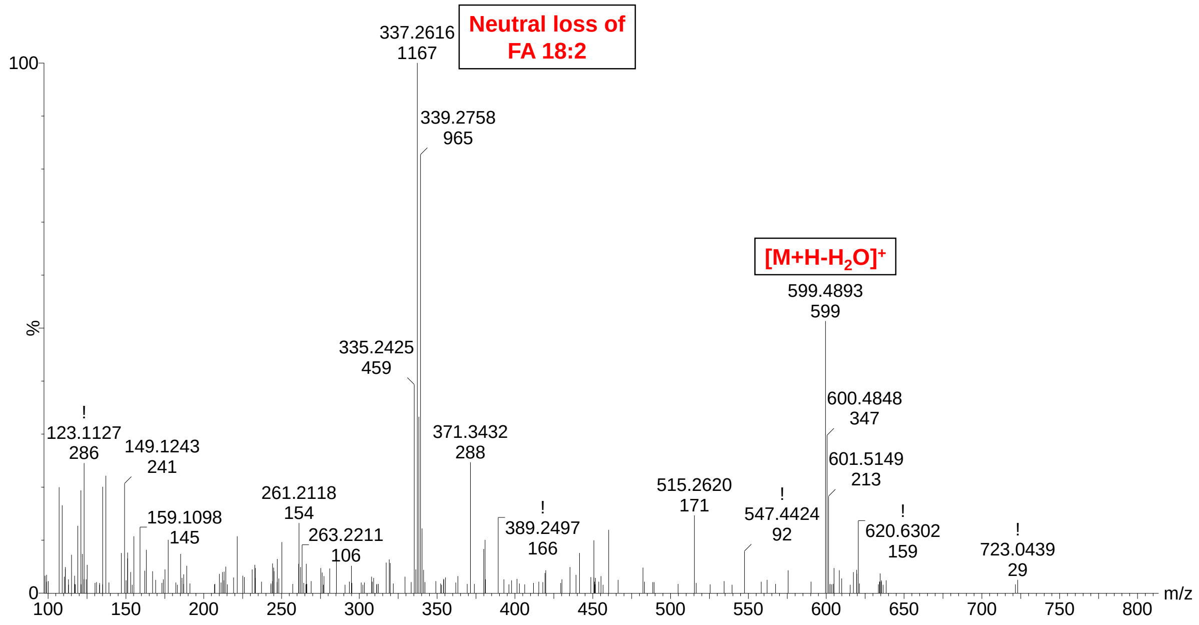

Cer 40:1;O2; Cer 17:1;O2/23:0; ESI+, MS^E

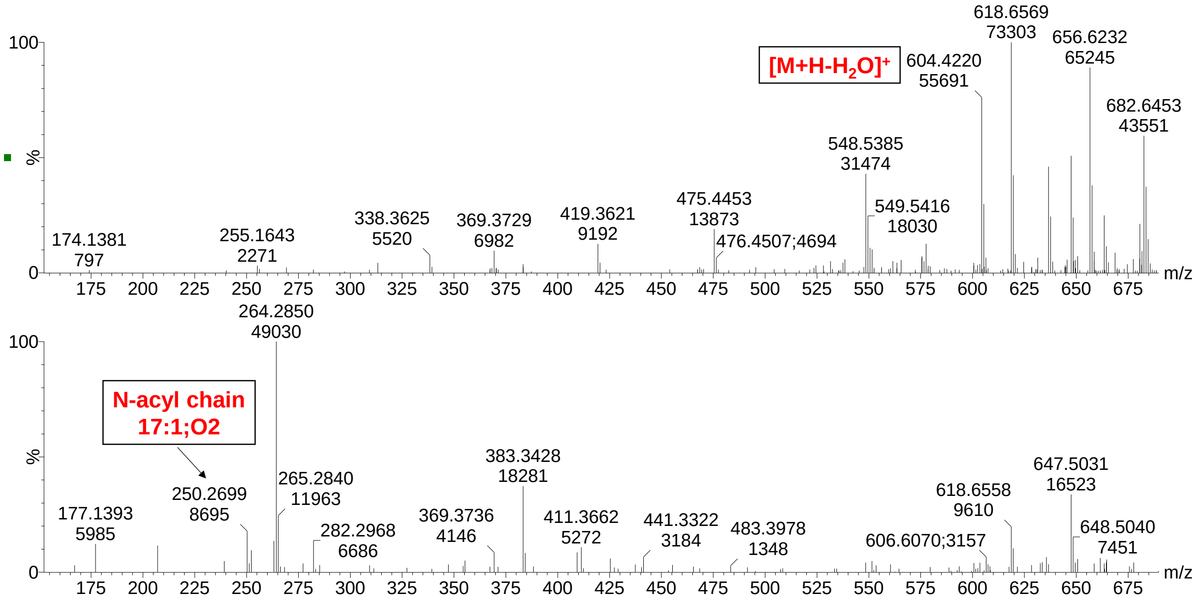

Cer 40:1;O2; Cer 18:1;O2/22:0; ESI+, MS^E

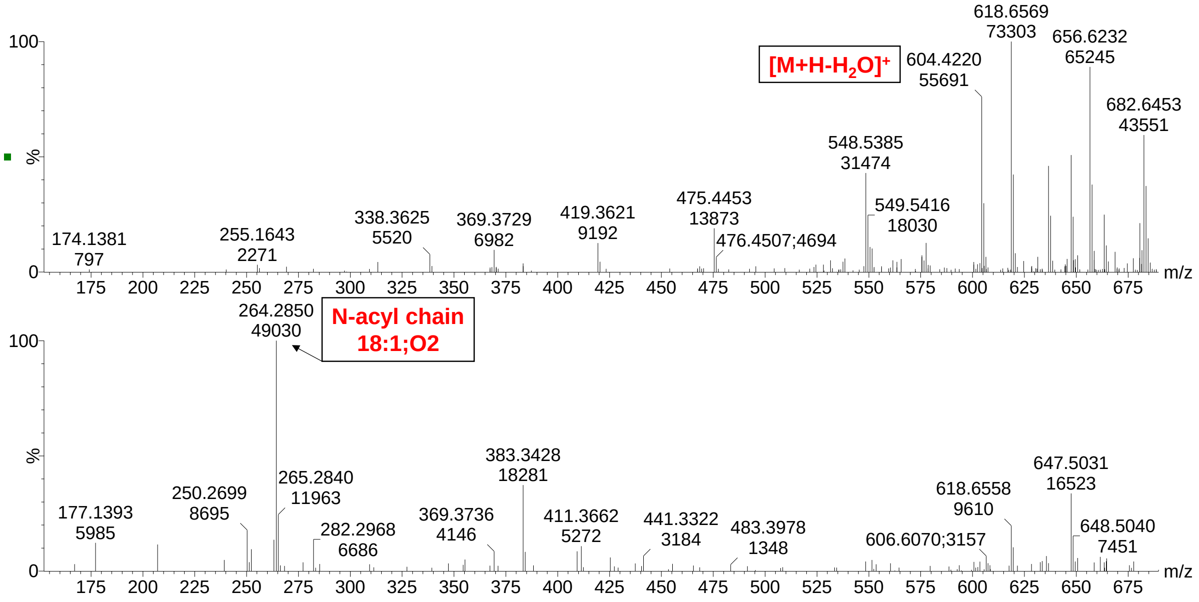

Cer 41:1;O2; Cer 17:1;O2/24:0; ESI+, fast DDA

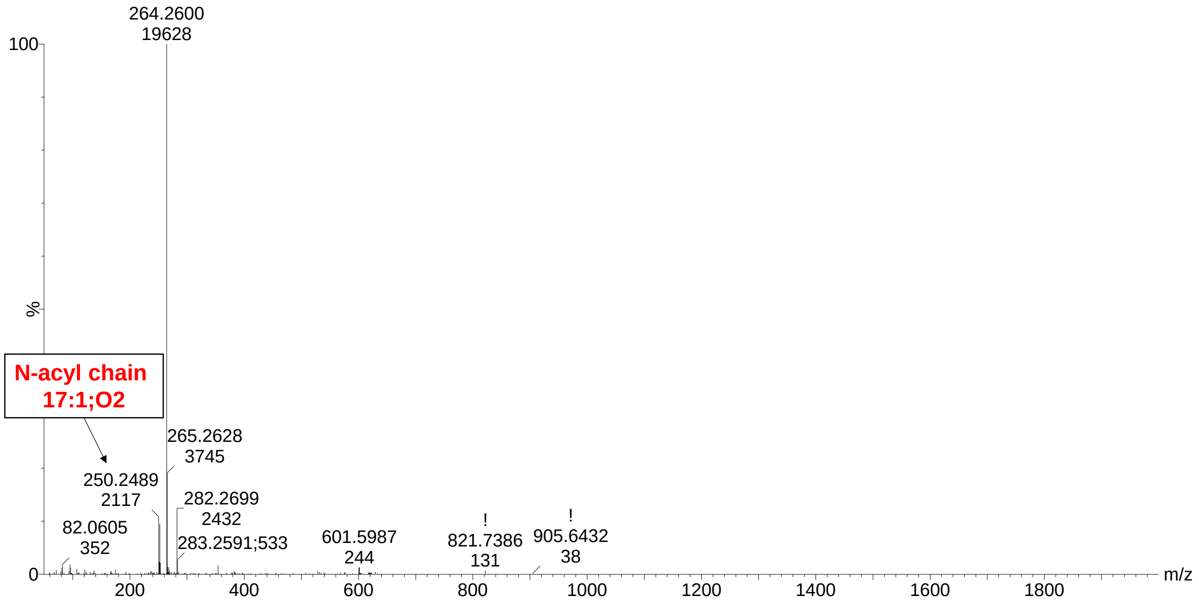

Cer 41:1;O2; Cer 18:1;O2/23:0; ESI+, fast DDA

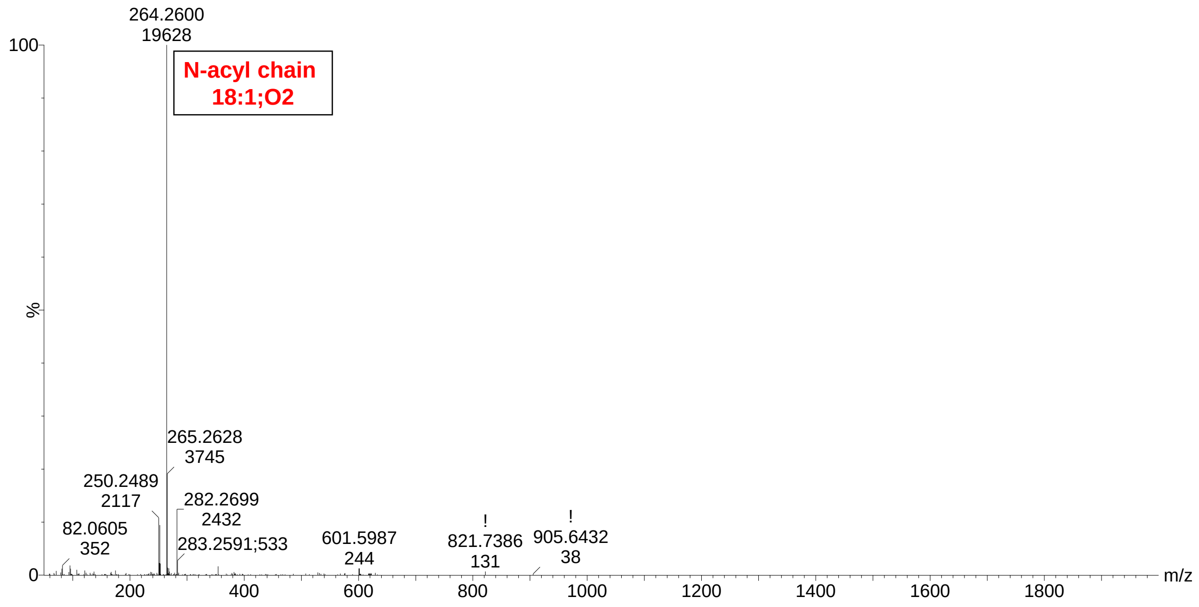

Cer 41:2;O2; Cer 17:1;O2/24:1; ESI+, fast DDA

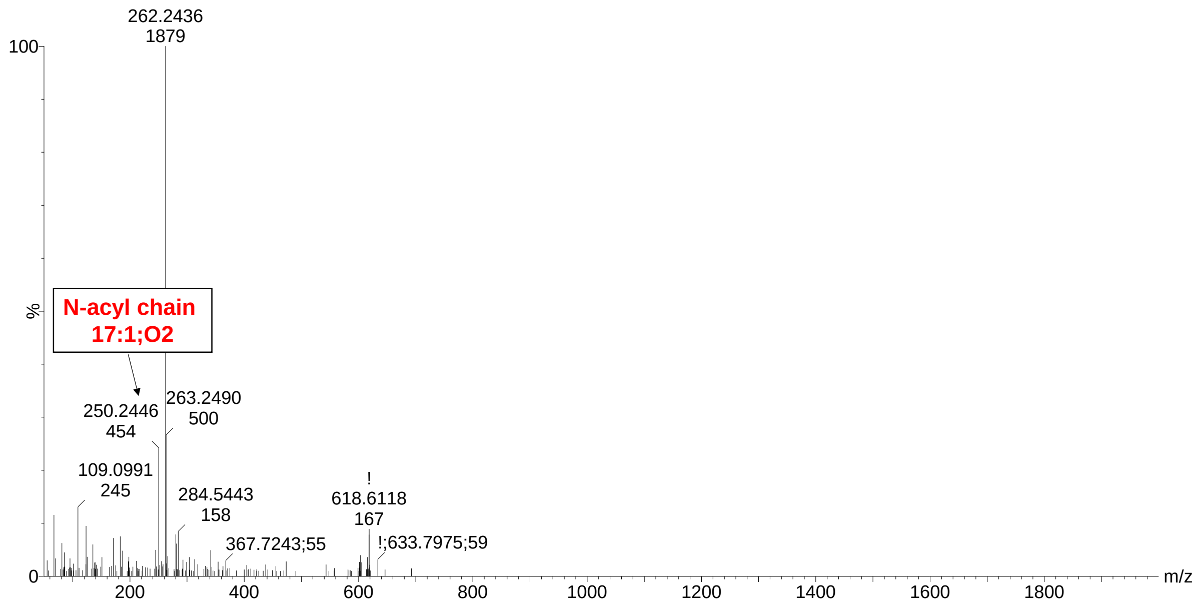

Cer 41:2;O2; Cer 18:2;O2/23:0; ESI+, fast DDA

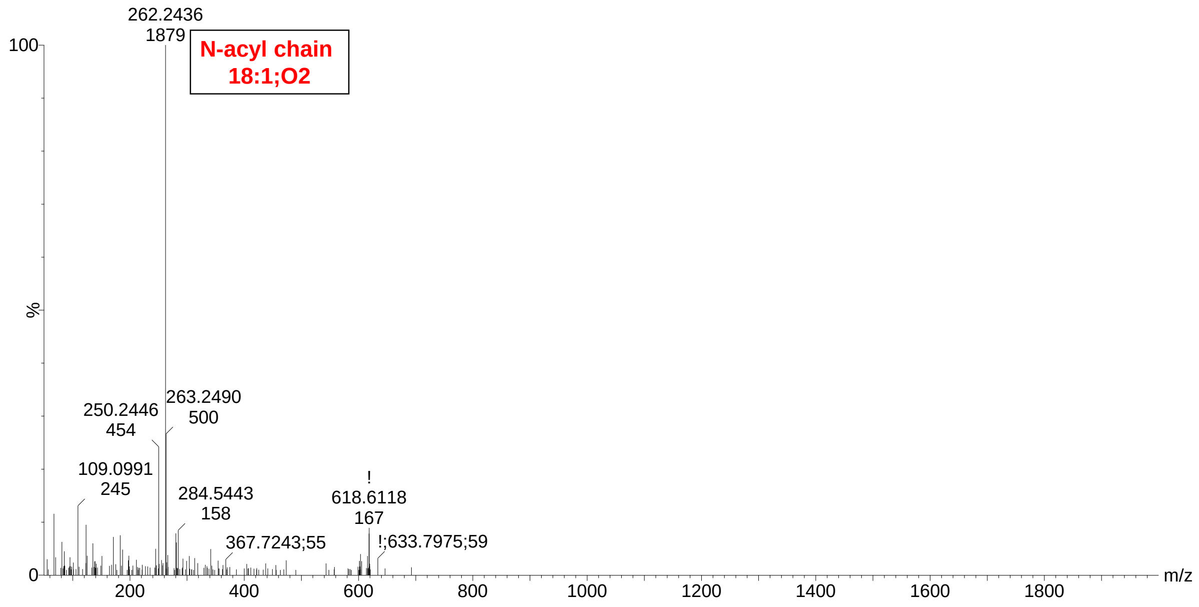

Cer 42:1;O2; Cer 18:1;O2/24:0; ESI+, fast DDA

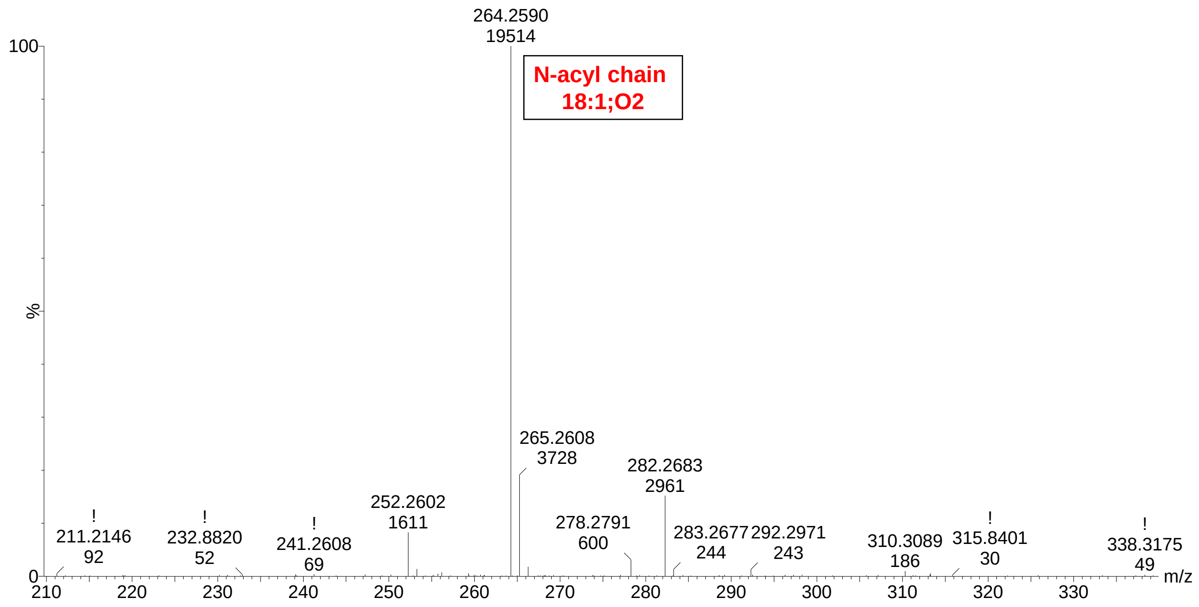

Cer 42:1;O2; Cer 20:1;O2/22:0; ESI+, fast DDA

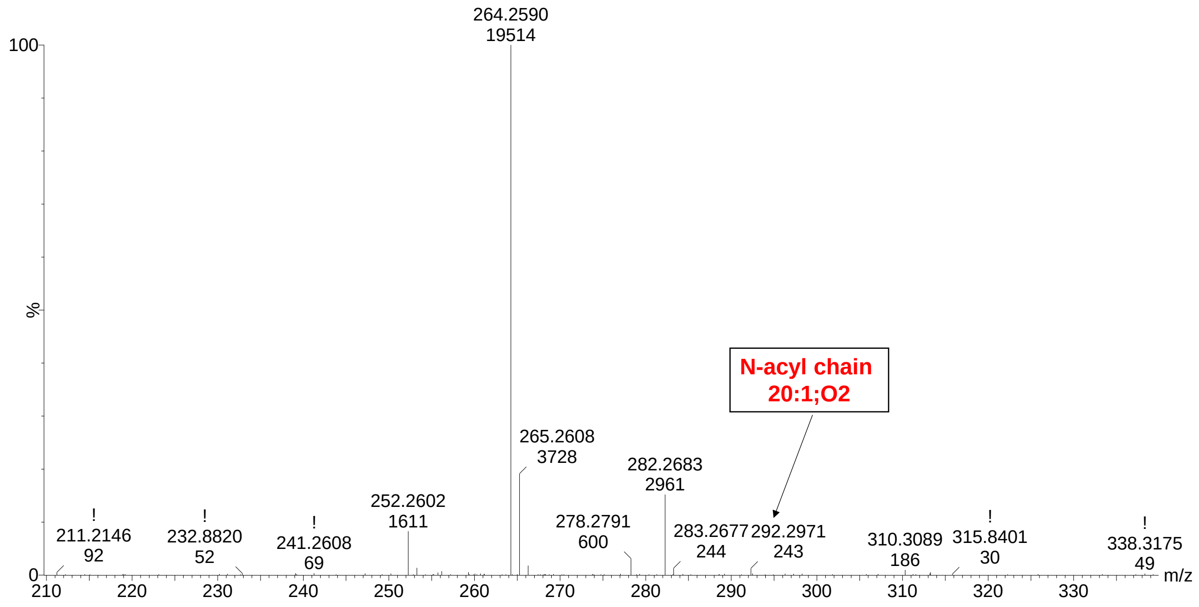

Cer 42:2;O2; Cer 18:2;O2/24:0; ESI+, MS^E

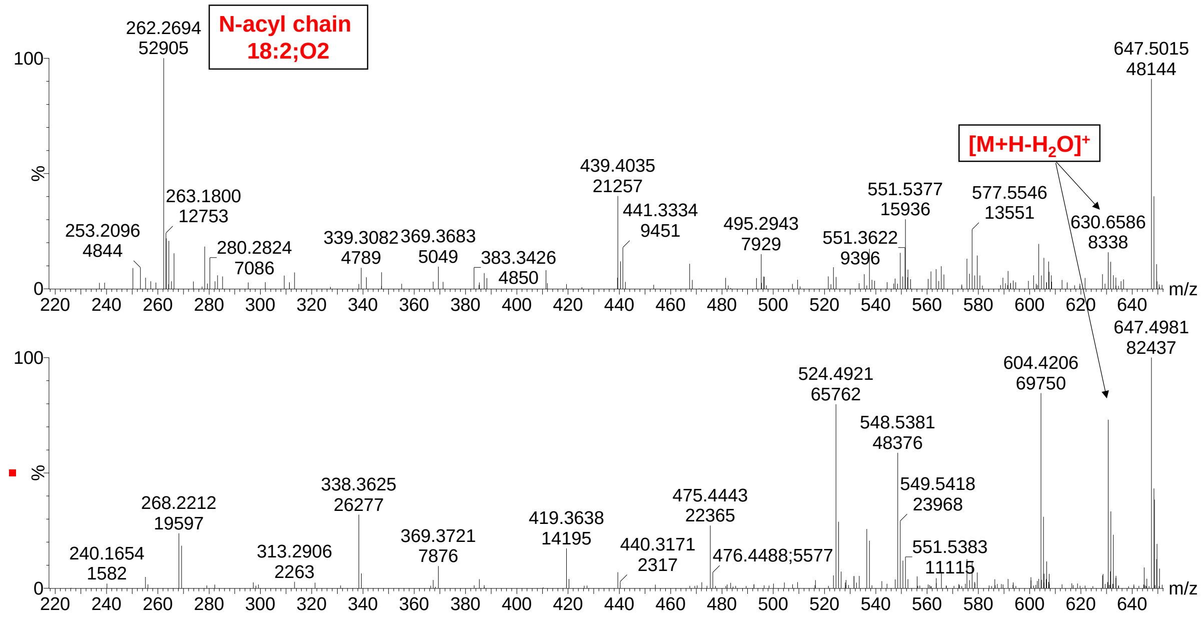

Cer 42:2;O2; Cer 19:1;O2/23:1; ESI+, MS^E

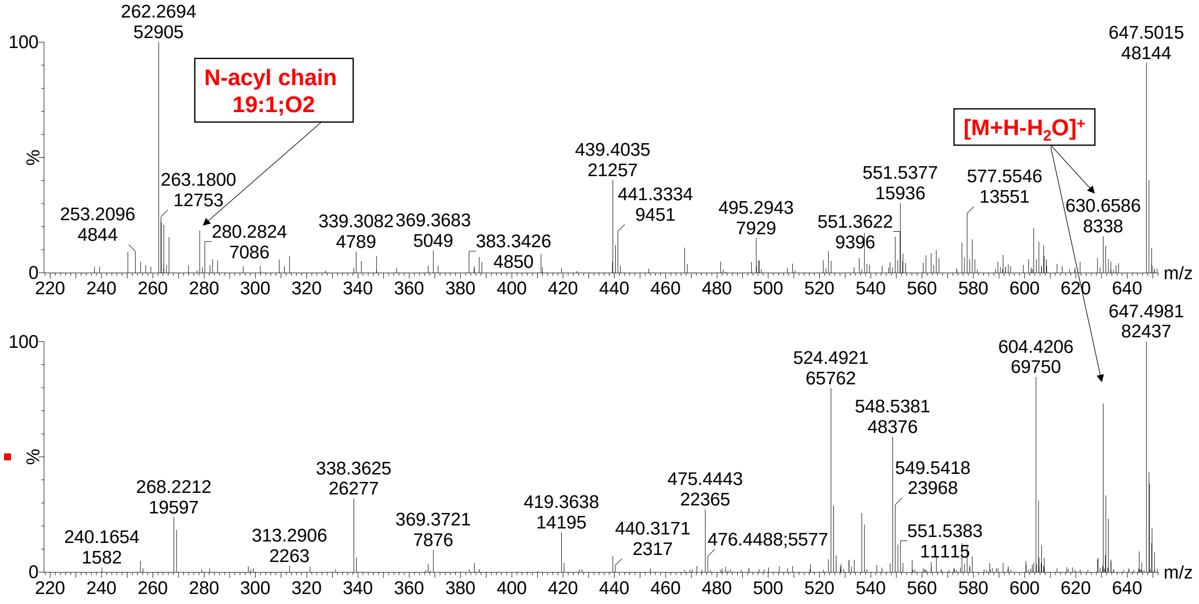

Cer 43:1;O2; Cer 18:1;O2/25:0; ESI+, MS^E

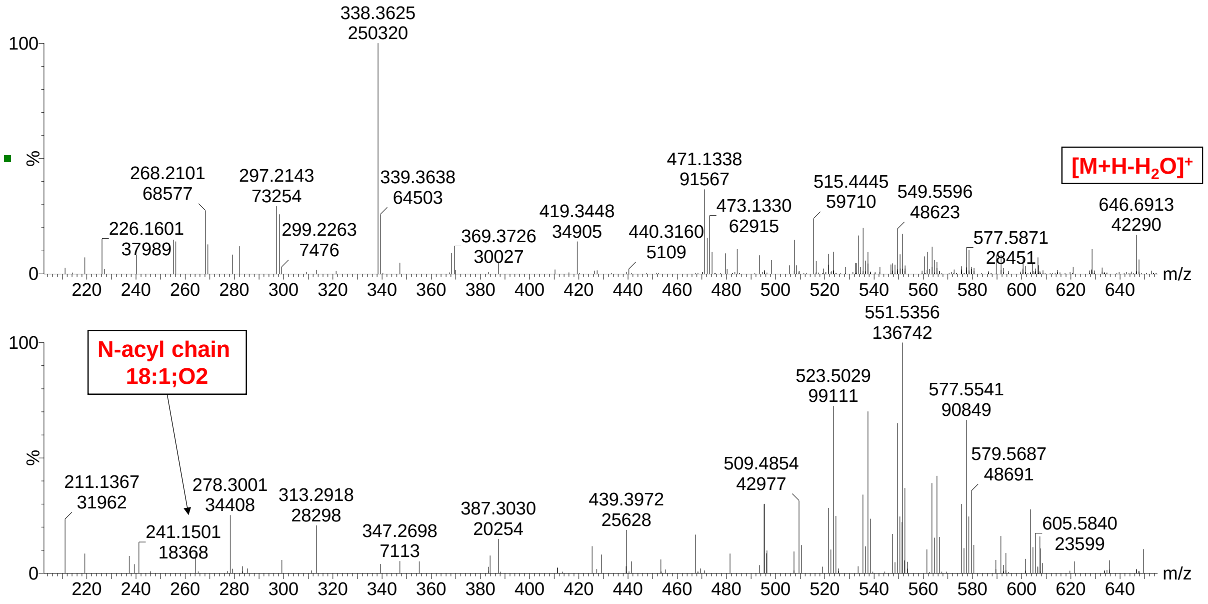

Cer 43:1;O2; Cer 19:1;O2/24:0; ESI+, MS^E

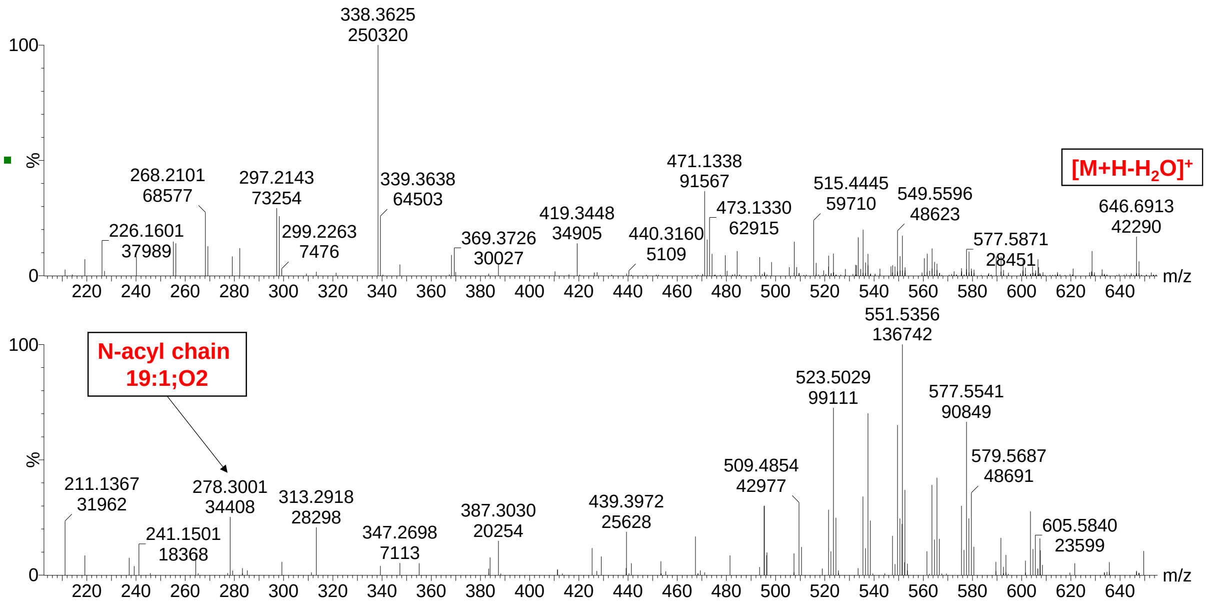

TG 44:1;O2; TG 14:0_18:1_12:0; ESI+, fast DDA

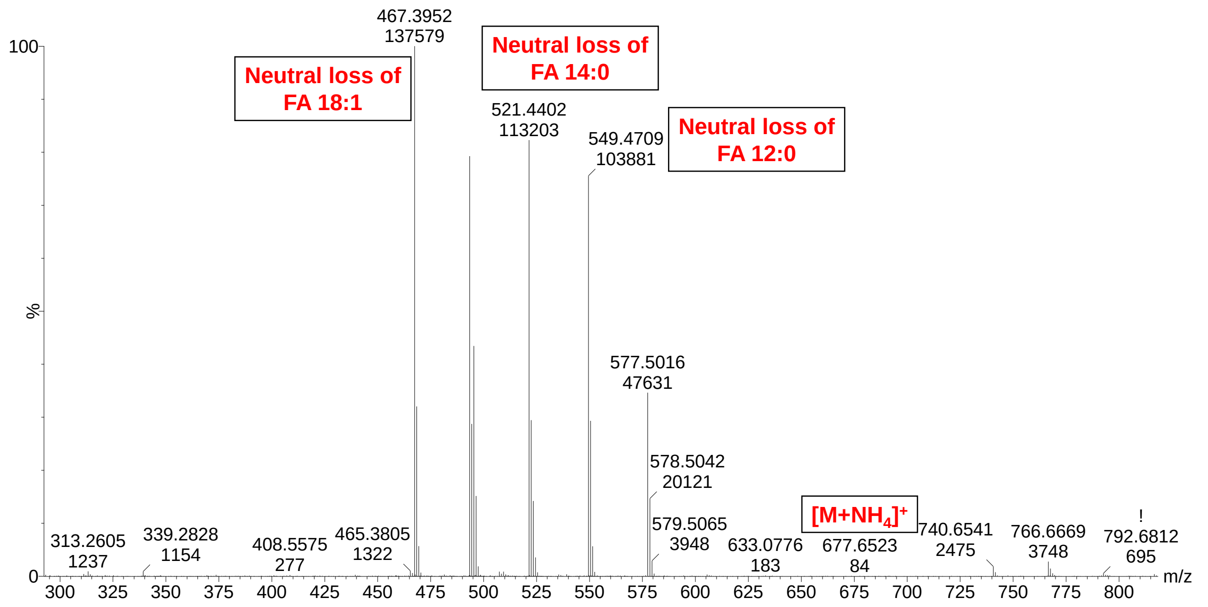

TG 44:1;O2; TG 12:0_16:0_16:1; ESI+, fast DDA

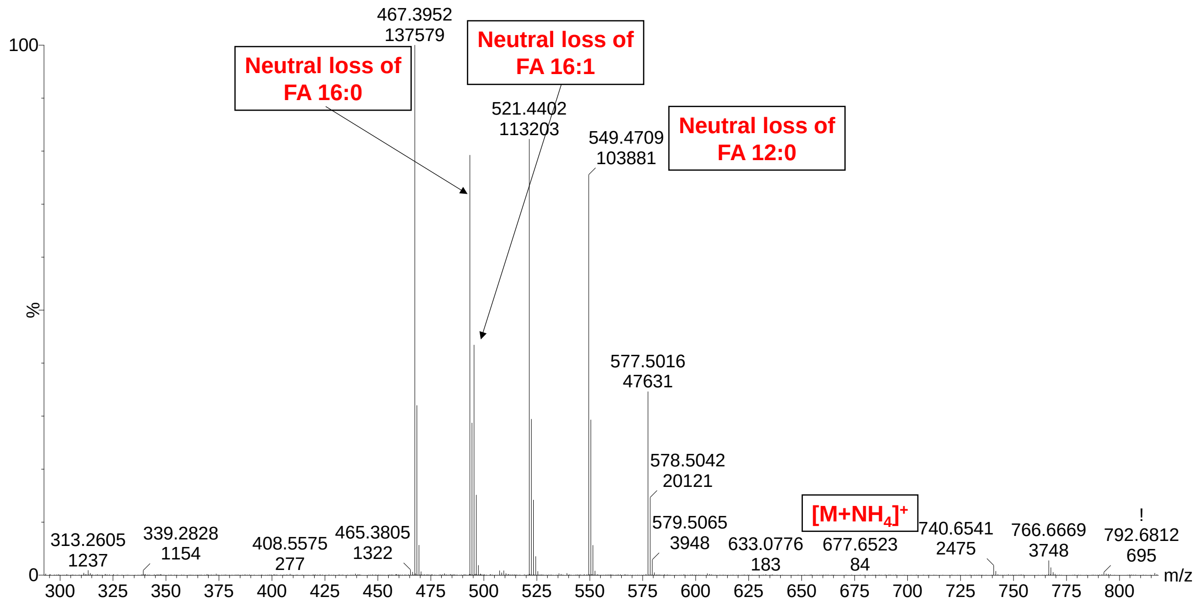

TG 44:1;O2; TG 16:1_14:0_14:0; ESI+, fast DDA

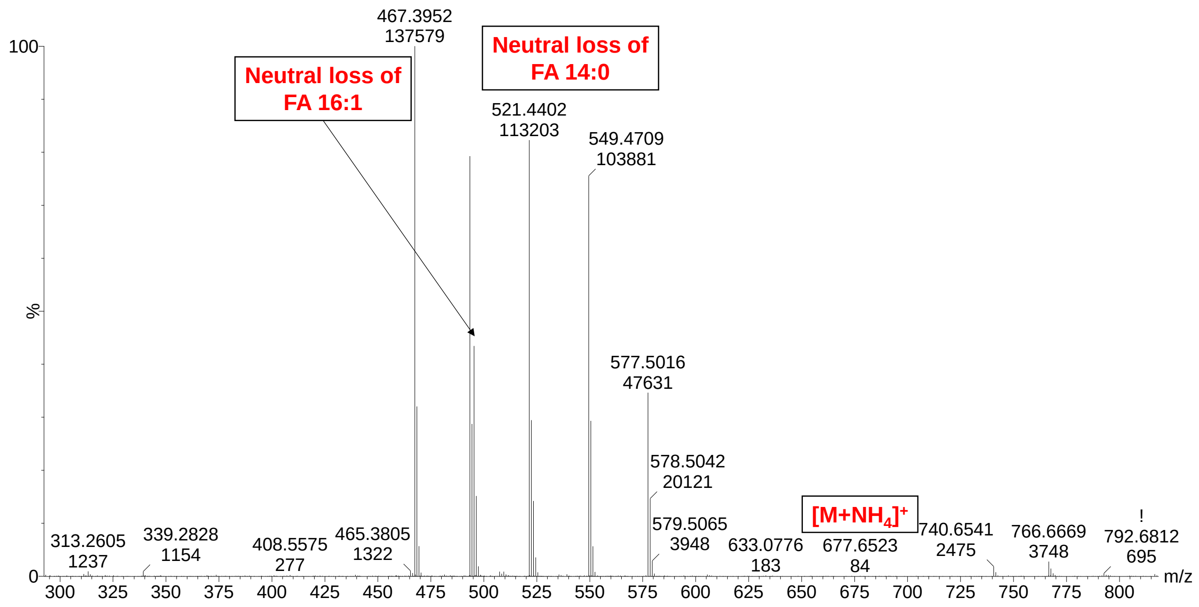

TG 46:1;O2; TG 16:0_18:1_12:0; ESI+, fast DDA

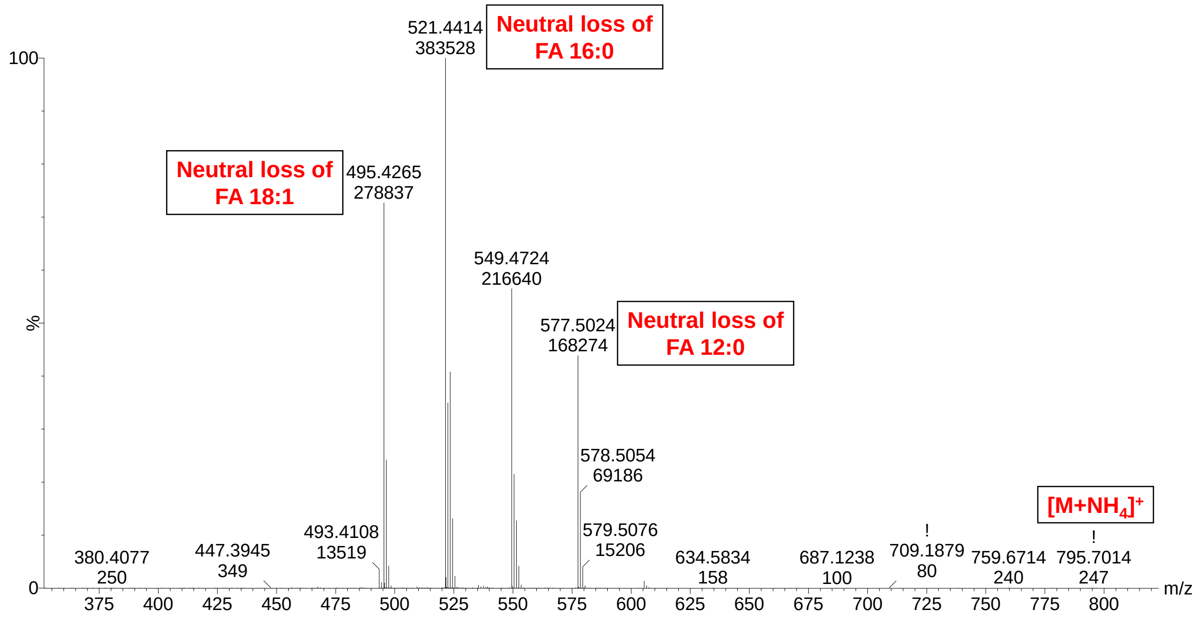

TG 46:1;O2; TG 16:0_16:1_14:0; ESI+, fast DDA

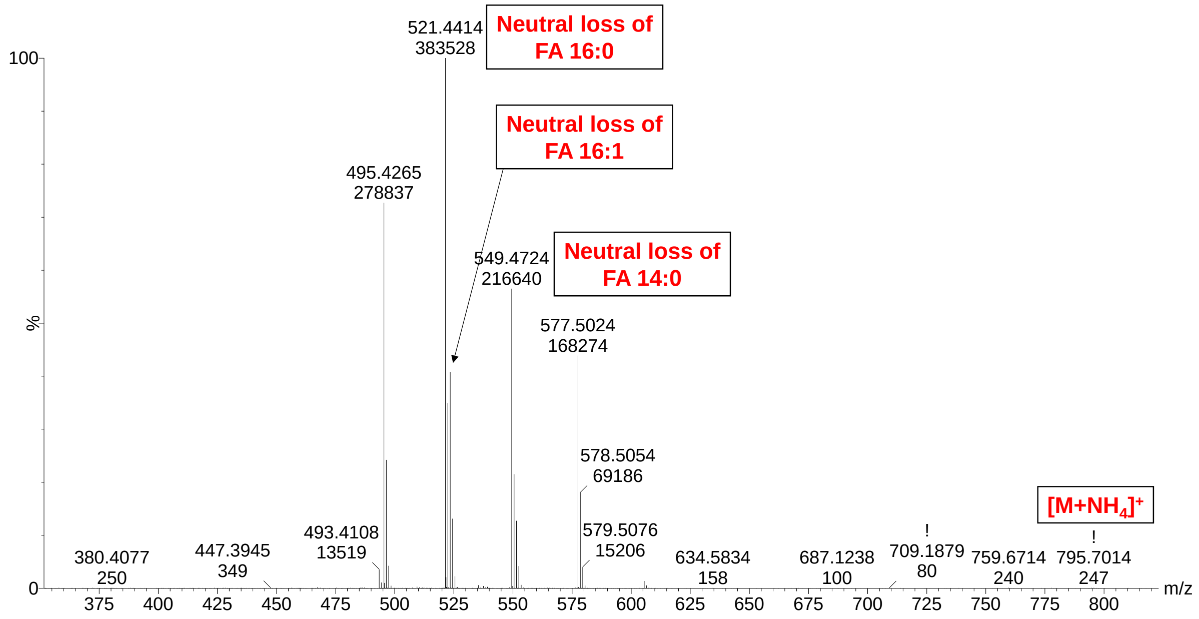

TG 46:3;O2; TG 12:0_16:1_18:2; ESI+, fast DDA

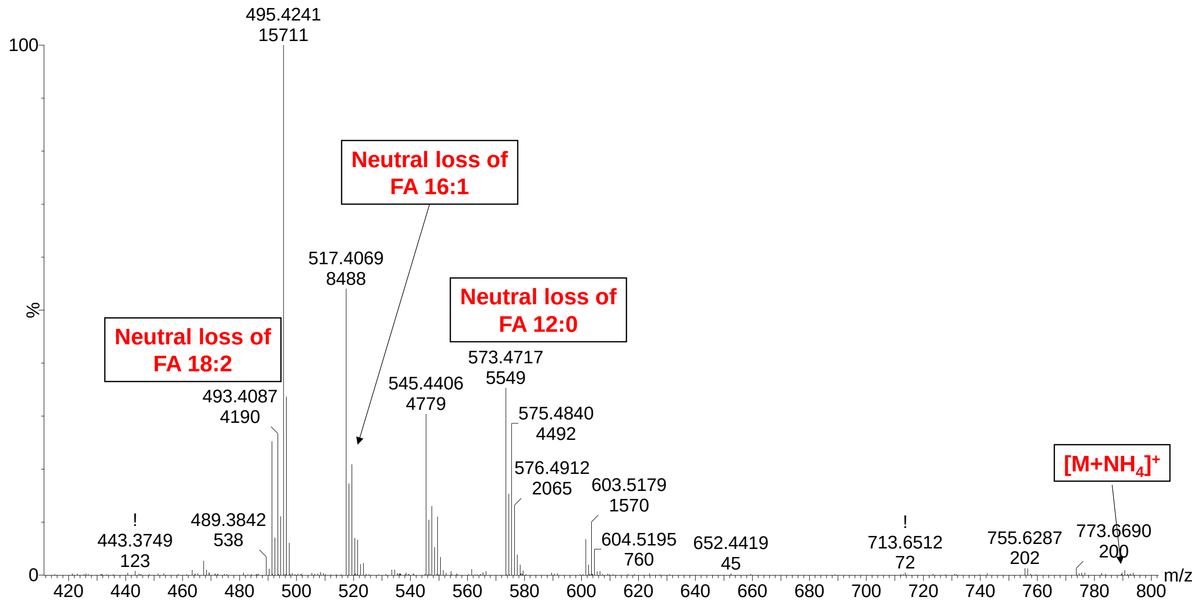

TG 46:3;O2; TG 14:0_14:1_18:2; ESI+, fast DDA

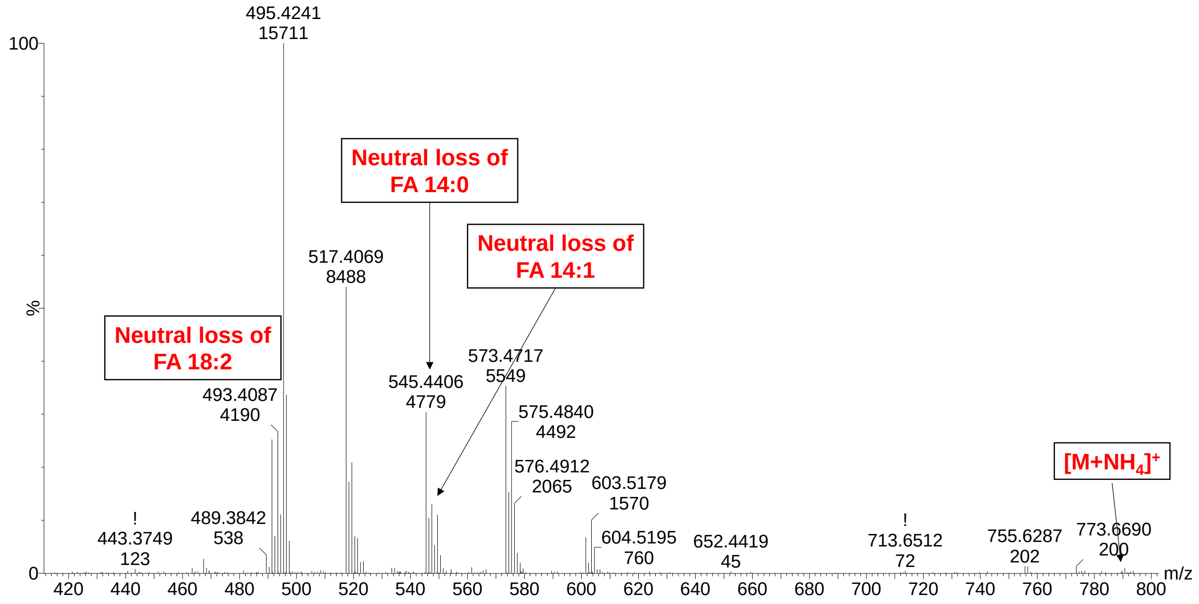

TG 48:0;O2; TG 16:0_16:0_16:0; ESI+, fast DDA

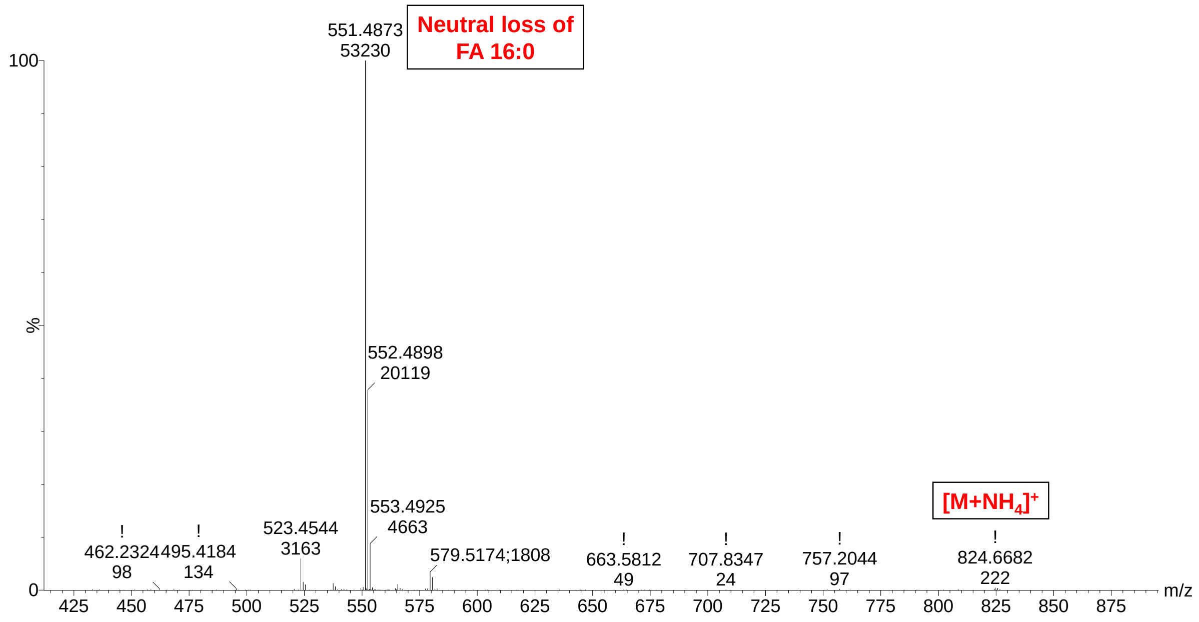

TG 48:0;O2; TG 14:0_16:0_18:0; ESI+, fast DDA

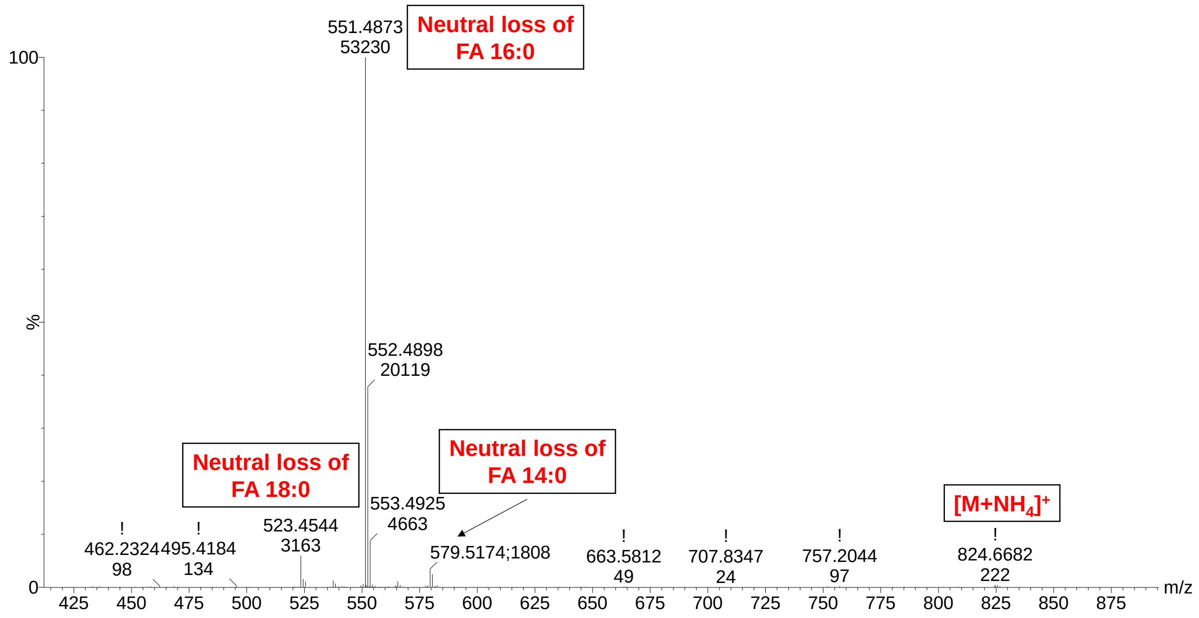

TG 48:1;O2; TG 16:0_16:1_16:0; ESI+, fast DDA

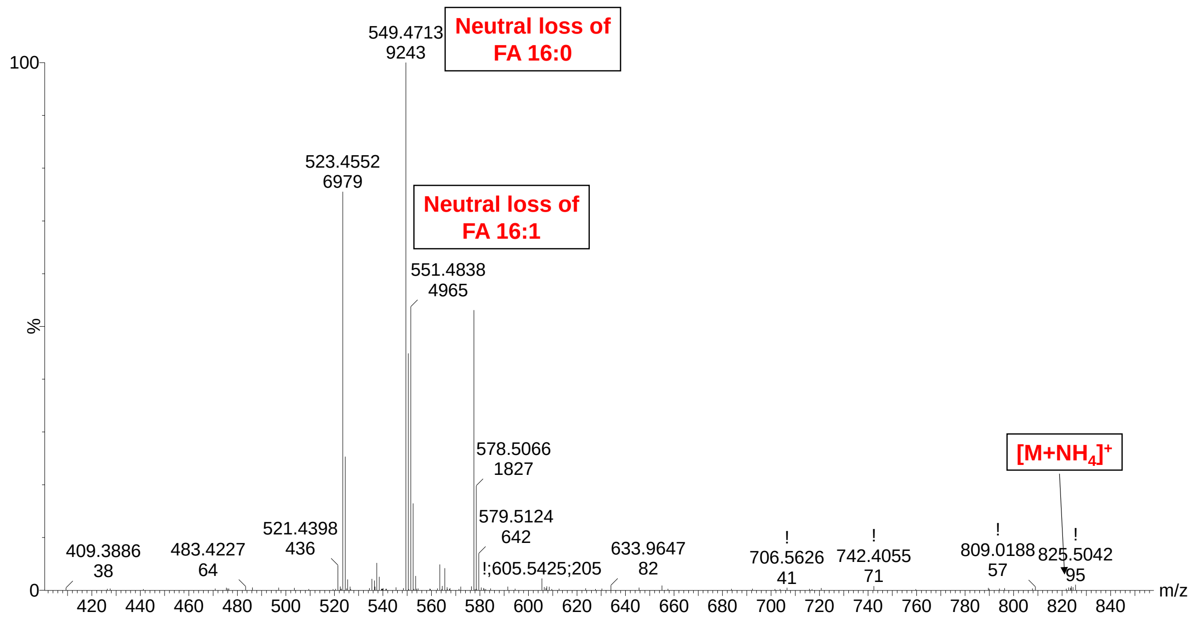

TG 48:1;O2; TG 16:0_18:1_14:0; ESI+, fast DDA

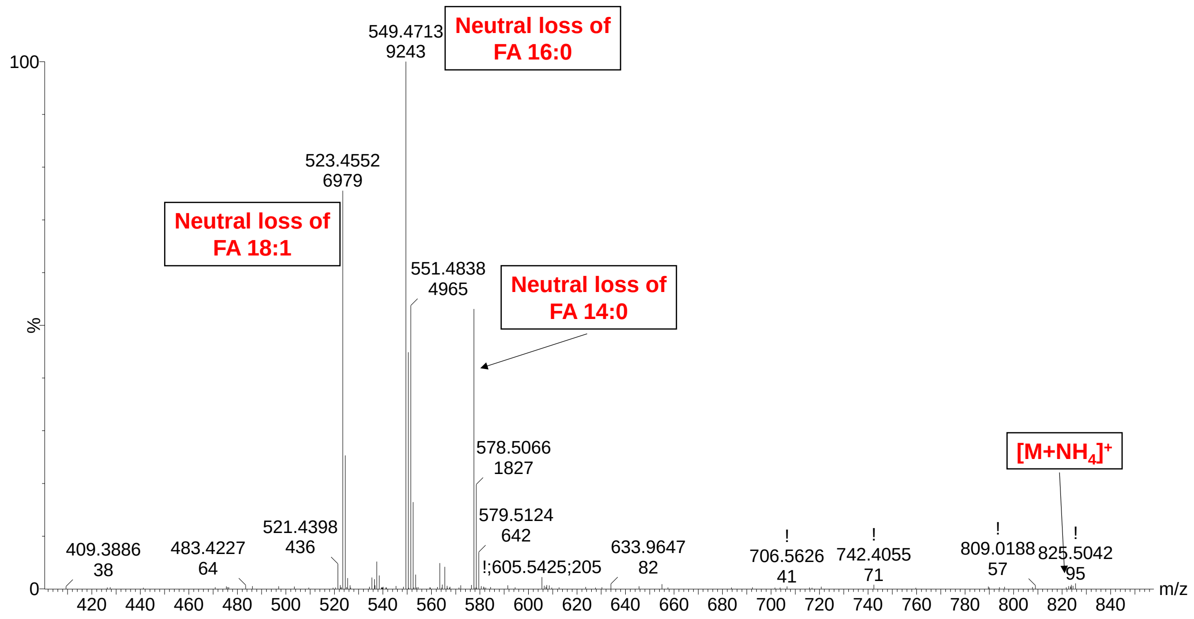

TG 48:1;O2; TG 18:0_18:1_12:0; ESI+, fast DDA

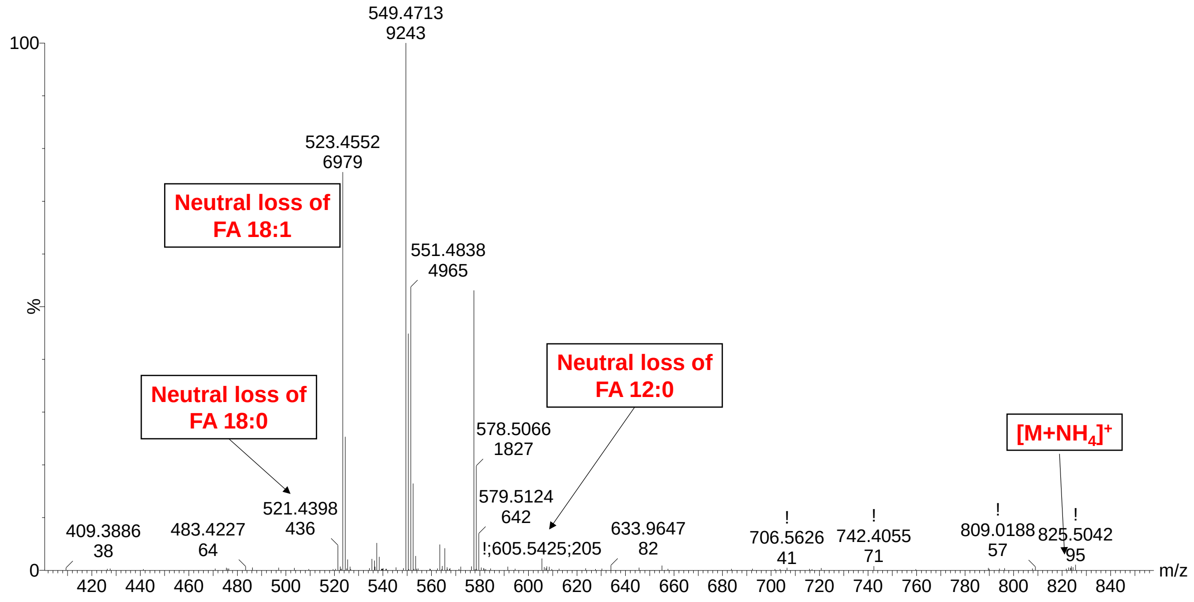

TG 48:2;O2; TG 18:1_18:1_12:0; ESI+, fast DDA

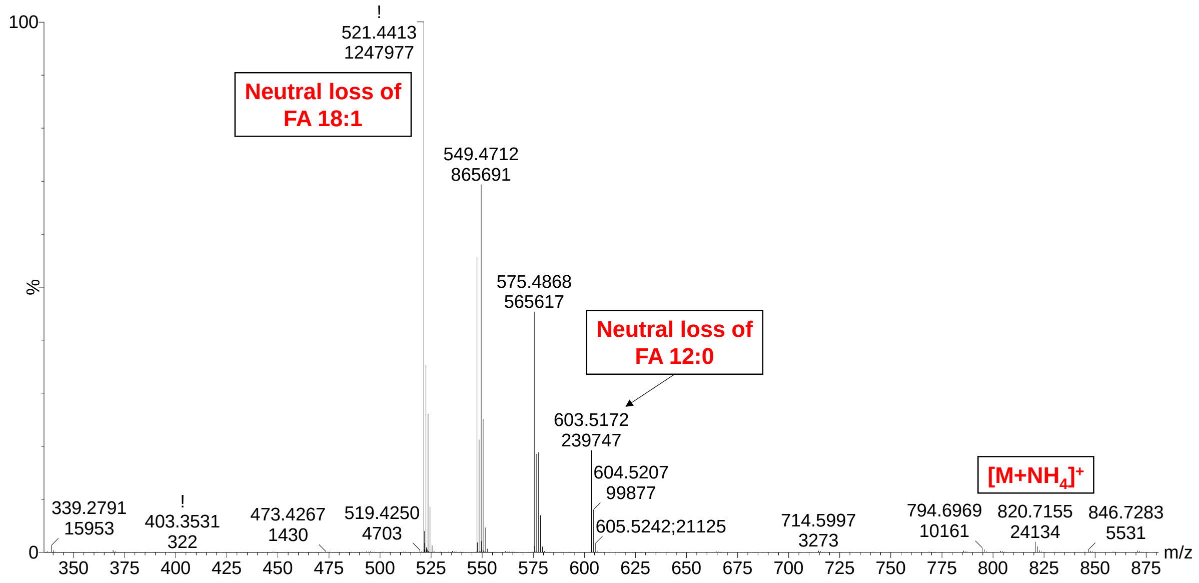

TG 48:2;O2; TG 16:0_18:2_14:0; ESI+, fast DDA

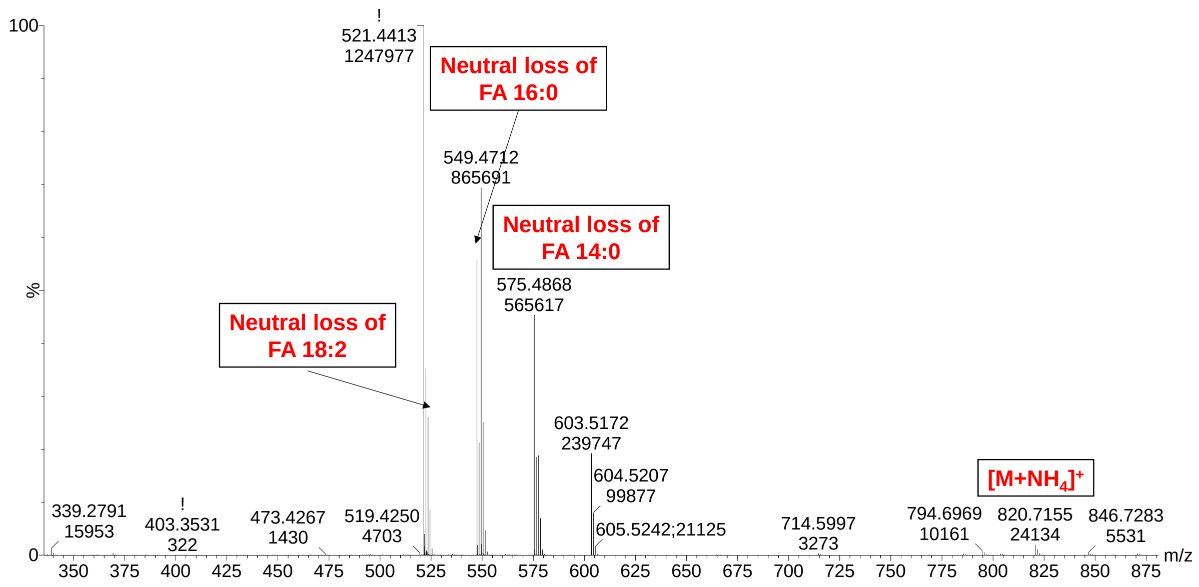

TG 48:2;O2; TG 18:1_16:1_14:0; ESI+, fast DDA

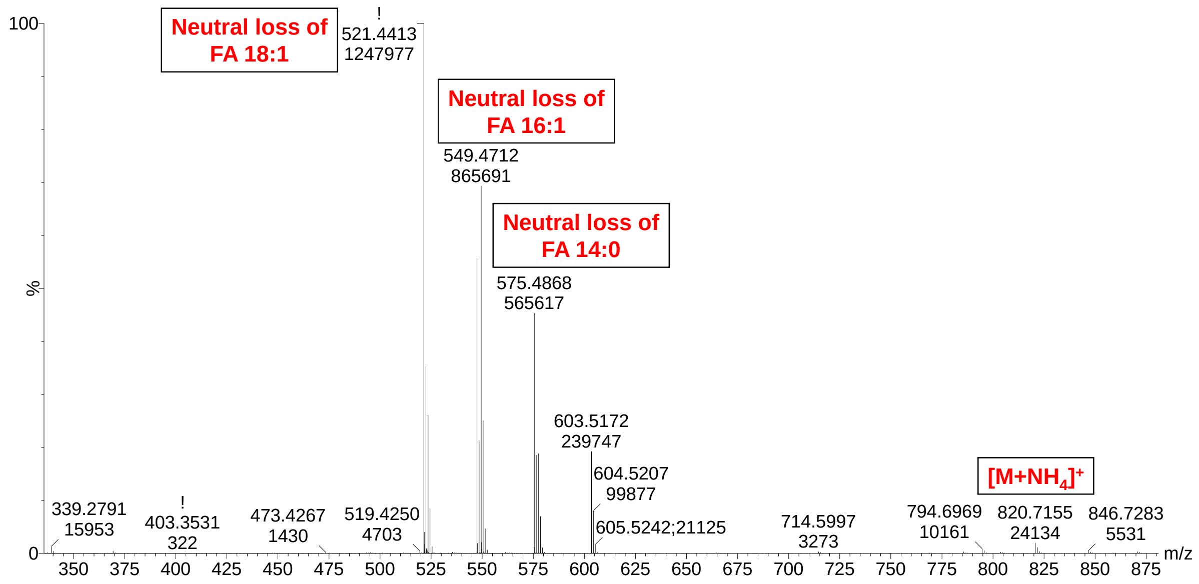

TG 50:1;O2; TG 16:0_18:1_16:0; ESI+, fast DDA

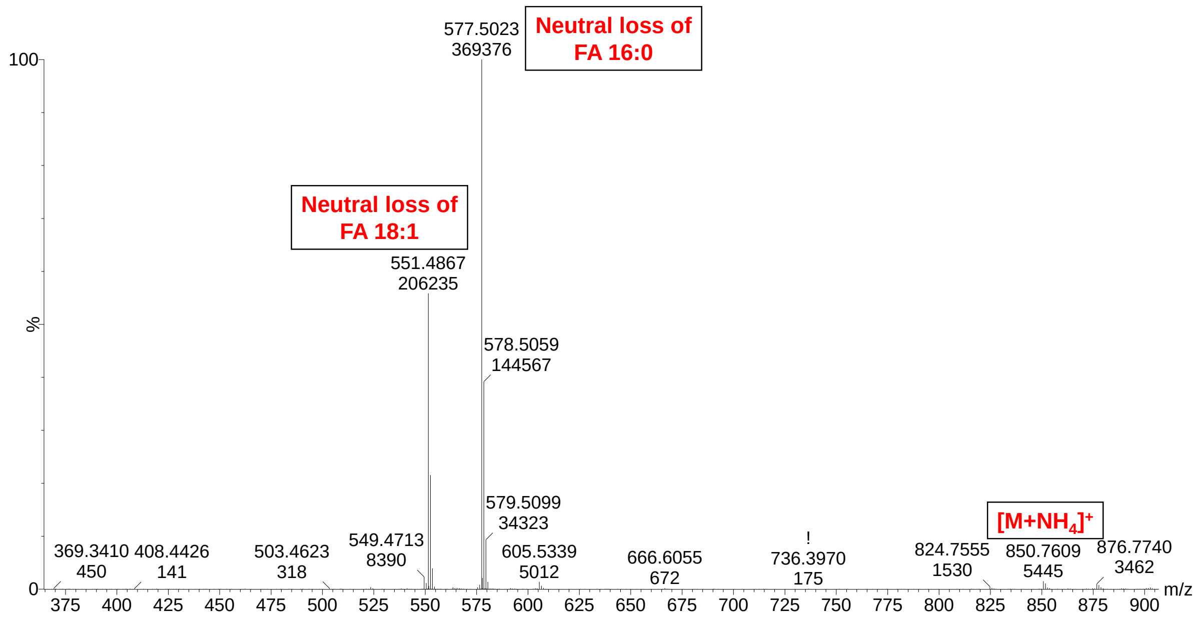

TG 50:1;O2; TG 18:0_18:1_14:0; ESI+, fast DDA

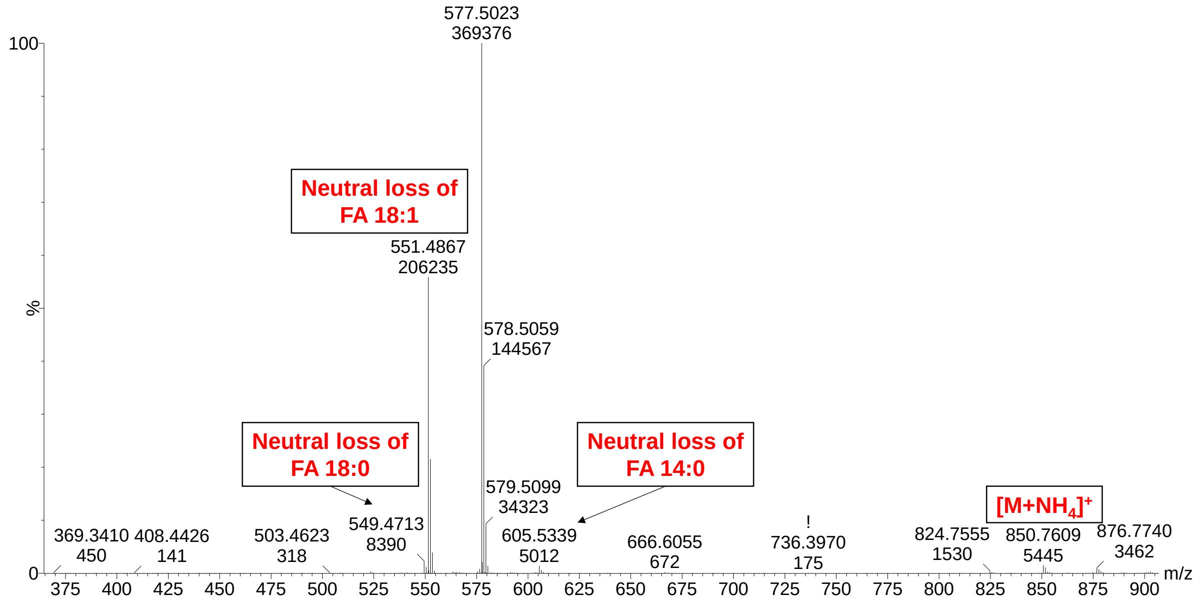

TG 50:2;O2; TG 18:1_18:1_14:0; ESI+, fast DDA

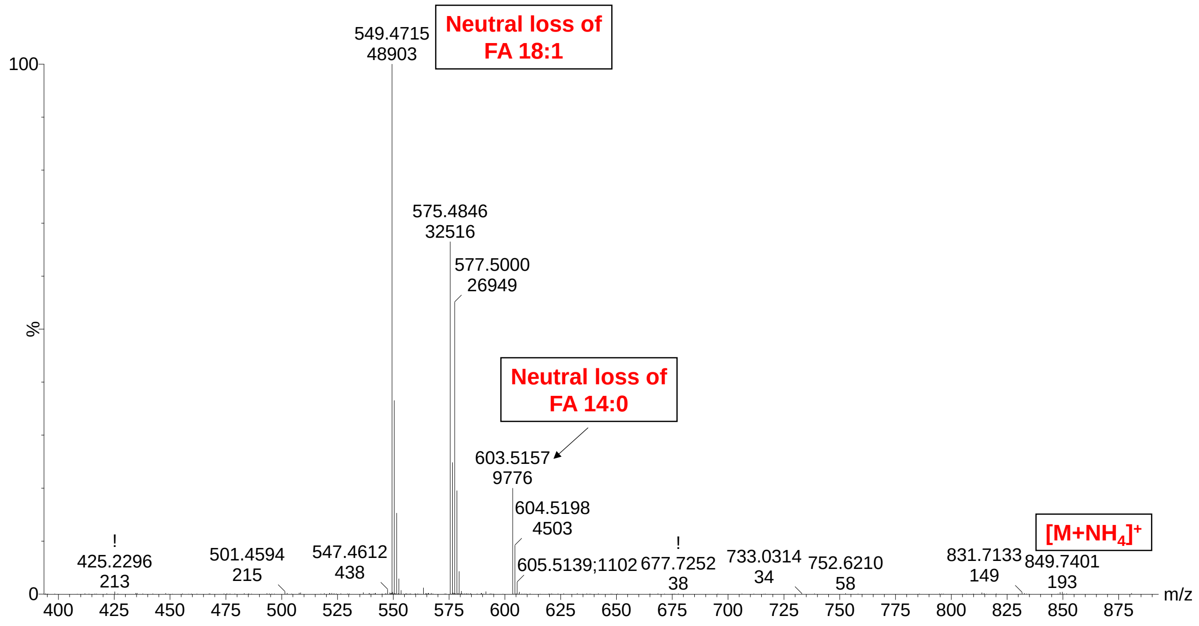

TG 50:2;O2; TG 16:0_16:0_18:2; ESI+, fast DDA

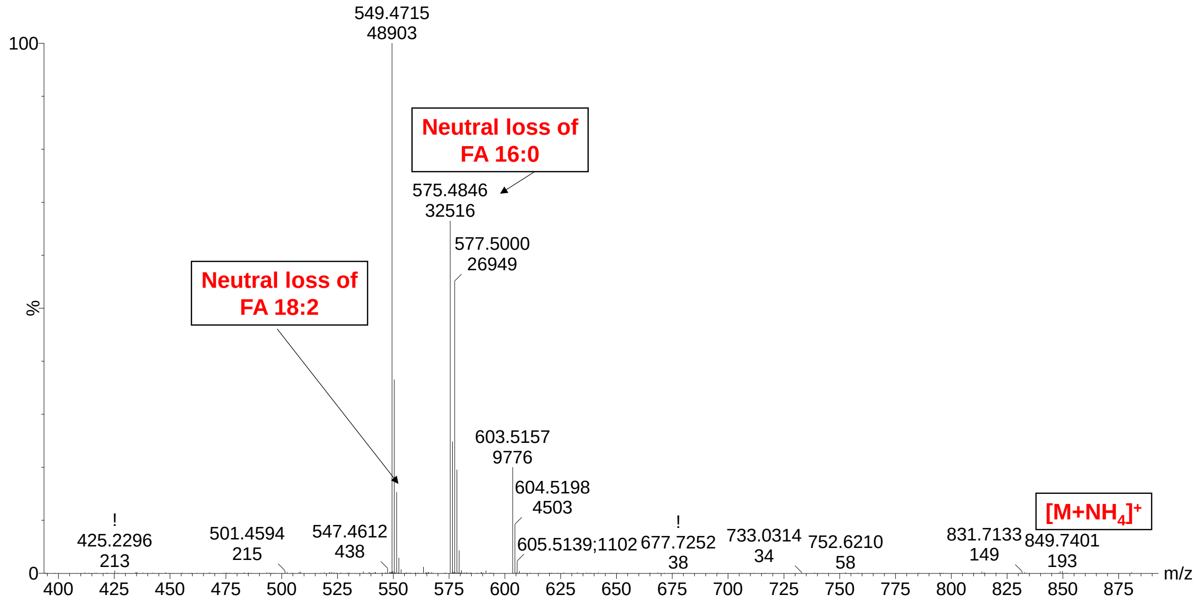

TG 50:2;O2; TG 16:0_18:1_16:1; ESI+, fast DDA

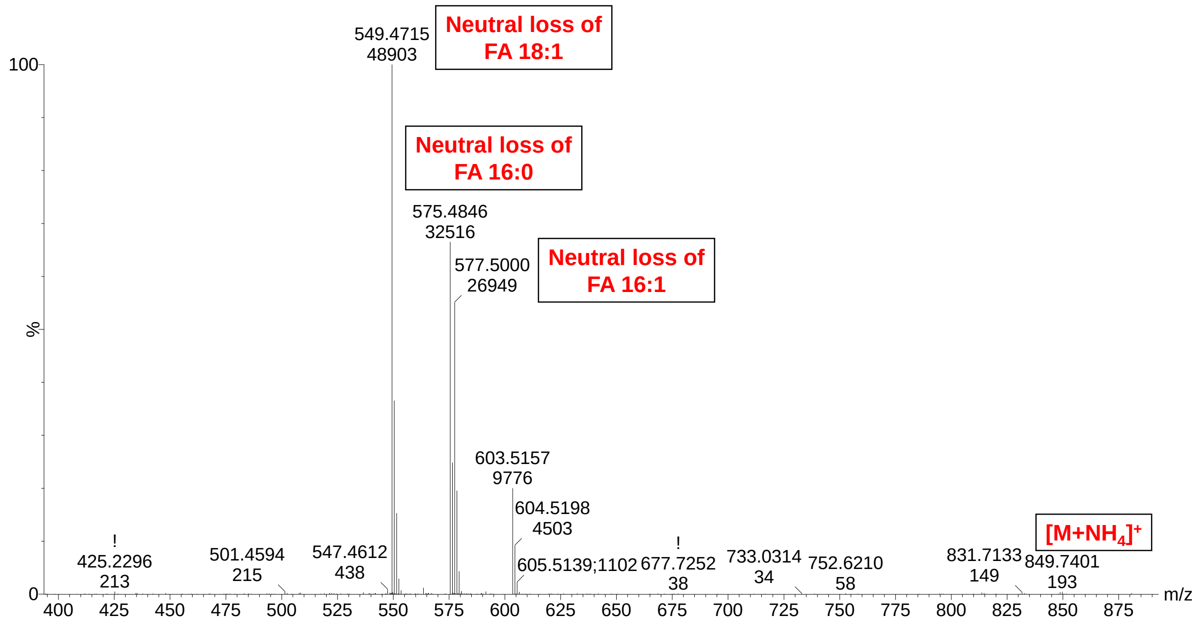

TG 50:3;O2; TG 18:1_18:2_14:0; ESI+, fast DDA

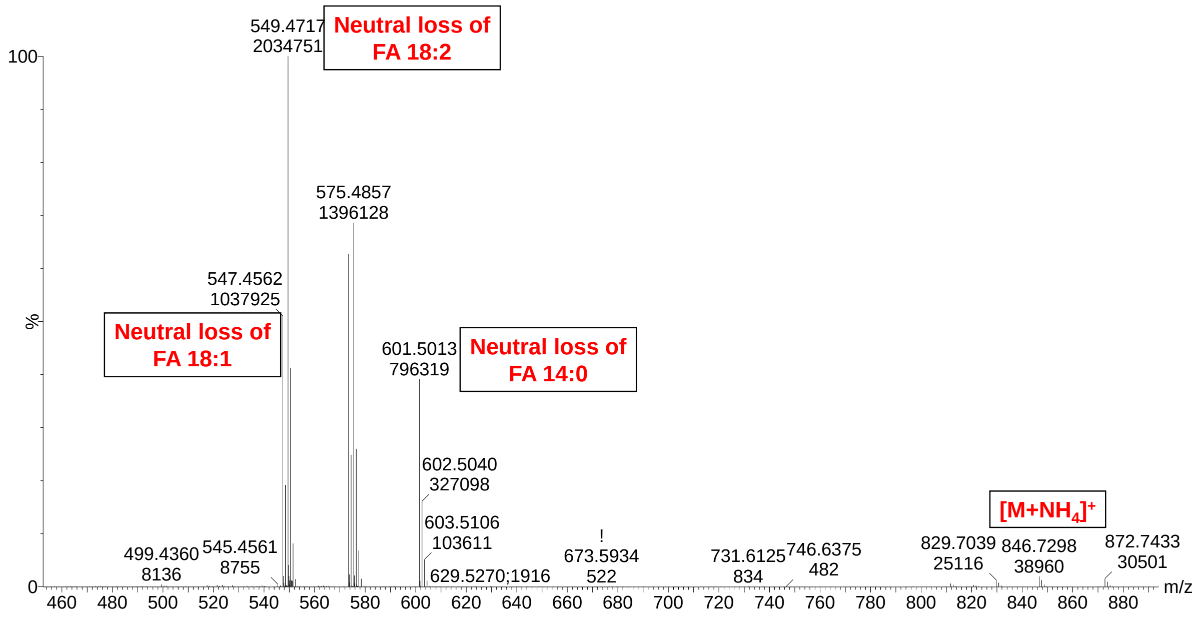

TG 50:3;O2; TG 16:1_16:0_18:2; ESI+, fast DDA

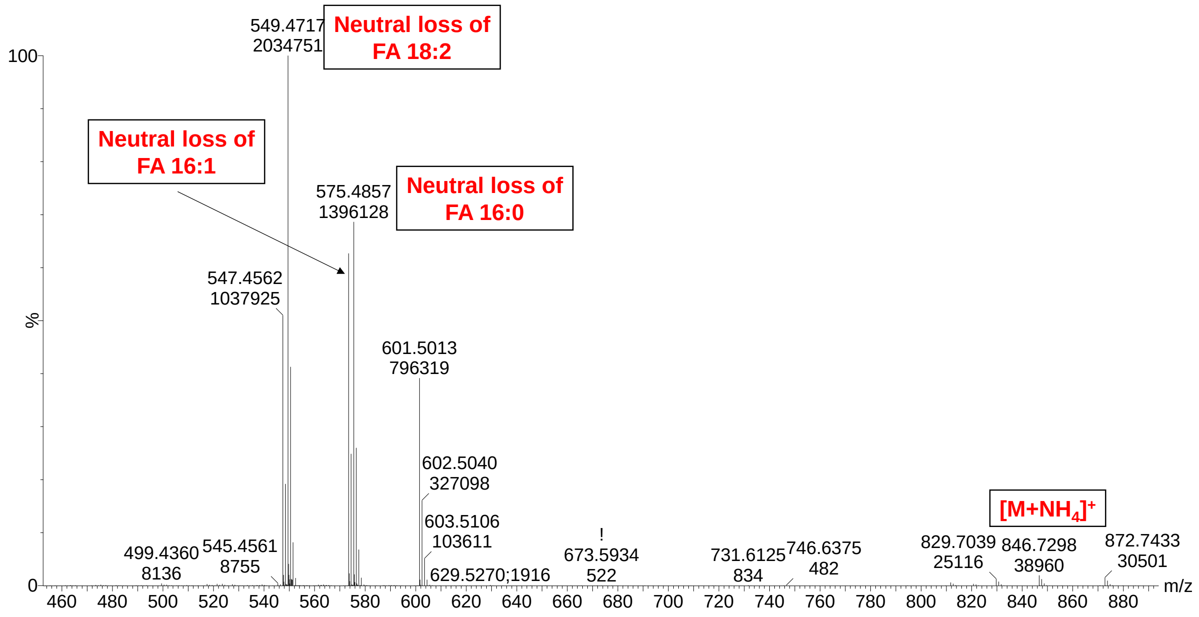

TG 50:3;O2; TG 16:1_16:1_18:1; ESI+, fast DDA

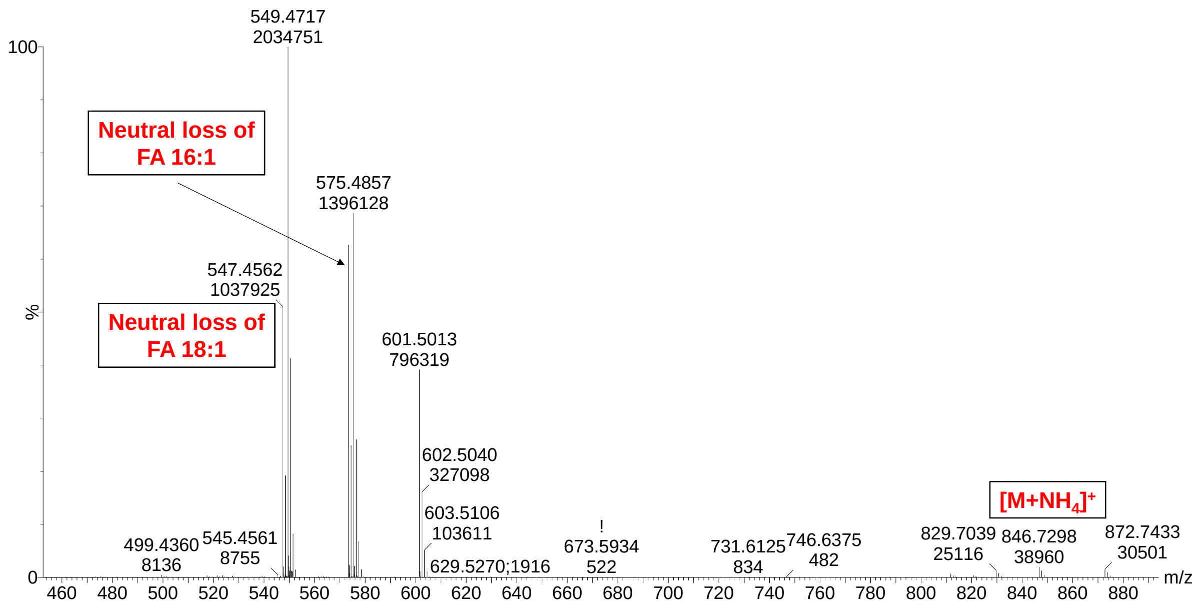

TG 50:4;O2; TG 18:2_16:1_16:1; ESI+, MS^E

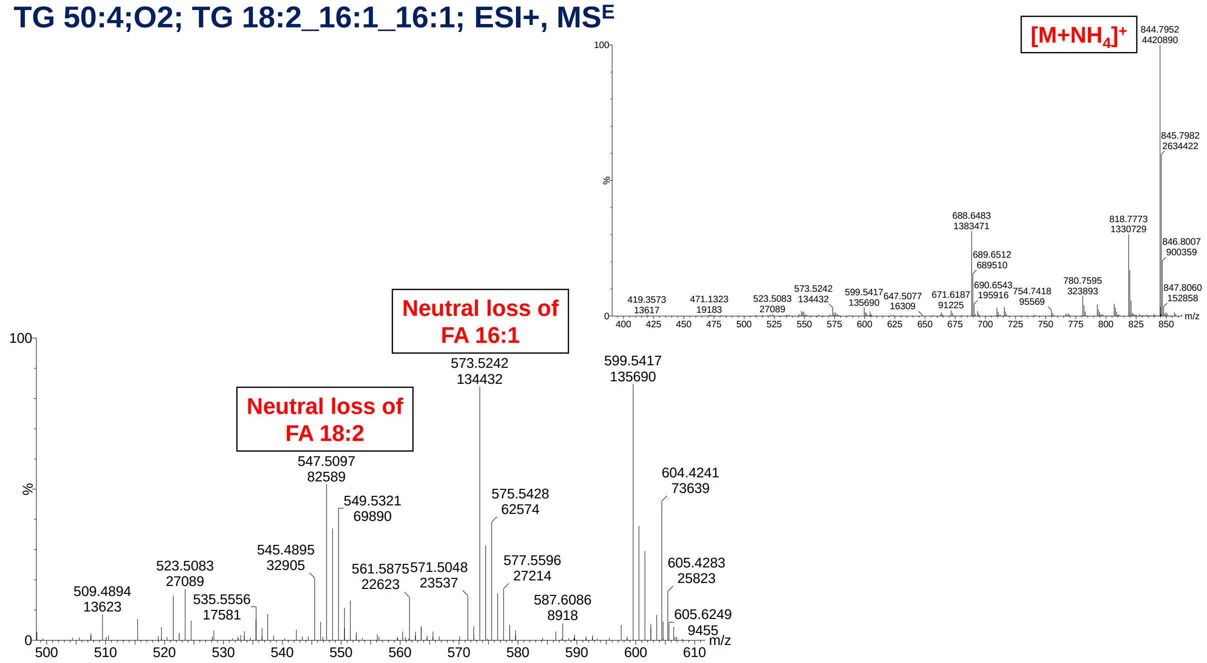

TG 50:4;O2; TG 18:3_16:1_16:0; ESI+, MS^E

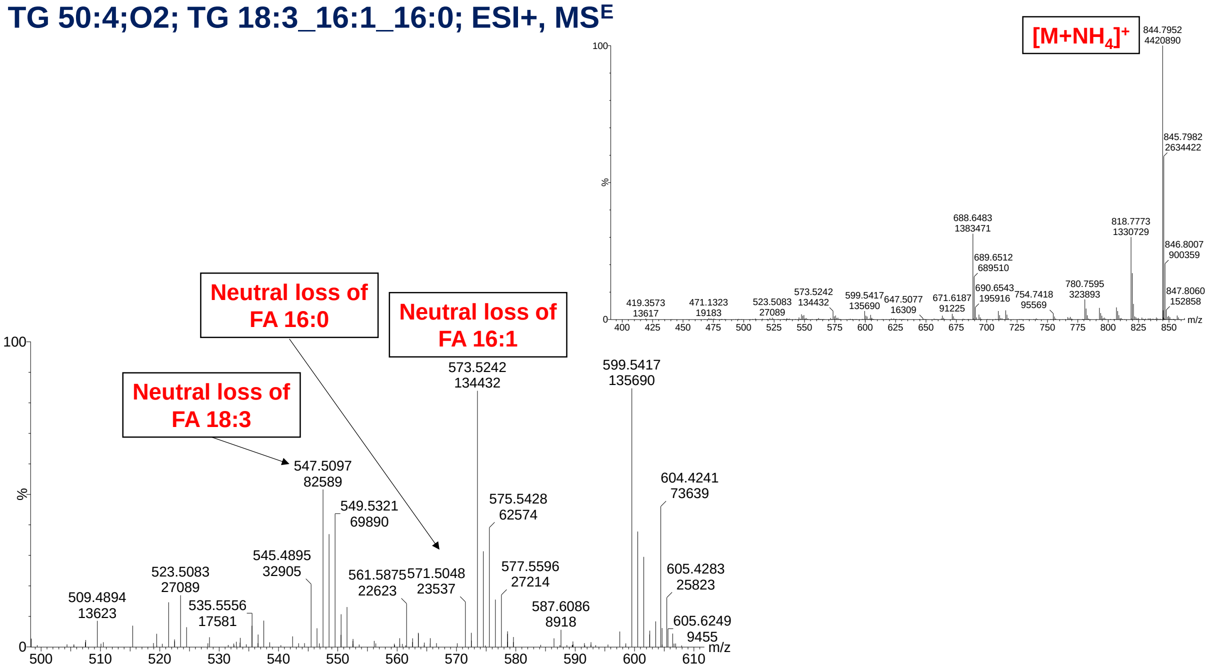

TG 51:3;O2; TG 18:2_15:0_18:1; ESI+, fast DDA

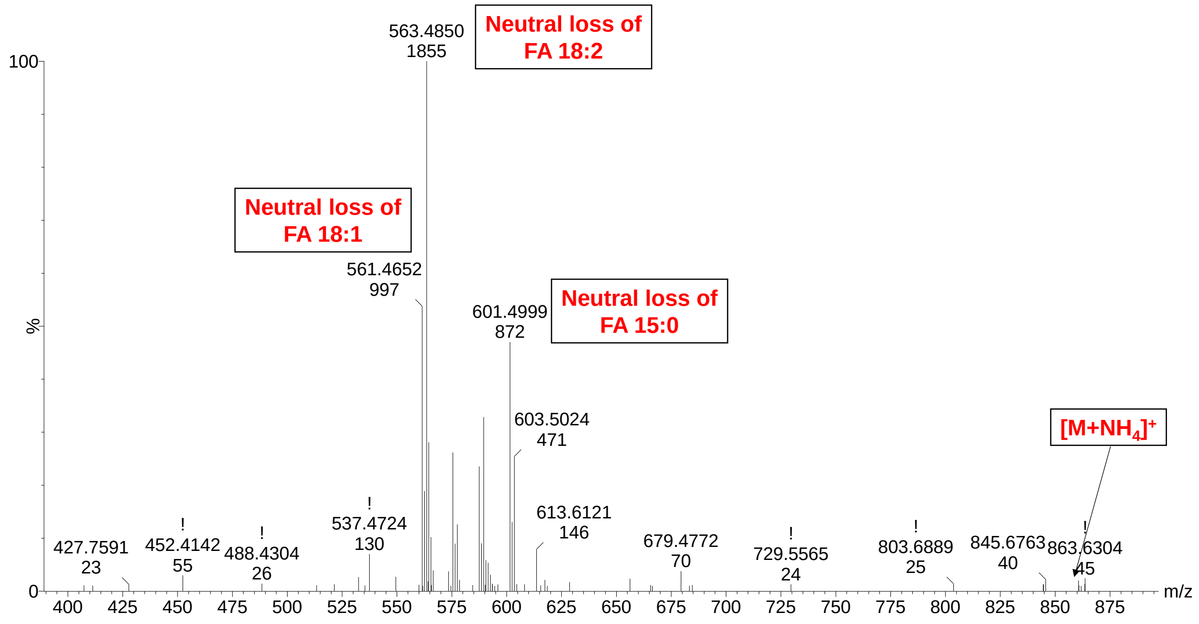

TG 51:3;O2; TG 17:1_18:2_16:0; ESI+, fast DDA

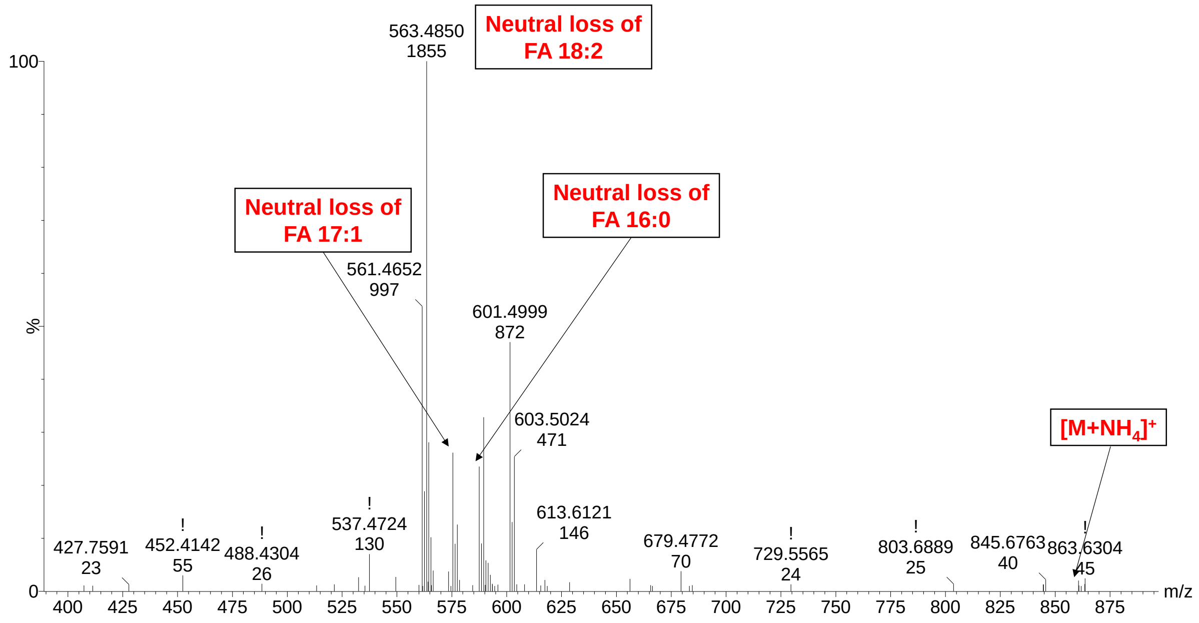

TG 52:3;O2; TG 18:1_18:1_16:1; ESI+, fast DDA

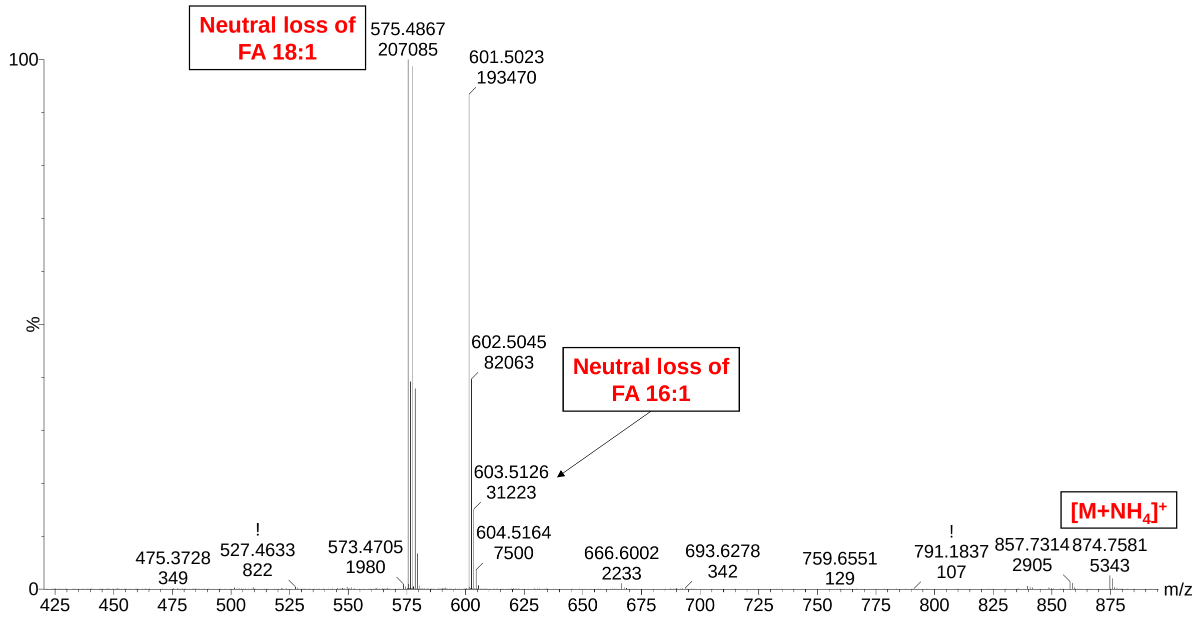

TG 52:3;O2; TG 18:1_18:2_16:0; ESI+, fast DDA

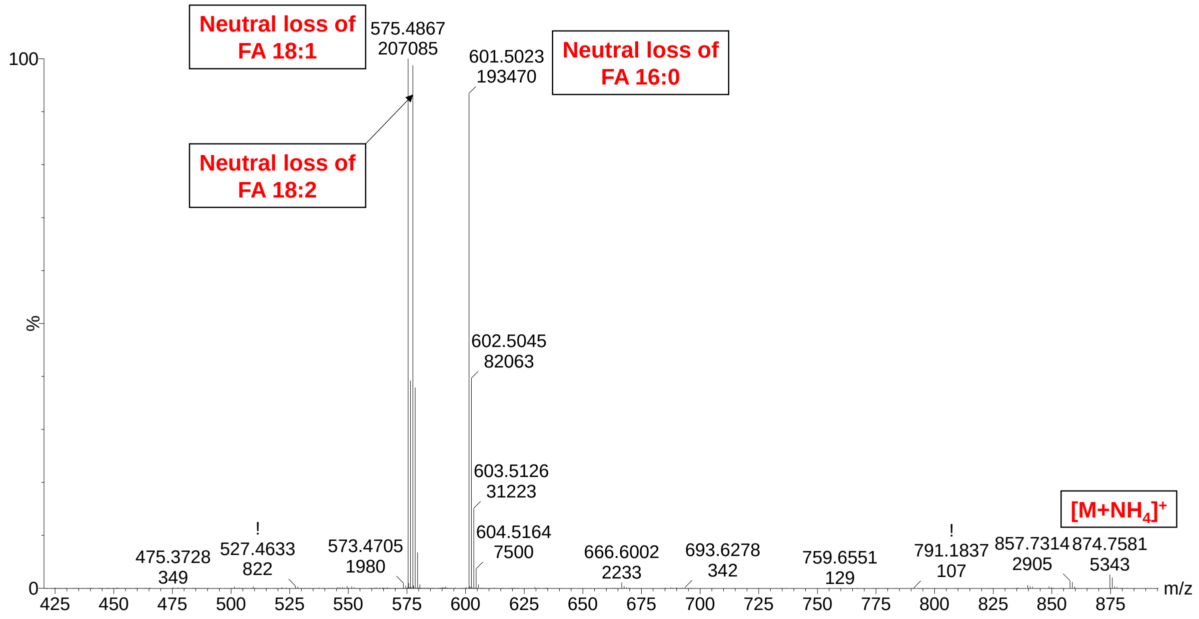

TG 52:4;O2; TG 18:1_18:2_16:1; ESI+, fast DDA

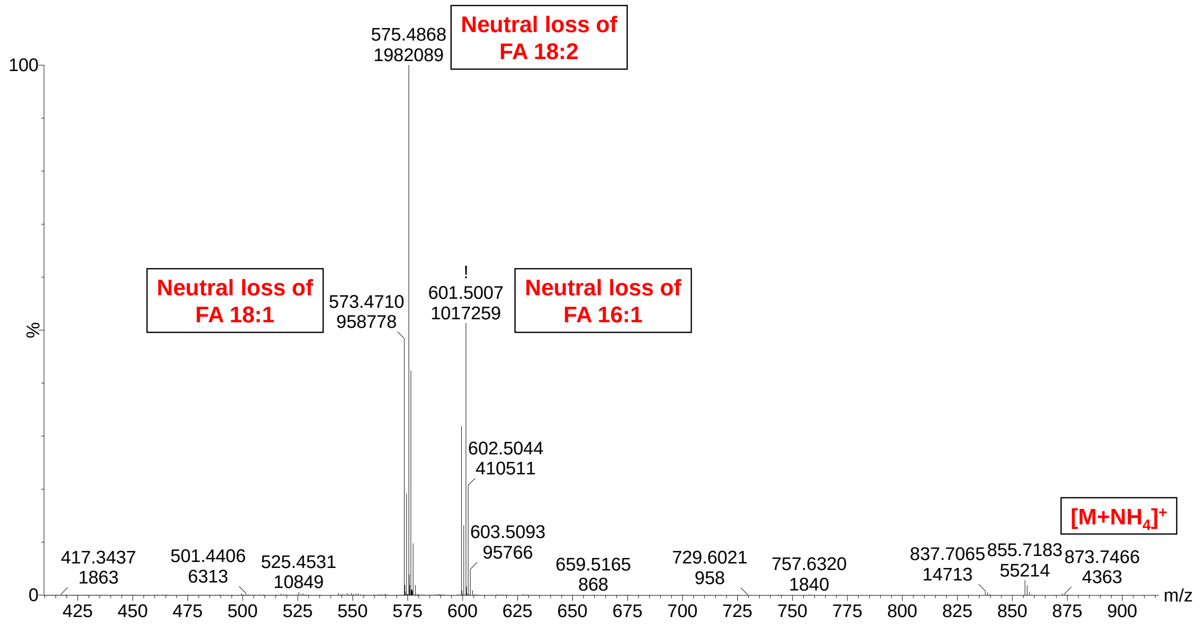

TG 52:4;O2; TG 18:2_18:2_16:0; ESI+, fast DDA

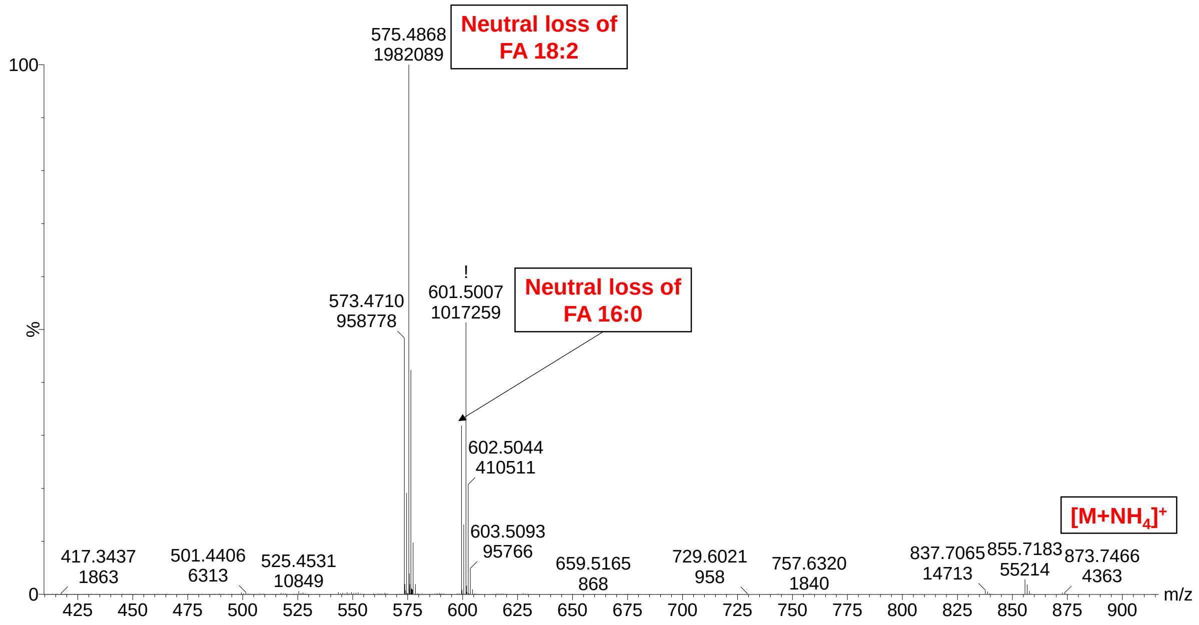

TG 52:5;O2; TG 18:2_18:2_16:1; ESI+, fast DDA

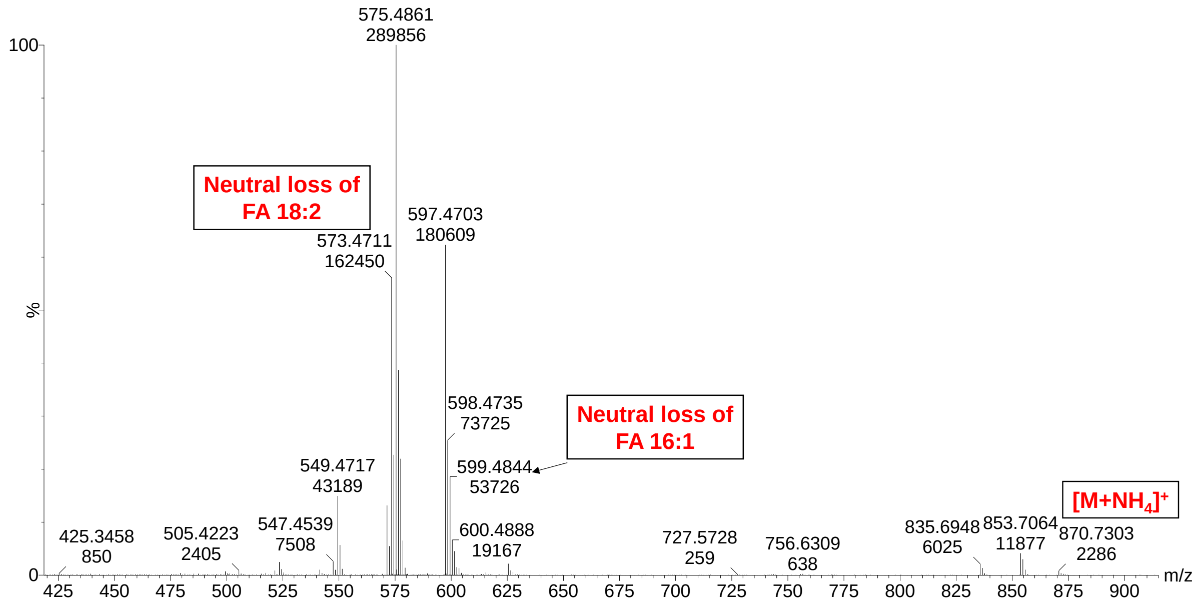

TG 52:5;O2; TG 16:0_16:1_20:4; ESI+, fast DDA

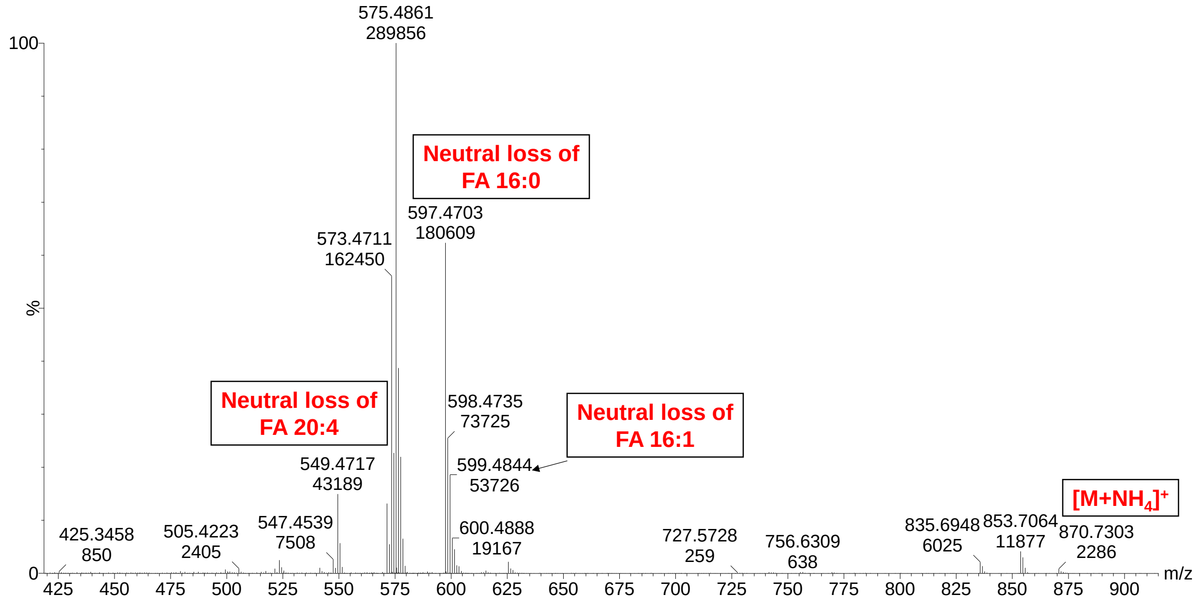

TG 52:5;O2; TG 18:3_18:2_16:0; ESI+, fast DDA

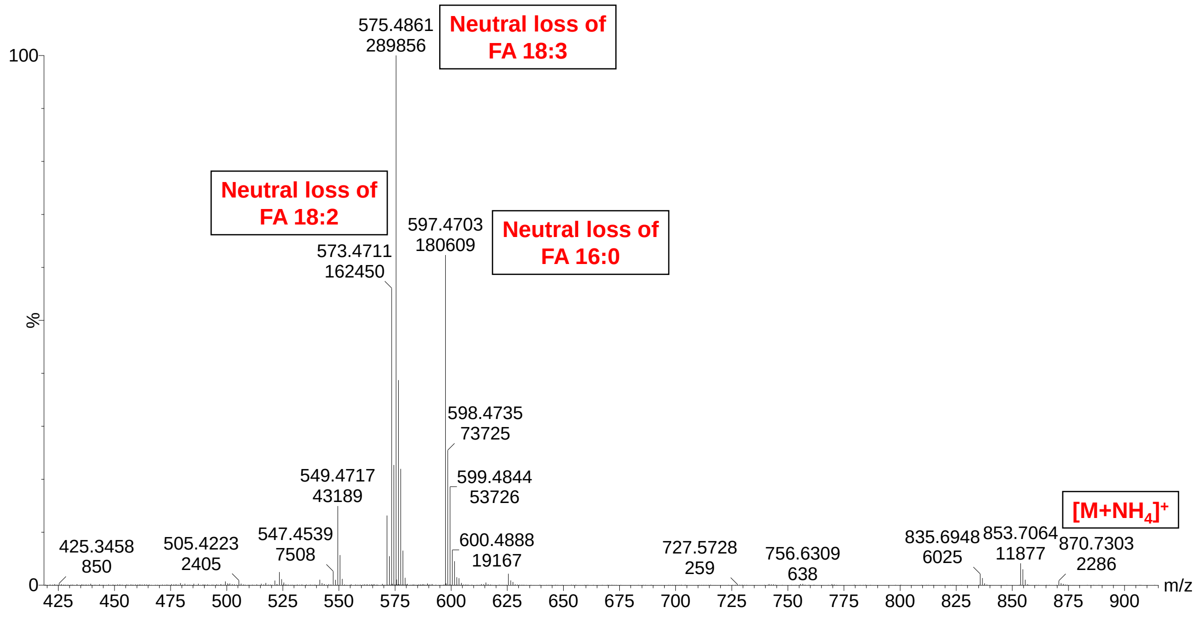

TG 54:1;O2; TG 20:0_18:1_16:0; ESI+, fast DDA

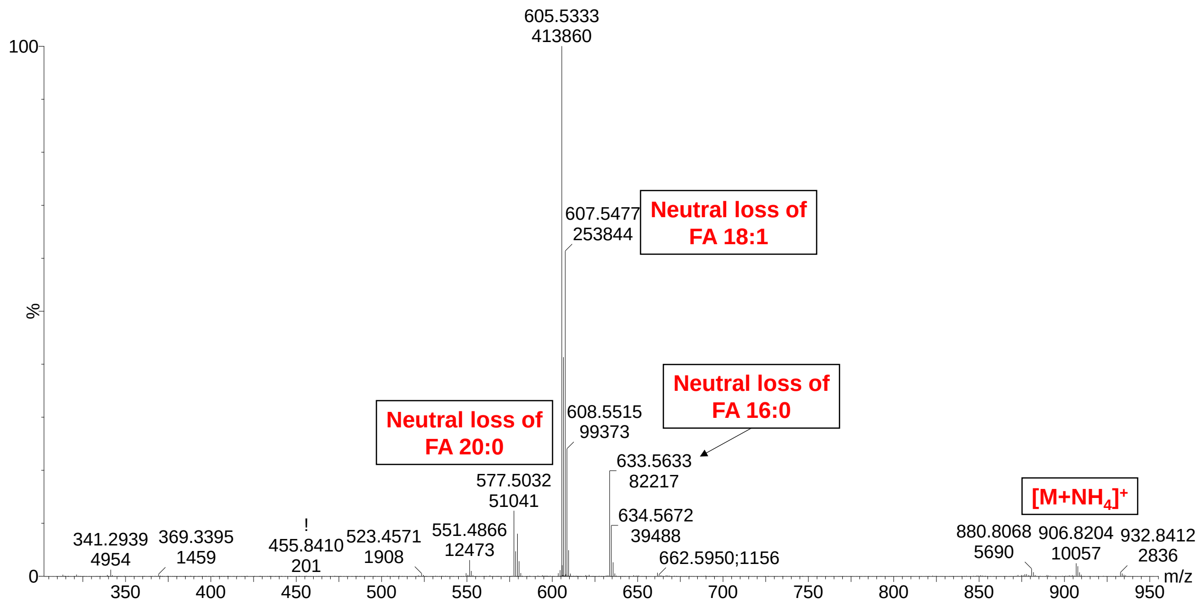

TG 54:1;O2; TG 18:1_18:0_18:0; ESI+, fast DDA

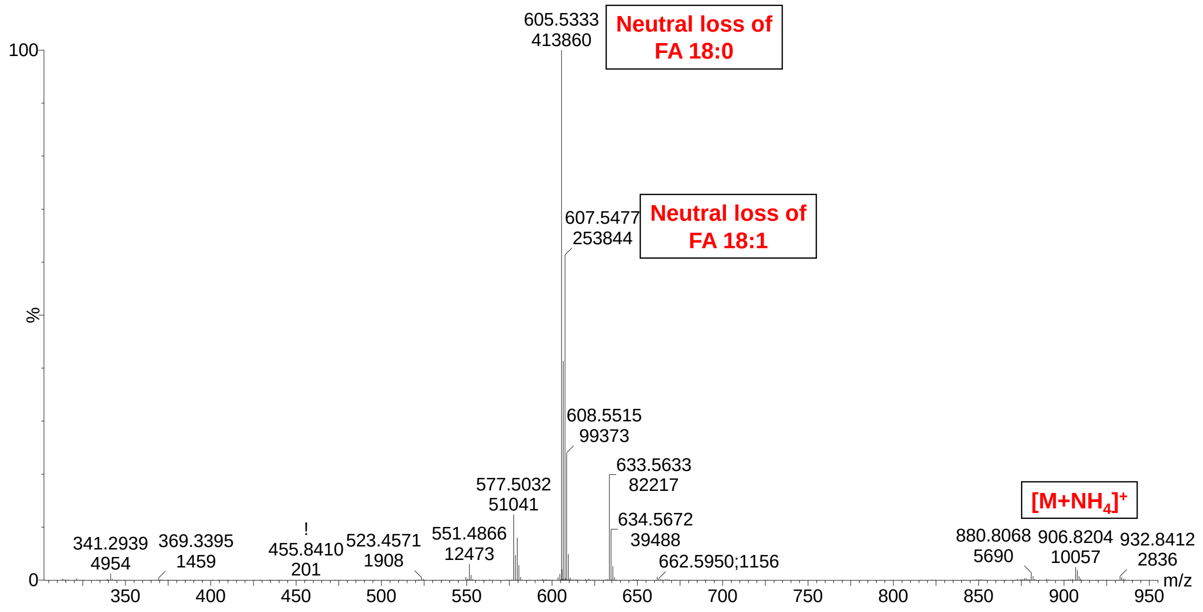

TG 54:1;O2; TG 18:0_20:1_16:0; ESI+, fast DDA

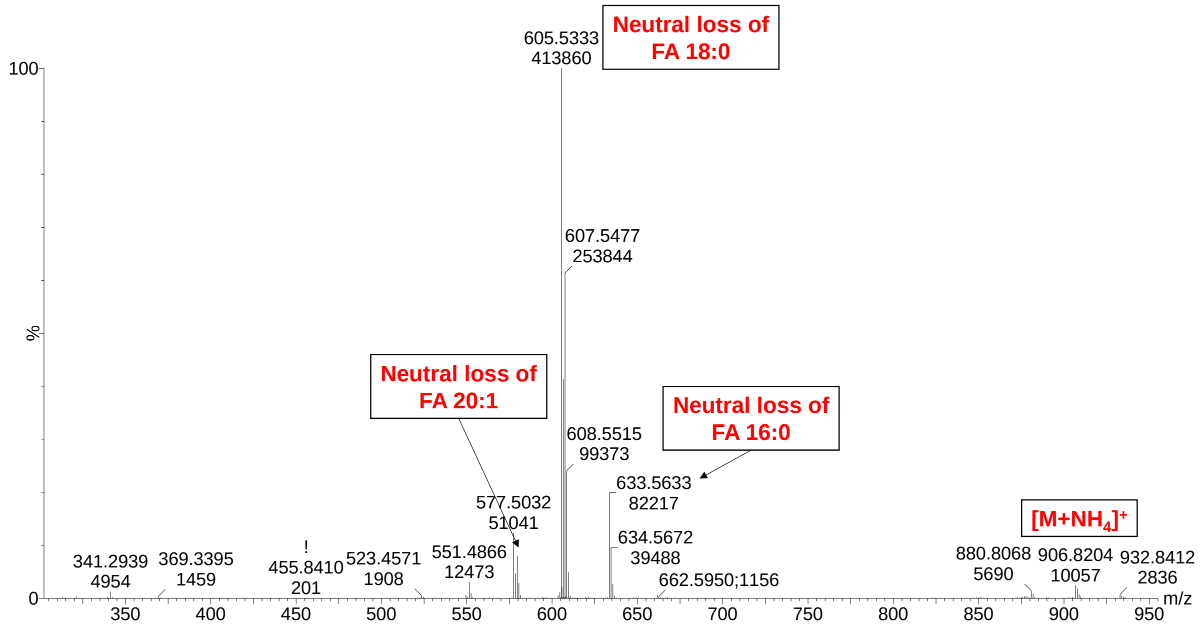

TG 54:2;O2; TG 20:1_18:1_16:0; ESI+, fast DDA

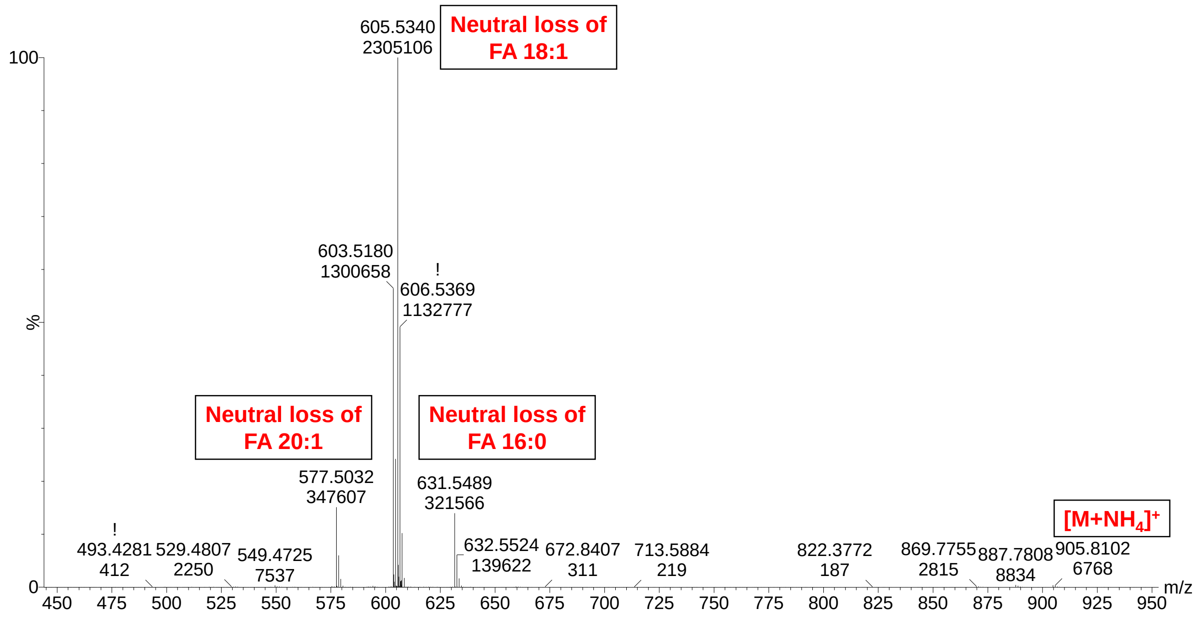

TG 54:2;O2; TG 18:0_18:1_18:1; ESI+, fast DDA

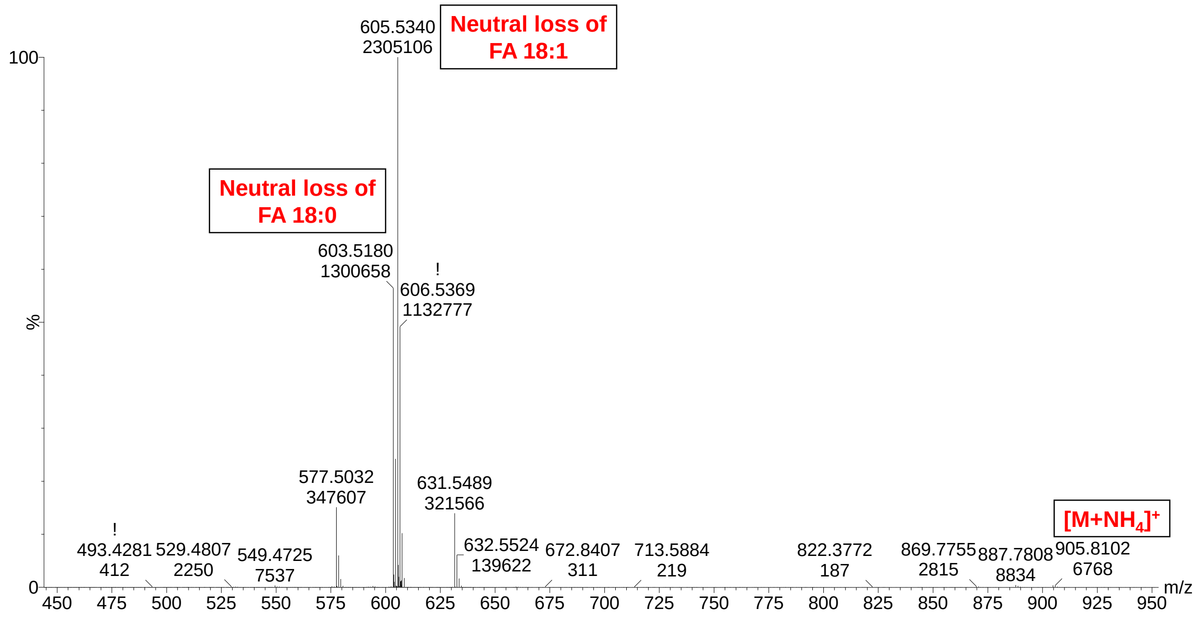

TG 54:3;O2; TG 18:1_18:1_18:1; ESI+, fast DDA

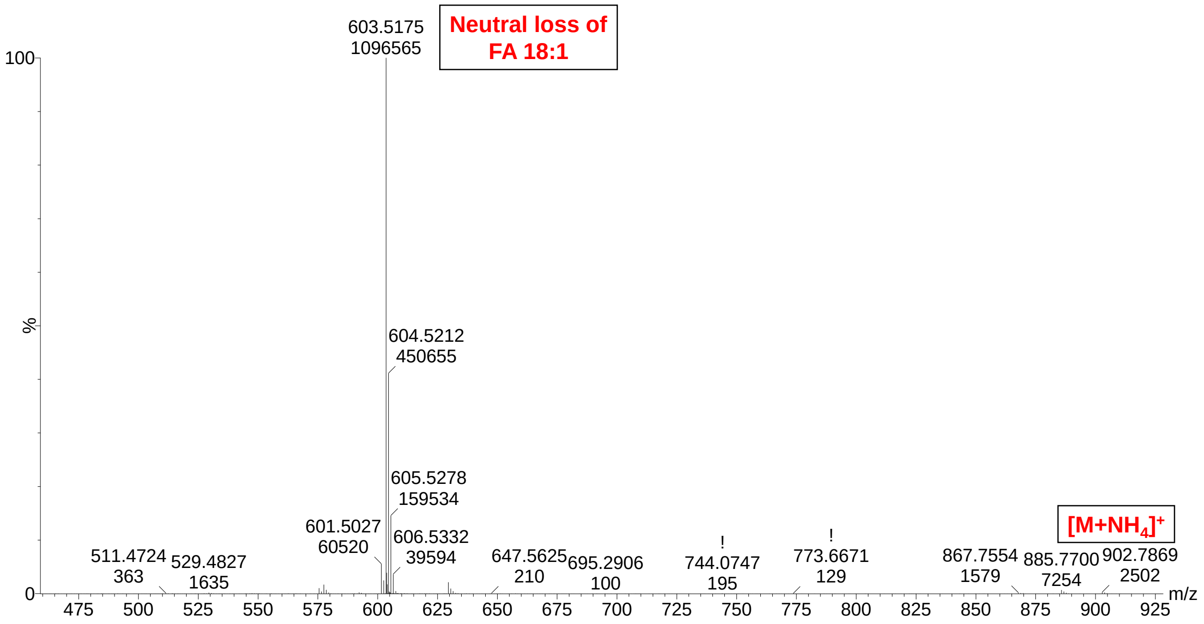

TG 54:3;O2; TG 18:0_18:2_18:1; ESI+, fast DDA

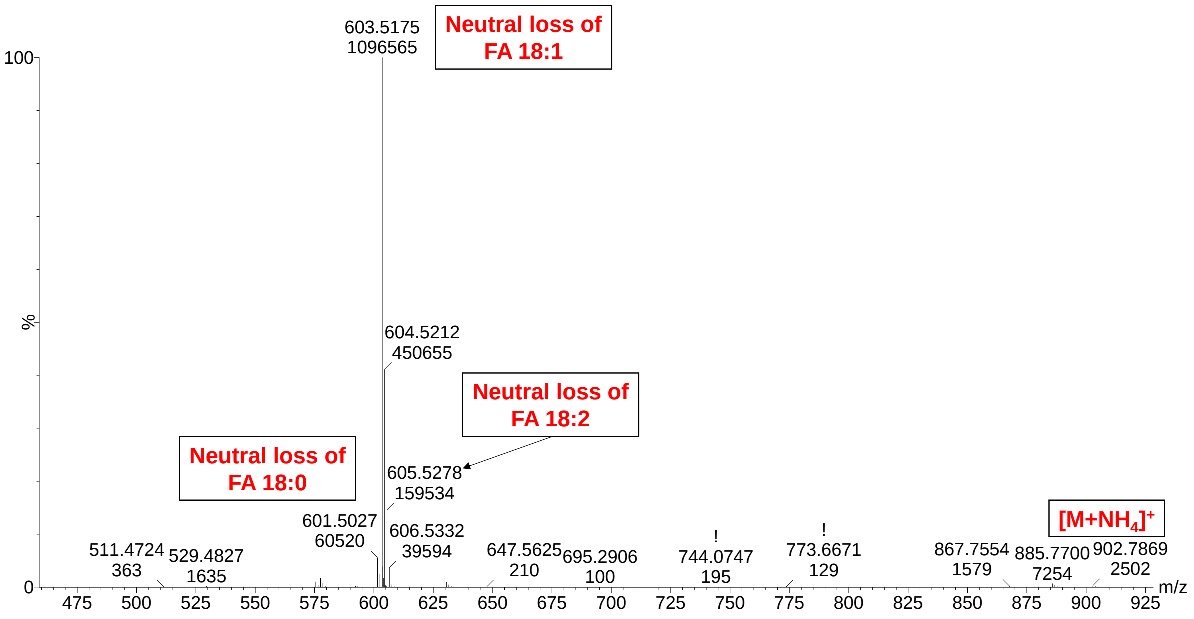

TG 54:4;O2; TG 18:1_18:2_18:1; ESI+, fast DDA

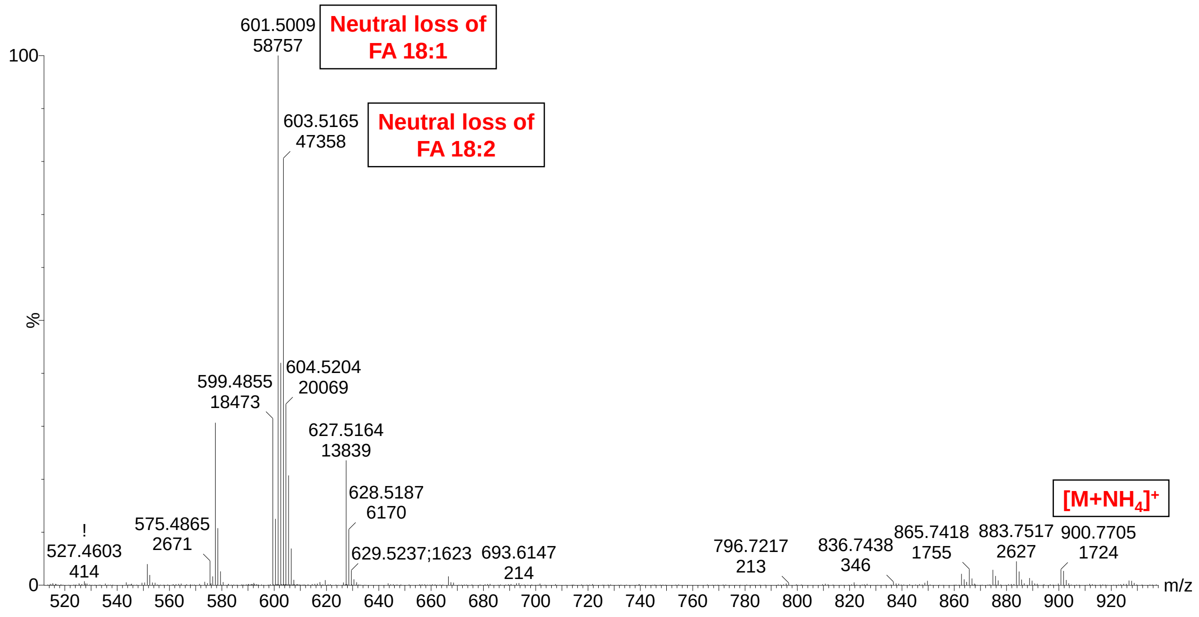

TG 54:4;O2; TG 16:0_18:1_20:3; ESI+, fast DDA

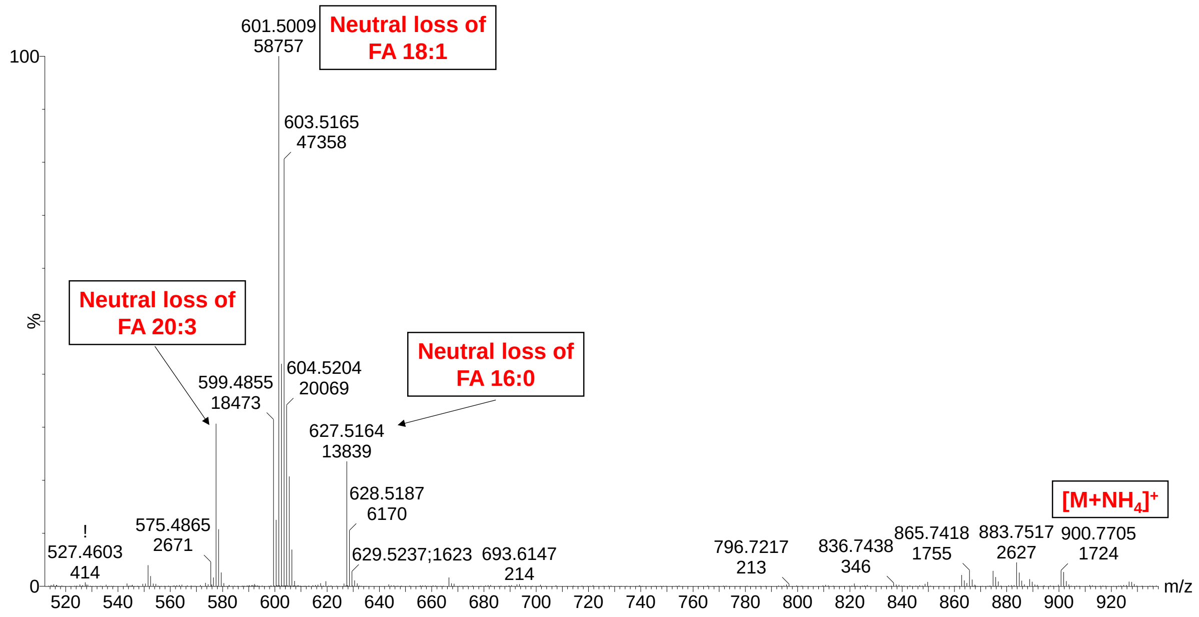

TG 54:4;O2; TG 18:0_18:2_18:2; ESI+, fast DDA

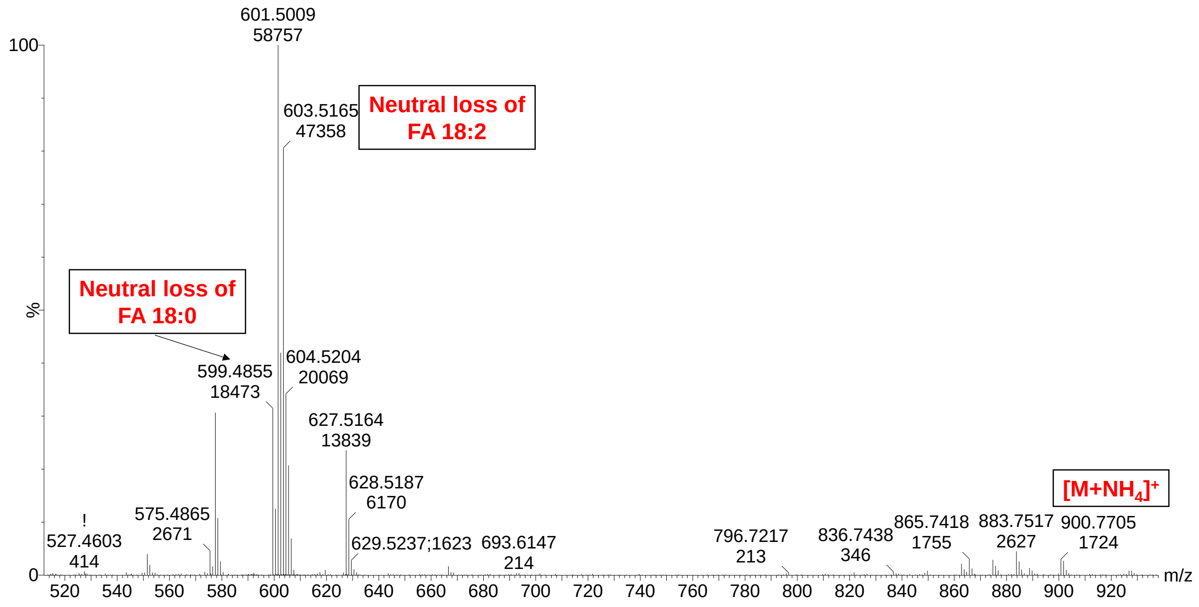

TG 54:5;O2; TG 16:0_18:2_20:3; ESI+, fast DDA

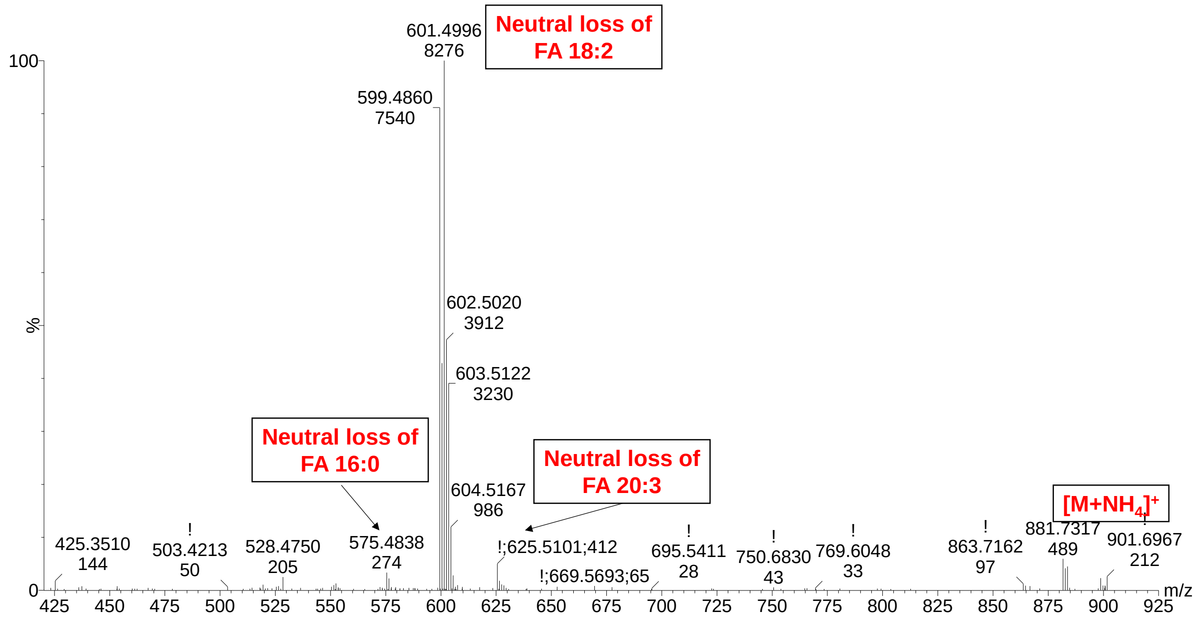

TG 54:5;O2; TG 18:1_18:2_18:2; ESI+, fast DDA

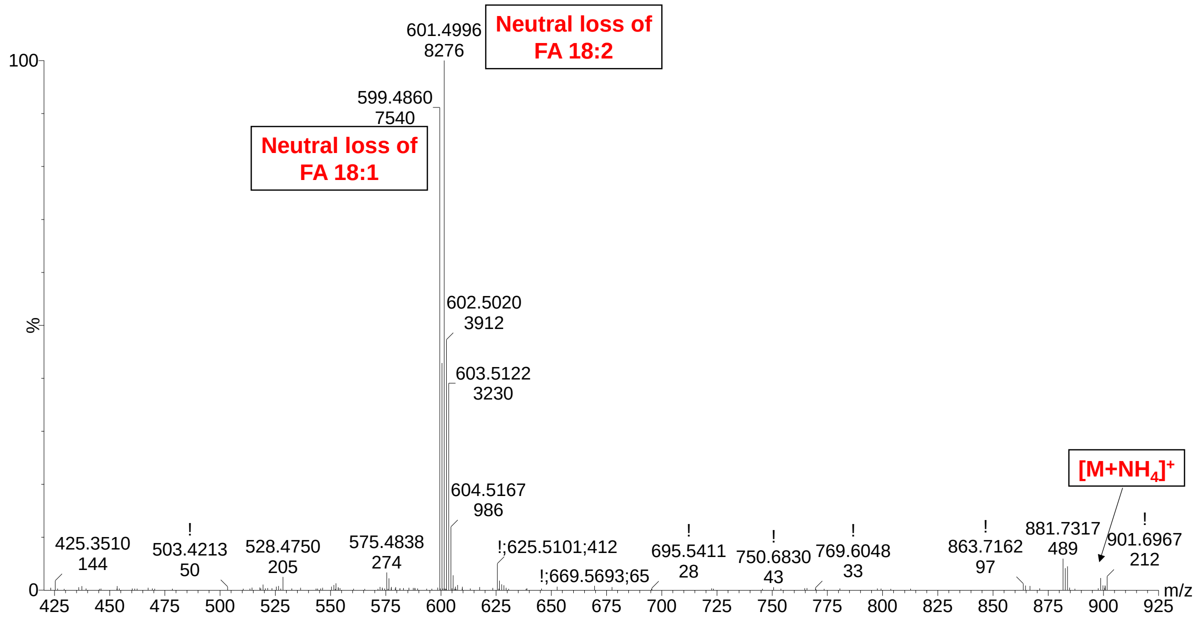

TG 54:5;O2; TG 18:1_18:1_18:3; ESI+, fast DDA

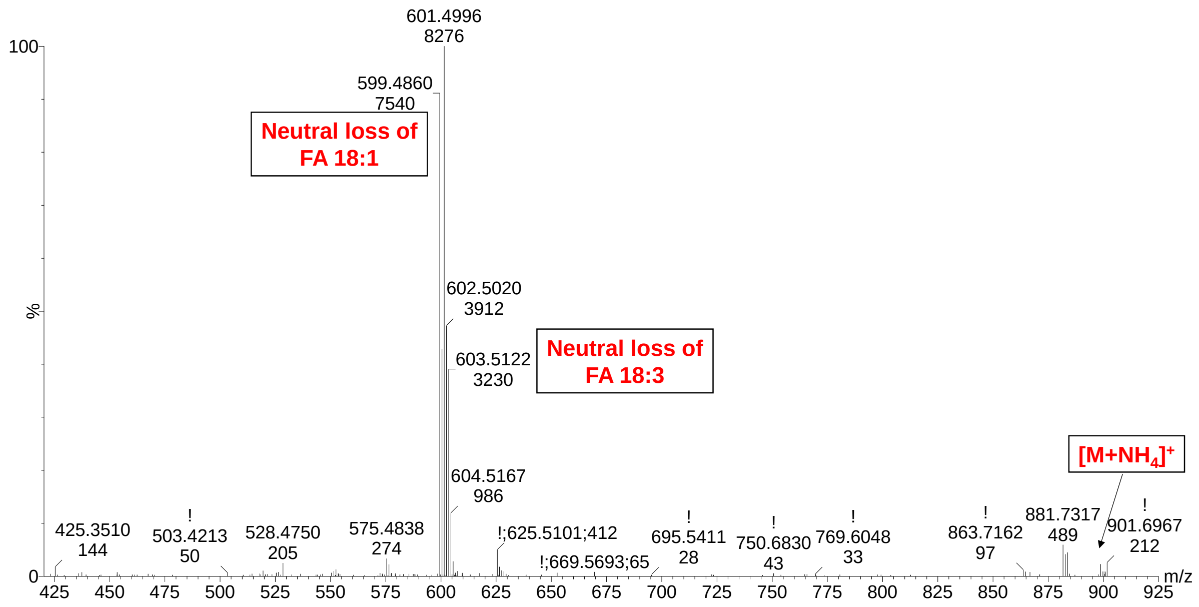

TG 54:6;O2; TG 18:1_18:2_18:3; ESI+, fast DDA

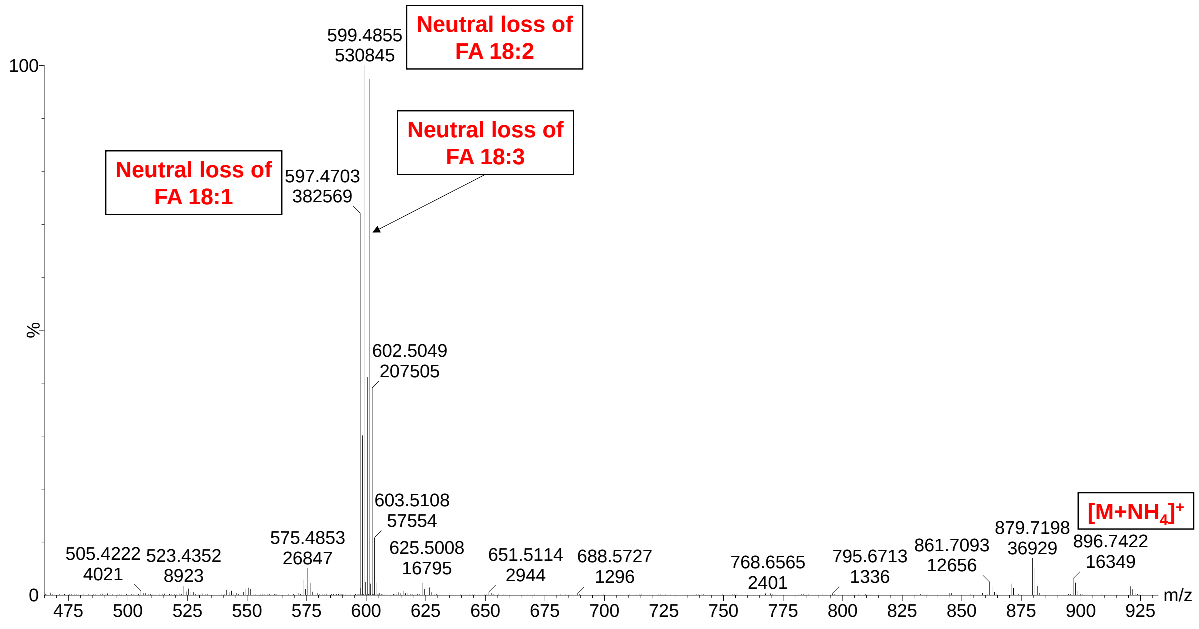

TG 54:6;O2; TG 20:4_18:1_16:1; ESI+, fast DDA

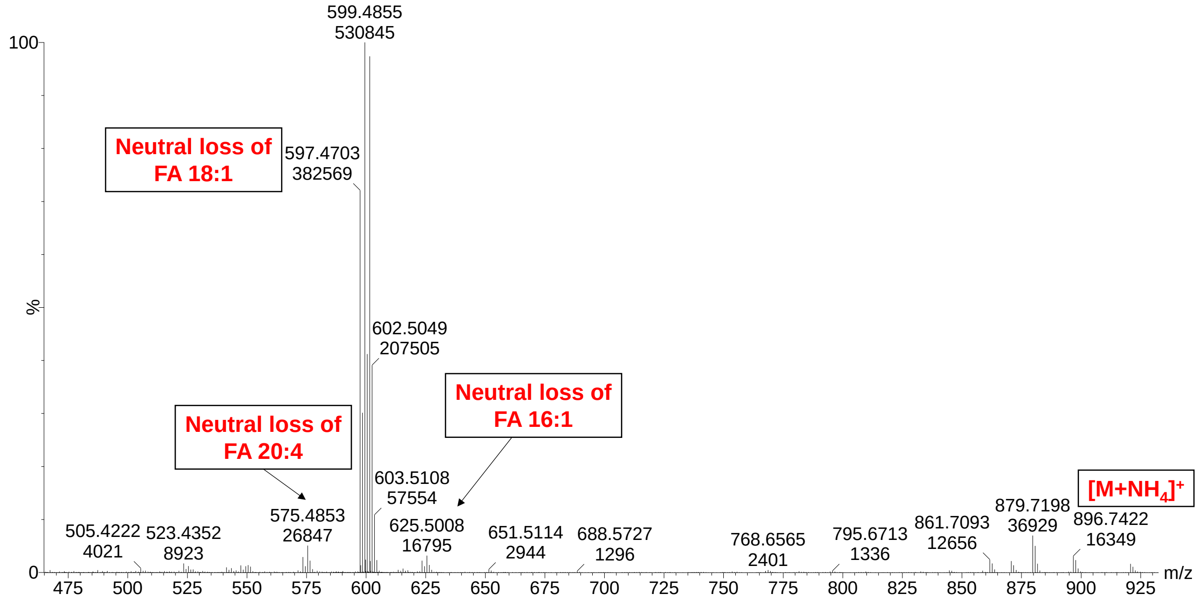

TG 54:6;O2; TG 20:4_18:2_16:0; ESI+, fast DDA

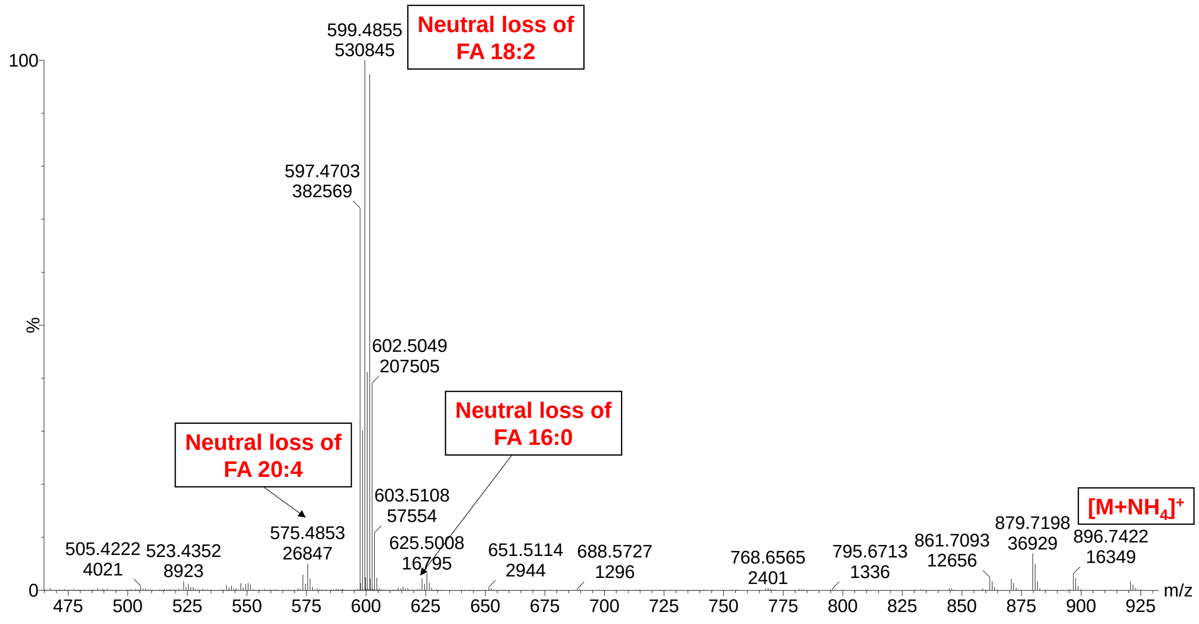

TG 56:2;O2; TG 20:1_18:1_18:0; ESI+, fast DDA

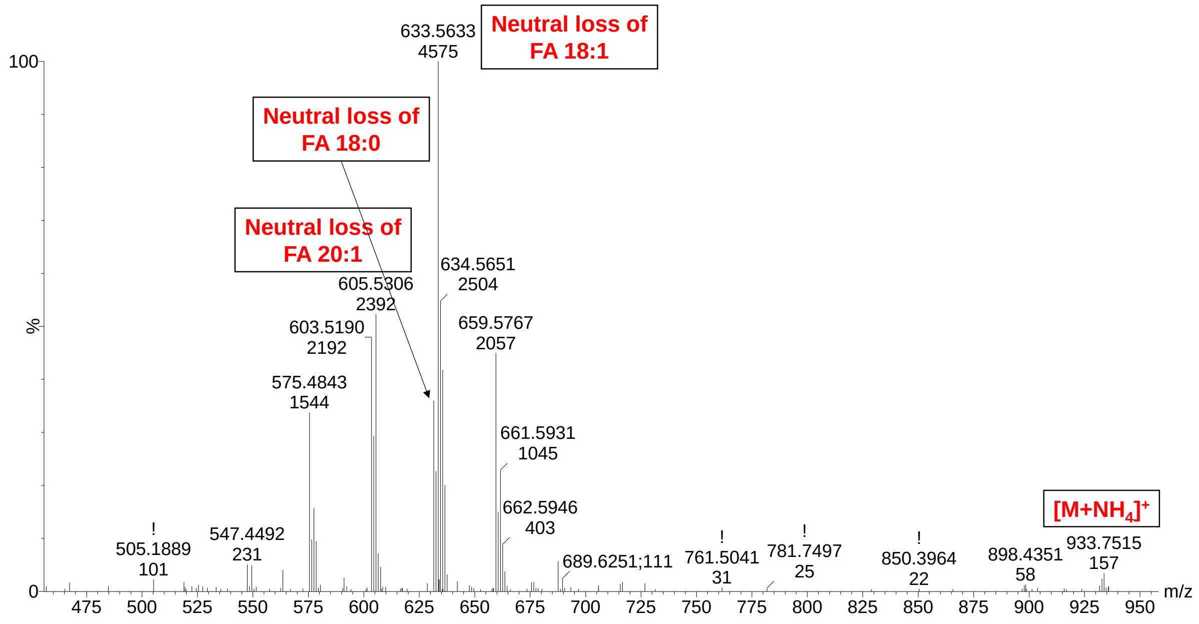

TG 56:2;O2; TG 22:0_18:2_16:0; ESI+, fast DDA

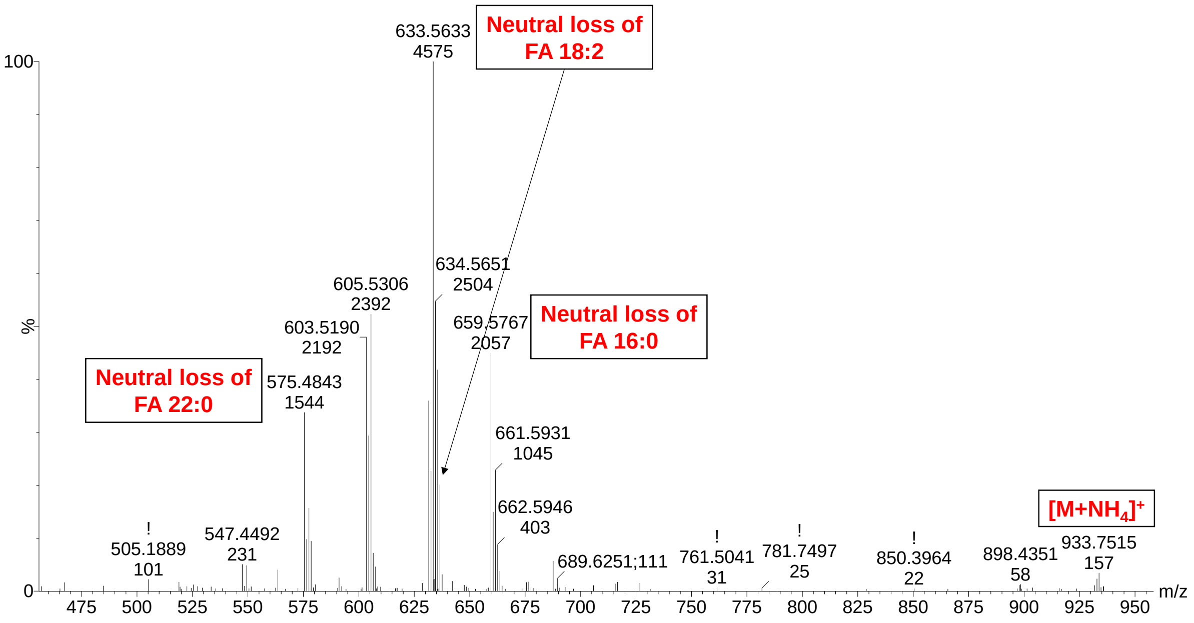

TG 56:5;O2; TG 22:4_16:0_18:1; ESI+, fast DDA

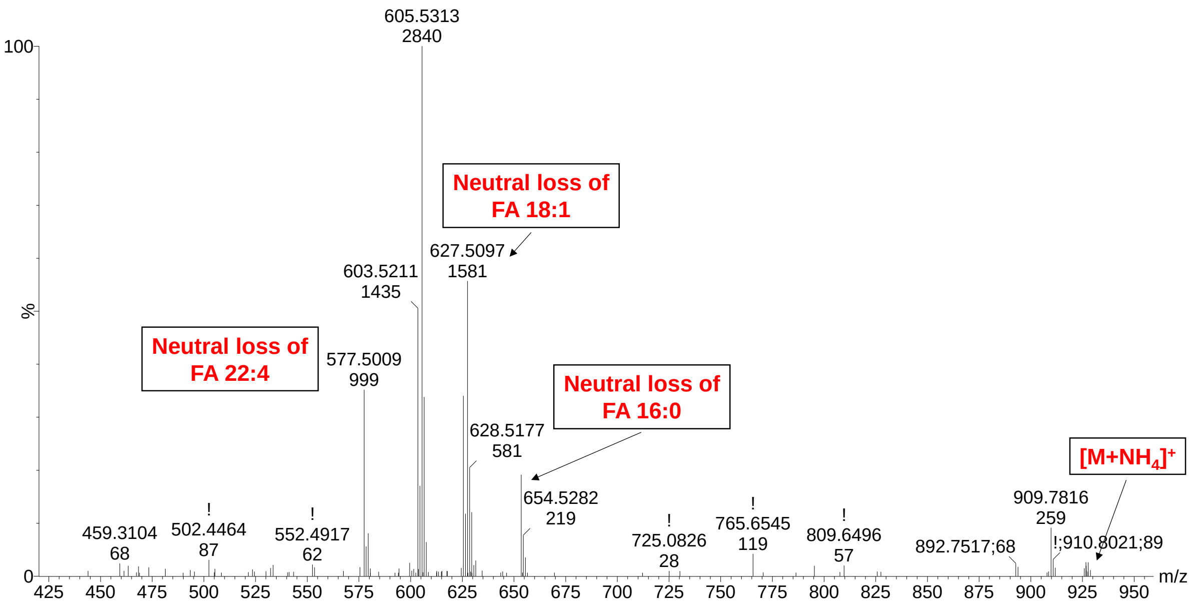

TG 56:5;O2; TG 20:4_18:1_18:0; ESI+, fast DDA

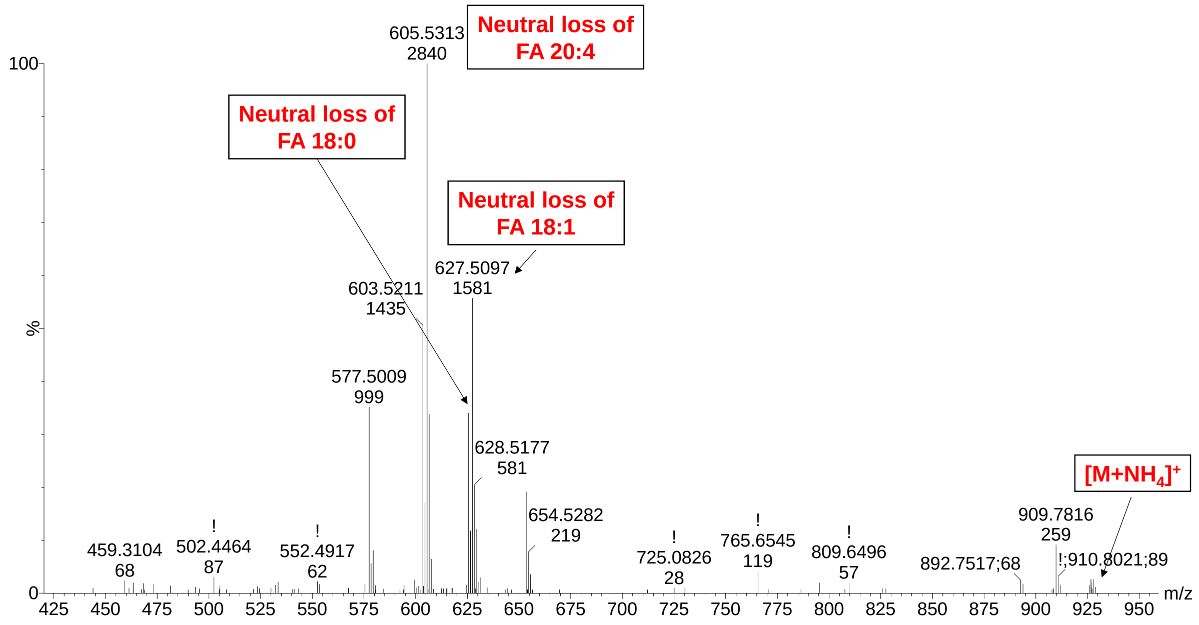

TG 56:6;O2; TG 18:1_18:1_20:4; ESI+, MS^{E}

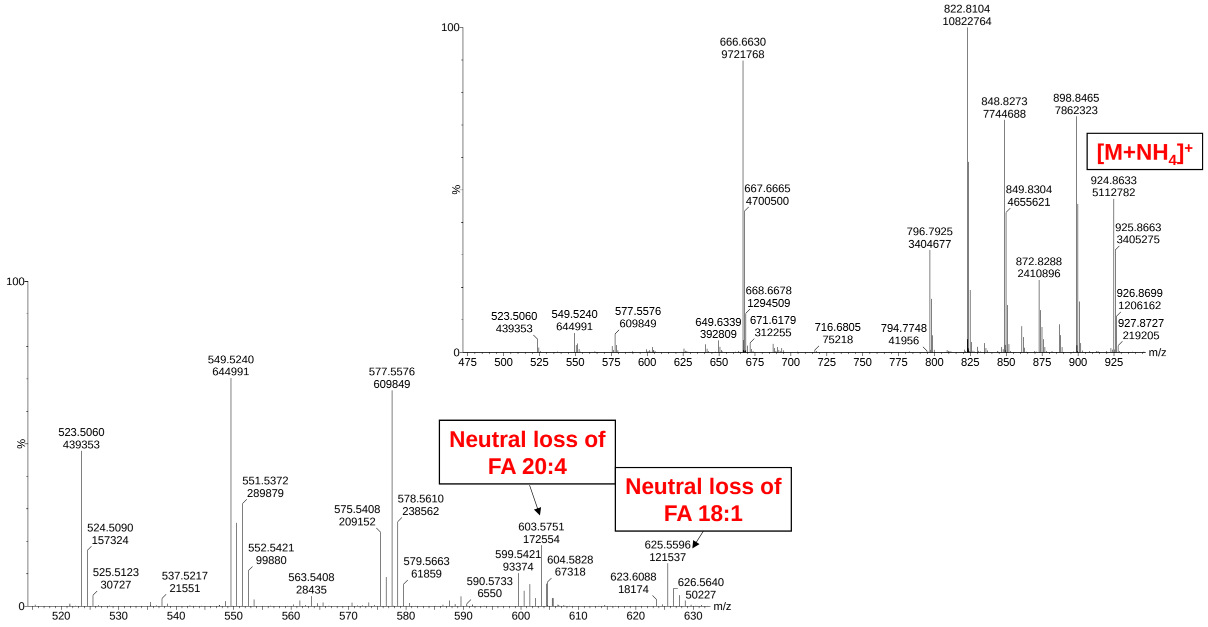

TG 56:6;O2; TG 18:0_18:2_20:4; ESI+, MS^E

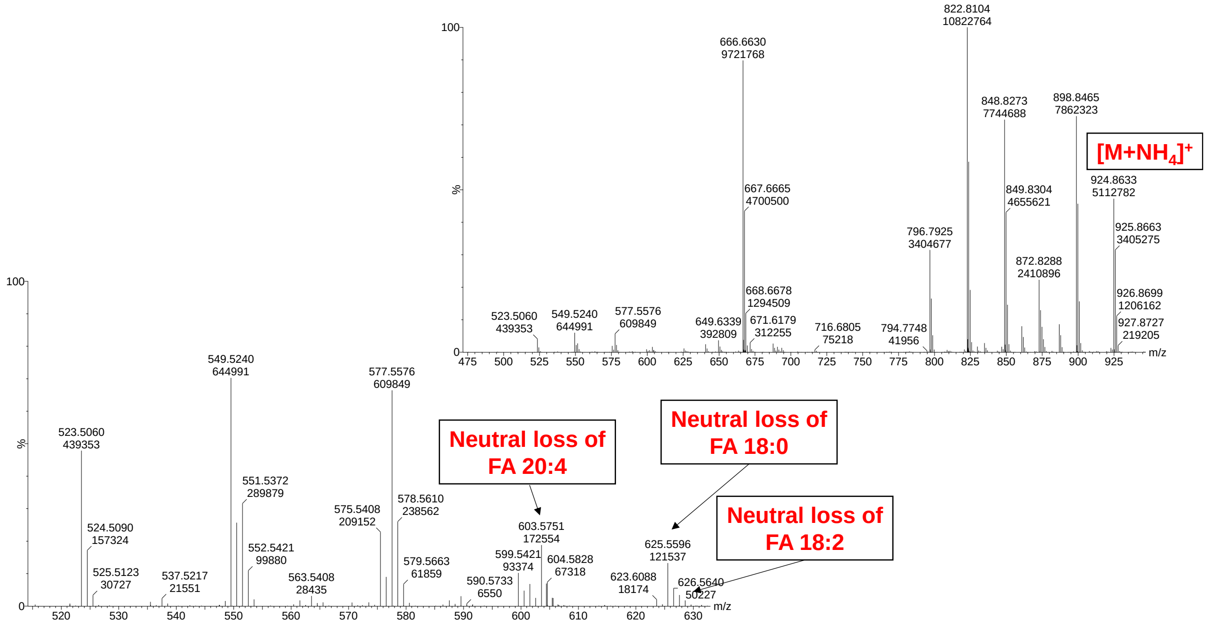

Table S1. Calculation of the lipid standard mix concentrations in body fluid.

Lipid class	Lipid species	Stock conc. [µg/µL]	Volume [µL]	StdMix [µg/mL]	StdMix [µg/mL plasma]	StdMix [nmol/mL plasma]	Exact mass
Cer	Cer 18:1;O2/12:0	2	0.6	1.2	1.0	2.0	481.79
	Cer 18:1;O2-d7/18:0	1	1.4	1.4	1.1	2.0	572.59
HexCer	GlcCer 18:1;O2/12:0	2	0.4	0.8	0.6	1.0	643.94
	GlcCer 18:1;O2-d5/18:0	1	0.9	0.9	0.7	1.0	732.63
Hex2Cer	LacCer 18:1;O2/12:0	2	0.6	1.2	1	1.2	805.55
	LacCer 18:1;O2-d7/15:0	2	0.65	1.3	1	1.2	854.65
GM3	GM3 18:1;O2/16:0-d9	0.5	1.5	0.8	0.6	0.5	1179.50
	GM3 18:1;O2/18:0-d5	0.1	7	0.7	0.6	0.5	1202.80
SM	SM 18:1;O2/12:0	2	14	28.0	22	34.6	646.51
	SM 18:1;O2/18:1 d9	1	32	32.0	26	34.7	737.64
PC	PC 14:0/14:0	4	15.9	63.6	51	75.1	677.50
	PC 15:0/18:1-d7	1	70.5	70.5	56	74.9	752.61
PC P	PC P-36:1 d9	1	9.8	9.8	8	10.0	780.67
LPC	LPC 13:0	2	10	20.0	16	35.3	453.55
	LPC 18:1 d7	1	23	23.0	18	34.8	528.39
PE	PE 14:0/14:0	2	2.4	4.8	4	6.0	635.45
	PE 15:0/18:1-d7	1	5.3	5.3	4	6.0	710.56
PE P	PE P-36:1 d9	1	1.8	1.8	1	1.9	738.62
PI	PI 15:0/18:1	1	6	6.0	5	5.7	839.55
	PI 15:0/18:1-d7	1	6	6.0	5	5.7	846.60
CE	CE 19:0	6	55.5	333.0	266	400	666.63
	CE 16:0 d7	6	45	270.0	216	342.0	631.63
DG	DG 18:1/18:1 d5	1	4.5	4.5	4	5.8	625.57
	DG 15:0/18:1-d7	1	4.2	4.2	3	5.7	587.55
TG	TG 19:1/19:1/19:1	4	16.5	66.0	53	57.0	926.83
	TG 15:0/18:1-d7/15:0	1	57.8	57.8	46	57.0	811.77
Solvent	$CHCl_3$/MetOH (1/1)		606.75				
Total Volume			1000				

Table S2. Compositions of the solvents used for optimization of maximal injection volume in second-dimension.

Solvent	Composition [µL]		
	**24 min**	**58 min**	**96 min**
ACN	150	112	56
H_2O	88	57.6	12.8
IPA	162	230.4	331.2
StdMix	100	100	100

Table S3. List of lipid species identified in pooled human plasma sample with their retention time, and observed ions in positive and negative ESI mode

Lysophosphatidylcholines							
**Lipid species level**	**Lipid acyl/alkyl level**	**ECN**	**D^1 t_R [min]**	**D^2 t_R [min]**	**$[M+H]^+$ (ppm)**	***m/z* 184**	**$[M+formate]^-$ (ppm)**
LPC 16:0	LPC 16:0_0:0	16	4.97	0.31	496.3398 (0.7)	184.0733	540.3307 (-1.5)
LPC 18:0	LPC 18:0_0:0	18	6.46	0.31	524.3711 (1.2)	184.0733	568.3620 (-1.2)
LPC 18:1	LPC 18:1_0:0	16	5.02	0.31	522.3554 (1.7)	184.0733	566.3464 (-2.8)
LPC 18:2	LPC 18:2_0:0	14	4.26	0.31	520.3398 (1.2)	184.0733	564.3307 (-2.1)
LPC 18:3	LPC 18:3_0:0	12	3.47	0.31	518.3241 (1.7)	184.0733	562.3151 (-0.5)
LPC 20:0	LPC 20:0_0:0	20	9.30	0.31	552.4024 (5.3)	184.0733	596.3933 (-2.7)
LPC 20:1	LPC 20:1_0:0	18	6.70	0.31	550.3867 (-2.4)	184.0733	594.3777 (-4.0)
LPC 20:2	LPC 20:2_0:0	16	5.37	0.31	548.3711 (-3.0)	184.0733	592.3620 (2.0)
LPC 20:3	LPC 20:3_0:0	14	4.50	0.31	546.3554 (-1.9)	184.0733	590.3464 (-3.0)
LPC 20:4	LPC 20:4_0:0	12	4.18	0.31	544.3398 (0.1)	184.0733	588.3307 (4.8)
LPC 22:1	LPC 22:1_0:0	20	9.59	0.31	578.418 (-1.4)	184.0733	622.4089 (-3.9)
LPC 22:4	LPC 22:4_0:0	14	4.93	0.31	572.3711 (3.8)	184.0733	616.3620 (2.1)
LPC 22:5	LPC 22:5_0:0	12	4.26	0.31	570.3554 (4.2)	184.0733	614.3464 (3.3)
LPC 22:6	LPC 22:6_0:0	10	4.00	0.31	568.3398 (1.1)	184.0733	612.3307 (2.4)

Fatty acids					
**Lipid species level**	**Lipid acyl/alkyl level**	**ECN**	**D^1 t_R [min]**	**D^2 t_R [min]**	**$[M-H]^-$ (ppm)**
FA 14:0	FA 14:0	14	6.82	0.03	227.2017 (3.5)
FA 15:0	FA 15:0	15	9.20	0.03	241.2173 (3.3)
FA 16:0	FA 16:0	16	12.09	0.03	255.2330 (3.9)
FA 16:1	FA 16:1	14	8.73	0.03	253.2173 (1.6)
FA 17:0	FA 17:0	17	14.91	0.03	269.2486 (3.7)
FA 18:0	FA 18:0	18	18.07	0.03	283.2643 (-3.5)

FA 18:1	FA 18:1	16	12.88	0.03	281.2486 (-3.6)
FA 18:2	FA 18:2	14	9.63	0.03	279.2330 (-2.9)
FA 18:3	FA 18:3	12	7.66	0.03	277.2173 (-1.4)
FA 20:1	FA 20:1	18	18.76	0.03	309.2799 (-4.8)
FA 20:2	FA 20:2	16	13.97	0.03	307.2643 (-1.3)
FA 20:3	FA 20:3	14	10.88	0.03	305.2486 (0.7)
FA 20:4	FA 20:4	12	9.05	0.03	303.2330 (-3.6)
FA 20:5	FA 20:5	10	7.25	0.03	301.2173 (1.3)
FA 22:4	FA 22:4	14	12.32	0.03	331.2643 (-3.0)
FA 22:5	FA 22:5	12	10.55	0.03	329.2486 (-1.2)
FA 22:6	FA 22:6	10	8.56	0.03	327.2330 (4.3)
FA 24:0	FA 24:0	24	50.74	0.03	367.3582 (4.9)

Ceramides

**Lipid species level**	**Lipid acyl/alkyl level**	**ECN**	**D^1 t_R [min]**	**D^2 t_R [min]**	**[M+H-H$_2$O]$^+$ (ppm)**	**N-acyl chain**	**[M+formate]$^-$ (ppm)**
Cer 34:1;O2	Cer 18:1;O2/16:0	32	42.62	0.03	520.5088 (-2.3)	264.2686	582.5103 (0.5)
Cer 36:1;O2	Cer 18:1;O2/18:0	34	50.63	0.03	548.5401 (-2.2)	264.2686	610.5416 (-2.1)
Cer 40:0;O2	Cer 18:0;O2/22:0	40	64.15	0.03	606.6183 (1.3)	266.2842	668.6188 (-2.1)
Cer 40:1;O2	Cer 17:1;O2/23:0	38	62.92	0.03	604.6027 (4.5)	250.2529	666.6042 (4.7)
	Cer 18:1;O2/22:0	38	62.92	0.03	604.6027 (4.5)	264.2686	666.6042 (4.7)
Cer 41:1;O2	Cer 17:1;O2/24:0	39	65.39	0.03	618.6183 (-4.5)	250.2529	680.6199 (-3.5)
	Cer 18:1;O2/23:0	39	65.39	0.03	618.6183 (-4.5)	264.2686	680.6199 (-3.5)
Cer 41:2;O2	Cer 17:1;O2/24:1	37	61.07	0.03	616.6027 (3.1)	250.2529	678.6042 (-0.7)
	Cer 18:2;O2/23:0	37	61.07	0.03	616.6027 (3.1)	262.2529	678.6042 (-0.7)
Cer 42:1;O2	Cer 17:1;O2/25:0	40	67.26	0.03	632.6340 (-1.3)	250.2529	694.6355 (-3.5)
	Cer 18:1;O2/24:0	40	67.26	0.03	632.6340 (-1.3)	264.2686	694.6355 (-3.5)
	Cer 20:1;O2/22:0	40	67.26	0.03	632.6340 (-1.3)	292.2999	694.6355 (-3.5)
Cer 42:2;O2	Cer 18:2;O2/24:0	38	62.36	0.03	630.6183 (0.5)	262.2529	692.6199 (-5.0)
	Cer 19:1;O2/23:1	38	62.36	0.03	630.6183 (0.5)	278.2842	692.6199 (-5.0)
Cer 42:3;O2	Cer 18:2;O2/24:1	36	59.22	0.03	628.4724 (1.4)	262.2529	690.6042 (-1.0)

Cer 43:1;O2	Cer 18:1;O2/25:0	41	68.5	0.03	646.6496 (-3.2)	264.2686	708.6512 (-1.8)
	Cer 19:1;O2/24:0	41	68.5	0.03	646.6496 (-3.2)	278.2842	708.6512 (-1.8)

Monohexosylceramides

Lipid species level	Lipid acyl/alkyl level	ECN	D^1 t_R [min]	D^2 t_R [min]	$[M+H]^+$ (ppm)	N-acyl chain	$[M+formate]^-$ (ppm)
HexCer 34:1;O2	HexCer 18:1;O2/16:0	32	34.25	0.10	700.5722 (0.1)	264.2686	744.5632 (2.4)
HexCer 40:1;O2	HexCer 18:1;O2/22:0	38	60.25	0.10	784.6661 (1.1)	264.2686	828.6569 (-1.9)
HexCer 41:1;O2	n.d.	39	63.18	0.10	798.6818 (-1.6)	-	842.6727 (4.9)
HexCer 42:1;O2	n.d.	40	66.03	0.10	812.6974 (4.7)	-	856.6884 (2.5)
HexCer 42:2;O2	n.d.	38	60.12	0.10	810.6818 (4.7)	-	854.6727 (2.5)

Dihexosylceramised

Lipid species level	Lipid acyl/alkyl level	ECN	D^1 t_R [min]	D^2 t_R [min]	$[M+H]^+$ (ppm)	$[M+formate]^-$ (ppm)
Hex2Cer 32:1;O2	n.d.	30	20.57	0.13	834.5937 (-3.0)	878.5847 (3.2)
Hex2Cer 34:1;O2	n.d.	32	27.99	0.12	862.6250 (1.2)	906.6159 (-1.2)
Hex2Cer 40:1;O2	n.d.	38	53.35	0.12	946.7189 (-2.4)	990.7099 (1.0)
Hex2Cer 42:1;O2	n.d.	40	58.92	0.12	974.7502 (0.9)	1018.7412 (1.2)
Hex2Cer 42:2;O2	n.d.	38	52.73	0.12	972.7346 (2.7)	1016.7255 (1.2)
Hex2Cer 42:3;O2	n.d.	36	47.16	0.12	970.7233 (-0.8)	1014.7099 (2.3)

GM3 gangliosides

Lipid species level	Lipid acyl/alkyl level	ECN	D^1 t_R [min]	D^2 t_R [min]	$[M-H]^-$ (ppm)
GM3 34:1;O2	n.d.	32	20.01	0.19	1151.7059 (-3.1)
GM3 36:1;O2	n.d.	34	26.82	0.19	1179.7372 (-4.0)
GM3 36:2;O2	n.d.	32	20.64	0.19	1177.7215 (-4.2)
GM3 38:1;O2	n.d.	36	35.48	0.19	1207.7685 (-0.5)
GM3 38:2;O2	n.d.	34	31.51	0.19	1205.7528 (-3.6)
GM3 40:1;O2	n.d.	38	43.51	0.19	1235.7998 (-4.8)
GM3 40:2;O2	n.d.	36	37.33	0.19	1233.7841 (-1.6)
GM3 42:1;O2	n.d.	40	50.94	0.19	1263.8311 (-4.0)
GM3 42:2;O2	n.d.	38	43.51	0.19	1261.8154 (-3.0)

GM3 42:3;O2	n.d.	36	36.71	0.19	1259.7998 (-4.7)

Sphingomyelins

Lipid species level	Lipid acyl/alkyl level	ECN	D^1 t_R [min]	D^2 t_R [min]	[M+H]$^+$ (ppm)	*m/z* 184	[M+formate]$^-$ (ppm)
SM 32:1;O2	n.d.	30	20.95	0.28	675.5436 (2.8)	184.0733	719.5346 (4.9)
SM 33:1;O2	n.d.	31	24.71	0.28	689.5592 (0.9)	184.0733	733.5502 (-1.3)
SM 34:0;O2	n.d.	34	30.94	0.28	705.5905 (-0.4)	184.0733	749.5812 (-3.8)
SM 34:1;O2	n.d.	32	32.17	0.28	703.5749 (3.4)	184.0733	747.5659 (-3.7)
SM 34:2;O2	n.d.	30	28.86	0.28	701.5592 (1.3)	184.0733	745.5502 (-3.4)
SM 35:1;O2	n.d.	33	22.37	0.28	717.5905 (2.1)	184.0733	761.5815 (-0.7)
SM 36:1;O2	n.d.	34	38.32	0.28	731.6062 (1.6)	184.0733	775.5972 (3.5)
SM 36:2;O2	n.d.	32	31.08	0.28	729.5905 (2.6)	184.0733	773.5815 (0.7)
SM 38:1;O2	n.d.	36	48.20	0.28	759.6375 (-1.8)	184.0733	803.6285 (-2.9)
SM 38:2;O2	n.d.	34	40.44	0.28	757.6218 (0.3)	184.0733	801.6128 (-4.8)
SM 39:1;O2	n.d.	37	52.72	0.28	773.6531 (0.4)	184.0733	817.6441 (2.3)
SM 40:1;O2	n.d.	38	56.15	0.28	787.6688 (3.0)	184.0733	831.6598 (3.2)
SM 40:2;O2	n.d.	36	50.08	0.28	785.6531 (3.1)	184.0733	829.6441 (0.4)
SM 41:1;O2	n.d.	39	59.75	0.28	801.6844 (3.4)	184.0733	845.6754 (-4.3)
SM 41:2;O2	n.d.	37	54.20	0.28	799.6688 (1.5)	184.0733	843.6598 (-3.9)
SM 42:1;O2	n.d.	40	62.76	0.28	815.7001 (3.2)	184.0733	859.6911 (4.2)
SM 42:2;O2	n.d.	38	56.15	0.28	813.6844 (2.0)	184.0733	857.6754 (4.4)
SM 42:3;O2	n.d.	36	49.91	0.28	811.6688 (0.1)	184.0733	855.6598 (2.2)
SM 43:2;O2	n.d.	39	58.24	0.28	827.7001 (1.2)	184.0733	871.6911 (4.2)

Phophatidylcholines

Lipid species level	Lipid acyl/alkyl level	ECN	D^1 t_R [min]	D^2 t_R [min]	[M+H]$^+$ (ppm)	*m/z* 184	[M+formate]$^-$ (ppm)	*sn-1*/*sn-2* RCOO$^-$
PC 30:0	PC 14:0/16:0	30	28.95	0.22	706.5381 (5.7)	184.0733	750.5291 (2.7)	227.2017 / 255.233
PC 32:0	PC 16:0/16:0	32	39.78	0.22	734.5694 (2.0)	184.0733	778.5604 (3.9)	255.2330
PC 32:1	PC 16:0/16:1	30	32.17	0.22	732.5538 (3.0)	184.0733	776.5447 (-0.4)	255.2330 / 253.2173
PC 32:2	PC 14:0/18:2	28	25.83	0.22	730.5381 (0.8)	184.0733	774.5291 (-2.2)	227.2017 / 279.2330

PC 33:1	PC 15:0/18:1	31	36.63	0.22	746.5694 (0.8)	184.0733	790.5604 (2.8)	241.2173 / 281.2486
PC 33:2	PC 15:0/18:2	29	28.29	0.22	744.5538 (3.9)	184.0733	788.5447 (-1.6)	241.2173 / 279.2330
PC 34:0	PC 18:0/16:0	34	49.09	0.22	762.6007 (2.4)	184.0733	806.5917 (4.8)	283.2643 / 255.2330
PC 34:1	PC 16:0/18:1	32	41.16	0.22	760.5851 (3.7)	184.0733	804.5760 (-4.4)	255.2330 / 281.2486
PC 34:2	PC 16:0/18:2	30	34.44	0.22	758.5694 (3.7)	184.0733	802.5604 (-4.7)	255.2330 / 279.2330
PC 34:3	PC 16:0/18:3	28	28.77	0.22	756.5538 (3.3)	184.0733	800.5447 (4.5)	255.2330 / 277.2173
PC 34:4	PC 14:0/20:4	26	24.97	0.22	754.5381 (2.1)	184.0733	798.5291 (2.5)	227.2017 / 303.2330
PC 35:1	PC 17:0/18:1	33	46.01	0.22	774.6007 (1.9)	184.0733	818.5917 (2.3)	269.2486 / 281.2486
PC 35:2	PC 17:0/18:2	31	39.10	0.22	772.5851 (3.1)	184.0733	816.5760 (2.7)	269.2486 / 279.2330
PC 35:3	PC 15:0/20:3	29	31.32	0.22	770.5694 (1.9)	184.0733	814.5604 (4.3)	241.2173 / 305.2486
PC 35:4	PC 15:0/20:4	37	29.07	0.22	768.5538 (0.7)	184.0733	812.5447 (0.1)	241.2173 / 303.2330
PC 36:1	PC 18:0/18:1	34	50.41	0.22	788.6164 (3.6)	184.0733	832.6073 (0.4)	283.2643 / 281.2486
PC 36:2	PC 18:0/18:2	32	43.90	0.22	786.6007 (4.2)	184.0733	830.5917 (-0.4)	283.2643 / 279.2330
PC 36:3	PC 16:0/20:3	30	37.19	0.22	784.5851 (3.4)	184.0733	828.5760 (-0.8)	255.2330 / 305.2486
	PC 18:1/18:2	30	35.70	0.22	784.5851 (2.3)	184.0733	828.5760 (-0.8)	281.2486 / 279.2330
PC 36:4	PC 18:2/18:2	28	33.48	0.22	782.5694 (3.8)	184.0733	826.5604 (-2.1)	279.2330
	PC 16:0/20:4	28	33.48	0.22	782.5694 (3.8)	184.0733	826.5604 (-2.1)	255.2330 / 303.2330
PC 36:5	PC 16:0/20:5	26	28.06	0.22	780.5538 (2.8)	184.0733	824.5447 (-2.1)	255.2330 / 301.2173
PC 37:3	PC 17:0/20:3	31	41.16	0.22	798.6007 (3.1)	184.0733	842.5917 (1.3)	269.2486 / 305.2486
PC 37:4	PC 17:0/20:4	29	34.44	0.22	796.5851 (1.4)	184.0733	840.5760 (3.9)	269.2486 / 303.2330
PC 38:2	PC 18:1/20:1	34	51.70	0.22	814.6320 (3.2)	184.0733	858.6230 (-3.6)	281.2486 / 309.2799
	PC 18:0_20:2	34	51.70	0.22	814.6320 (3.2)	184.0733	858.6230 (-3.6)	283.2643 / 307.2643
PC 38:3	PC 18:0/20:3	32	46.49	0.22	812.6164 (2.3)	184.0733	856.6073 (-4.4)	283.2643 / 305.2486
PC 38:4	PC 18:1/20:3	30	42.76	0.22	810.6007 (3.6)	184.0733	854.5917 (-4.6)	281.2486 / 305.2486
	PC 18:0/20:4	30	42.76	0.22	810.6007 (3.6)	184.0733	854.5917 (-4.6)	283.2643 / 303.2330
PC 38:5	PC 16:0/22:5	28	34.44	0.22	808.5851 (1.5)	184.0733	852.5760 (3.2)	255.2330 / 329.2486
	PC 18:1/20:4	28	34.44	0.22	808.5851 (1.5)	184.0733	852.5760 (3.2)	281.2486 / 303.2330
	PC 18:0/20:5	28	37.19	0.22	808.5851 (3.0)	184.0733	852.5760 (3.2)	283.2643 / 301.2173
PC 38:6	PC 16:0/22:6	26	31.52	0.22	806.5694 (3.3)	184.0733	850.5604 (2.5)	255.2330 / 327.2330

	PC 18:2/20:4	26	31.52	0.22	806.5694 (3.3)	184.0733	850.5604 (2.5)	279.2330 / 303.2330
PC 40:4	PC 18:0/22:4	32	49.27	0.22	838.6320 (1.8)	184.0733	882.6230 (2.0)	283.2643 / 331.2643
PC 40:5	PC 18:0_22:5	30	43.53	0.22	836.6164 (2.3)	184.0733	880.6073 (-3.3)	283.2643 / 329.2486
	PC 20:1_20:4	30	43.53	0.22	836.6164 (2.3)	184.0733	880.6073 (-3.3)	309.2799 / 303.2330
PC 40:6	PC 18:0/22:6	28	40.83	0.22	834.6007 (3.5)	184.0733	878.5917 (-2.8)	283.2643 / 327.2330
PC 40:7	PC 18:1/22:6	27	32.77	0.22	832.5851 (1.9)	184.0733	876.5760 (-3.9)	281.2486 / 327.2330
PC 40:8	n.d.	26	26.69	0.22	830.5695 (2.3)	184.0733	874.5604 (-2.9)	n.d.

Phophatidylcholines - plasmalogens

**Lipid species level**	**Lipid acyl/alkyl level**	**ECN**	**D^1 t_R [min]**	**D^2 t_R [min]**	**$[M+H]^+$ (ppm)**	***m/z* 184**	**$[M+formate]^-$ (ppm)**	***sn-2* $RCOO^-$**
PC P-32:0	PC P-16:0/16:0	32	44.03	0.22	718.5745 (-1.3)	184.0733	762.5655 (4.7)	255.2330
PC P-34:1	PC P-16:0/18:1	32	45.35	0.22	744.5902 (0.4)	184.0733	788.5811 (-2.3)	281.2486
PC P-34:2	PC P-16:0/18:2	30	38.32	0.22	742.5745 (0.8)	184.0733	786.5655 (-1.0)	279.2330
PC P-36:2	PC P-18:0/18:2	32	46.55	0.22	770.6058 (-0.4)	184.0733	814.5968 (4.2)	279.2330
PC P-36:3	PC P-18:1/18:2	30	38.68	0.22	768.5902 (2.7)	184.0733	812.5811 (2.3)	279.2330
PC P-38:4	PC P-18:0/20:4	30	39.60	0.22	794.6058 (2.6)	184.0733	838.5968 (-3.5)	303.2330

Phosphatidylethanolamines

**Lipid species level**	**Lipid acyl/alkyl level**	**ECN**	**D^1 t_R [min]**	**D^2 t_R [min]**	**$[M+H]^+$ (ppm)**	**$[M-H]^-$ (ppm)**	***sn-1/sn-2* $RCOO^-$**
PE 34:1	PE 16:0/18:1	32	41.61	0.14	718.5381 (2.9)	716.5236 (-2.8)	255.2330 / 281.2486
PE 34:2	PE 16:0/18:2	30	34.81	0.14	716.5225 (3.8)	714.5079 (-2.5)	255.2330 / 279.2330
PE 36:1	PE 18:0/18:1	34	49.65	0.14	746.5694 (2.0)	744.5549 (1.9)	283.2643 / 281.2486
PE 36:2	PE 18:0/18:2	32	44.08	0.14	744.5538 (2.6)	742.5392 (3.5)	283.2643 / 279.2330
PE 36:3	PE 18:1/18:2	30	36.04	0.14	742.5381 (2.4)	740.5236 (2.8)	281.2486 / 279.2330
PE 36:4	PE 16:0/20:4	28	33.57	0.14	740.5225 (4.9)	738.5079 (-1.8)	255.2330 / 303.2330
PE 38:3	PE 18:0/20:3	32	45.93	0.14	770.5694 (2.9)	768.5549 (-3.3)	283.2643 / 305.2486
PE 38:4	PE 18:0/20:4	30	42.84	0.14	768.5538 (4.6)	766.5392 (-4.2)	283.2643 / 303.2330
PE 38:5	PE 18:1/20:4	28	37.28	0.14	766.5381 (2.5)	764.5236 (-1.0)	281.2486 / 303.2330
PE 38:6	PE 16:0/22:6	26	31.72	0.14	764.5225 (3.5)	762.5079 (-3.5)	255.2330 / 327.2330
PE 40:4	PE 18:0/22:4	32	48.42	0.14	796.5851 (4.0)	794.5705 (-2.8)	283.2643 / 331.2643

PE 40:5	PE 18:0/22:5	30	43.46	0.14	794.5694 (3.1)	792.5549 (-4.5)	283.2643 / 329.2486
PE 40:6	PE 18:0/22:6	28	40.99	0.14	792.5538 (3.2)	790.5392 (-4.8)	283.2643 / 327.2330
PE 40:7	PE 18:1/22:6	26	38.04	0.14	790.5381 (4.7)	788.5236 (-0.1)	281.2486 / 327.2330

Phosphatidylethanolamines - plasmalogens

Lipid species level	Lipid acyl/alkyl level	ECN	D^1 t_R [min]	D^2 t_R [min]	[M+H]$^+$ (ppm)	[$R_2COO(C_3H_5)OH$]$^+$	[M-H]$^-$ (ppm)	*sn-2* RCOO$^-$
PE P-34:1	PE P-16:0/18:1	32	45.31	0.14	702.5432 (4.8)	339.2894	700.5287 (-4.6)	281.2486
PE P-34:2	PE P-16:0/18:2	30	39.13	0.14	700.5276 (4.0)	337.2737	698.513 (-0.7)	279.2330
PE P-36:1	PE P-18:0/18:1	34	56.57	0.14	730.5745 (-1.2)	339.2894	728.5600 (-0.7)	281.2486
PE P-36:2	PE P-18:0/18:2	32	50.92	0.14	728.5589 (1.9)	339.2894	726.5443 (-1.5)	279.2330
PE P-36:3	PE P-18:1/18:2	30	42.70	0.14	726.5432 (2.9)	337.2737	724.5287 (-1.2)	279.2330
PE P-36:4	PE P-16:0/20:4	28	40.21	0.14	724.5276 (-0.7)	361.2737	722.513 (2.2)	303.2330
PE P-36:5	PE P-16:0/20:5	26	34.39	0.14	722.5119 (2.1)	359.2581	720.4974 (-4.3)	301.2173
PE P-38:2	PE P-20:0/18:2	34	54.6	0.14	756.5902 (4.5)	337.2737	754.5756 (3.8)	279.2330
PE P-38:3	PE P-18:0/20:3	32	49.65	0.14	754.5745 (-2.4)	363.2894	752.5600 (-3.6)	305.2486
PE P-38:4	PE P-18:0/20:4	30	49.71	0.14	752.5589 (4.7)	361.2737	750.5443 (-2.1)	303.2330
PE P-38:5	PE P-18:1/20:4	28	41.38	0.14	750.5432 (-2.3)	361.2737	748.5287 (-2.4)	303.2330
PE P-38:6	PE P-16:0/22:6	26	38.21	0.14	748.5276 (2.1)	385.2737	746.513 (-0.1)	327.2330
PE P-40:4	PE P-20:0/20:4	32	51.51	0.14	780.5902 (1.8)	361.2737	778.5756 (4.8)	303.2330
PE P-40:5	PE P-20:1/20:4	30	50.51	0.14	778.5745 (-2.2)	361.2737	776.5600 (2.7)	303.2330
PE P-40:6	PE P-18:0/22:6	28	44.7	0.14	776.5589 (-1.0)	385.2737	774.5443 (1.9)	327.2330

Phosphatidylinositols

Lipid species level	Lipid acyl/alkyl level	ECN	D^1 t_R [min]	D^2 t_R [min]	[M-H]$^-$ (ppm)	*sn-1/sn-2* RCOO$^-$
PI 32:1	PI 16:0_16:1	30	25.43	0.16	807.5029 (-1.2)	255.2330 / 253.2173
PI 34:1	PI 16:0_18:1	32	33.47	0.16	835.5342 (-2.9)	255.2330 / 281.2486
PI 34:2	PI 16:0_18:2	30	27.27	0.16	833.5186 (-4.1)	255.2330 / 279.2330
PI 36:1	PI 18:0_18:1	34	42.11	0.16	863.5655 (1.8)	283.2643 / 281.2486
PI 36:2	PI 18:0_18:2	32	35.8	0.16	861.5499 (0.3)	283.2643 / 279.2330
PI 36:3	PI 18:1_18:2	30	29.69	0.16	859.5342 (-2.3)	281.2486 / 279.2330

PI 36:4	PI 16:0_20:4	28	26.64	0.16	857.5186 (0.9)	255.2330 / 303.2330
PI 38:3	PI 18:0_20:3	32	41.22	0.16	887.5655 (1.9)	283.2643 / 305.2486
PI 38:4	PI 18:0_20:4	30	35.05	0.16	885.5499 (3.7)	283.2643 / 303.2330
PI 38:5	PI 18:0_22:5	28	30.72	0.16	883.5342 (4.0)	281.2486 / 303.2330
PI 38:6	PI 16:0_22:6	26	25.09	0.16	881.5186 (-2.3)	255.2330 / 327.2330
PI 40:4	PI 18:0_22:4	32	41.24	0.16	913.5812 (-0.8)	283.2643 / 331.2643
PI 40:5	PI 18:0_22:5	30	35.63	0.16	911.5655 (-2.7)	283.2643 / 329.2486
PI 40:6	PI 18:0_22:6	28	33.21	0.16	909.5499 (3.3)	283.2643 / 327.2330

Diacylglycerols

Lipid species level	Lipid acyl/alkyl level	ECN	D^1 t_R [min]	D^2 t_R [min]	$[M+NH_4]^+$ (ppm)	$[M+H-H_2O]^+$	Neutral loss of *sn-1/sn-2/sn-3*
DG 30:0	DG 14:0_16:0	30	49.51	0.03	558.5092 (4.3)	523.4721	313.2737 / 285.2424
DG 32:0	DG 16:0/16:0	32	56.68	0.03	586.5405 (4.7)	551.5034	313.2737
DG 32:1	DG 16:0_16:1	30	50.83	0.03	584.5248 (4.4)	549.4877	311.2581 / 313.2737
	DG 18:1_14:0	30	50.83	0.03	584.5248 (4.4)	549.4877	339.2894 / 285.2424
DG 32:2	DG 18:1_14:0	28	44.29	0.03	582.5092 (4.9)	547.4721	337.2737 / 285.2424
DG 34:0	DG 16:0_18:0	34	66.3	0.03	614.5718 (4.2)	579.5347	341.3050 / 313.2737
DG 34:1	DG 16:0_18:1	32	61.18	0.03	612.5561 (1.6)	577.5190	339.2894 / 313.2737
DG 34:2	DG 16:1_18:1	30	56.51	0.03	610.5405 (3.9)	575.5034	339.2894 / 311.2581
	DG 16:0_18:2	30	56.51	0.03	610.5405 (3.9)	575.5034	337.2737 / 313.2737
DG 34:3	DG 18:2_16:1	28	46.26	0.03	608.5248 (4.6)	573.4877	337.2737 / 311.2581
DG 35:1	DG 18:1_17:0	33	59.94	0.03	626.5718 (-2.9)	591.5347	339.2894 / 327.2894
DG 36:0	DG 18:0/18:0	36	71.63	0.03	642.6031 (-1.4)	607.5660	341.3050
DG 36:1	DG 18:0_18:1	34	66.95	0.03	640.5874 (1.4)	605.5503	339.2894 / 341.3050
DG 36:2	DG 18:1/18:1	32	61.92	0.03	638.5718 (3.9)	603.5347	339.2894
DG 36:3	DG 18:1_18:2	30	57.02	0.03	636.5561 (4.4)	601.5190	339.2894 / 337.2737
DG 36:4	DG 18:1_18:3	28	51.92	0.03	634.5405 (4.3)	599.5034	335.2588 / 339.2894
	DG 18:2/18:2	26	51.92	0.03	634.5405 (4.3)	599.5034	337.2737
DG 38:4	n.d.	30	54.72	0.03	662.5718 (4.5)	627.5347	n.d.

DG 38:5	DG 18:1_20:4	28	51.47	0.03	660.5561 (4.8)	625.519	361.2737 / 339.2894
DG 38:6	n.d.	26	46.25	0.03	658.5405 (5.0)	623.5034	n.d.

Cholesterol and sterolesters

Lipid species level	Lipid acyl/alkyl level	ECN	D^1 t_R [min]	D^2 t_R [min]	$[M+NH_4]^+$ (ppm)	*m/z* 369
Chol	Chol	-	31.24	0.03	-	369.3516
SE 27:1/16:0	SE 27:1/16:0	16	89.69	0.03	642.6183 (-1.7)	369.3516
SE 27:1/16:1	SE 27:1/16:1	14	86.56	0.03	640.6027 (0.5)	369.3516
SE 27:1/18:0	SE 27:1/18:0	18	91.99	0.03	670.6496 (0.4)	369.3516
SE 27:1/18:1	SE 27:1/18:1	16	89.81	0.03	668.6340 (2.5)	369.3516
SE 27:1/18:2	SE 27:1/18:2	14	87.07	0.03	666.6183 (4.3)	369.3516
SE 27:1/18:3	SE 27:1/18:3	12	84.62	0.03	664.6027 (0.2)	369.3516
SE 27:1/20:1	SE 27:1/20:1	18	94.26	0.03	696.6653 (3.6)	369.3516
SE 27:1/20:2	SE 27:1/20:2	16	90.19	0.03	694.6496 (0.6)	369.3516
SE 27:1/20:3	SE 27:1/20:3	14	87.62	0.03	692.6340 (4.6)	369.3516
SE 27:1/20:4	SE 27:1/20:4	12	85.60	0.03	690.6183 (2.8)	369.3516
SE 27:1/20:5	SE 27:1/20:5	10	83.05	0.03	688.6027 (1.6)	369.3516
SE 27:1/22:1	SE 27:1/22:2	18	96.33	0.03	724.6966 (2.8)	369.3516
SE 27:1/22:4	SE 27:1/22:4	14	88.22	0.03	718.6496 (1.3)	369.3516
SE 27:1/22:5	SE 27:1/22:5	12	85.99	0.03	716.6340 (2.4)	369.3516
SE 27:1/22:6	SE 27:1/22:6	10	84.22	0.03	714.6183 (1.1)	369.3516

Triacylglycerols

Lipid species level	Lipid acyl/alkyl level	ECN	D^1 t_R [min]	D^2 t_R [min]	$[M+NH_4]$+ (ppm)	$[M+Na]^+$	Neutral loss of *sn-1/sn-2/sn-3*
TG 42:0	TG 16:0_14:0_12:0	42	81.06	0.03	740.6763 (1.9)	745.6317	523.4721 / 495.4408 / 467.4095
TG 42:1	TG 12:0_12:0_18:1	40	77.99	0.03	738.6606 (3.2)	743.6160	521.4564 / 521.4564 / 439.3782
TG 42:2	TG 12:0_12:0_18:2	38	74.88	0.03	736.6450 (4.5)	741.6004	519.4408 / 519.4408 / 439.3782
TG 44:0	TG 16:0_14:0_14:0	44	84.22	0.03	768.7076 (2.9)	773.6630	495.4408 / 523.4721 / 523.4721
TG 44:1	TG 14:0_18:1_12:0	42	81.37	0.03	766.6919 (3.9)	771.6473	521.4564 / 467.4095 / 549.4877
	TG 12:0_16:0_16:1	42	81.37	0.03	766.6919 (3.9)	771.6473	549.4877 / 495.4408 / 493.4251

	TG 16:1_14:0_14:0	42	81.37	0.03	766.6919 (3.9)	771.6473	495.4408 / 521.4564 / 521.4564
TG 44:2	TG 14:0_18:2_12:0	40	78.76	0.03	764.6763 (3.3)	769.6317	519.4408 / 467.4095 / 547.4721
TG 46:0	TG 16:0_16:0_14:0	46	86.92	0.03	796.7389 (4.4)	801.6943	523.4721 / 523.4721 / 551.5034
TG 46:1	TG 16:0_18:1_12:0	44	84.48	0.03	794.7232 (3.5)	799.6786	521.4564 / 495.4408 / 577.5190
	TG 16:0_16:1_14:0	44	84.48	0.03	794.7232 (3.5)	799.6786	521.4564 / 523.4721 / 549.4877
TG 46:2	TG 16:0_18:2_12:0	42	82.25	0.03	792.7076 (4.5)	797.6630	519.4408 / 495.4408 / 575.5034
TG 46:3	TG 12:0_16:1_18:2	40	79.56	0.03	790.6919 (3.9)	795.6473	573.4877 / 519.4408 / 493.4251
	TG 14:0_14:1_18:2	40	79.56	0.03	790.6919 (3.9)	795.6473	547.4721 / 545.4564 / 493.4251
TG 48:0	TG 16:0_16:0_16:0	48	89.49	0.03	824.7702 (3.5)	829.7256	551.5034 / 551.5034 / 551.5034
	TG 18:0_16:0_14:0	48	89.49	0.03	824.7702 (3.5)	829.7256	523.4721 / 551.5034 / 579.5347
TG 48:1	TG 16:0_16:1_16:0	46	87.10	0.03	822.7545 (4.0)	827.7099	549.4877 / 551.5034 / 549.4877
	TG 16:0_18:1_14:0	46	87.10	0.03	822.7545 (4.0)	827.7099	549.4877 / 523.4721 / 577.5190
	TG 18:0_18:1_12:0	46	87.10	0.03	822.7545 (4.0)	827.7099	521.4564 / 523.4721 / 605.5503
TG 48:2	TG 18:1_18:1_12:0	44	84.73	0.03	820.7389 (2.7)	825.6943	521.4564 / 521.4564 / 603.5347
	TG 16:0_18:2_14:0	44	84.73	0.03	820.7389 (2.7)	825.6943	547.4721 / 523.4721 / 575.5034
	TG 18:1_16:1_14:0	44	84.73	0.03	820.7389 (2.7)	825.6943	521.4564 / 549.4877 / 575.5034
TG 48:3	TG 18:1_18:2_12:0	42	82.86	0.03	818.7232 (3.5)	823.6786	519.4408 / 521.4564 / 601.5190
TG 49:0	TG 17:0_16:0_16:0	49	90.64	0.03	838.7858 (4.9)	843.7412	551.5034 / 565.5190 / 565.5190
TG 49:1	TG 15:0_16:0_18:1	47	88.39	0.03	836.7702 (4.8)	841.7256	577.5190 / 563.5034 / 537.4877
TG 49:2	TG 15:0_16:0_18:2	45	86.30	0.03	834.7545 (4.4)	839.7099	575.5034 / 561.4877 / 537.4877
TG 49:3	TG 18:2_16:1_15:0	43	84.22	0.03	832.7389 (4.3)	837.6943	535.4721 / 561.4877 / 573.4877
TG 50:0	TG 18:0_16:0_16:0	50	91.94	0.03	852.8015 (4.5)	857.7569	551.5034 / 579.5347 / 579.5347
TG 50:1	TG 16:0_18:1_16:0	48	89.61	0.03	850.7858 (4.1)	855.7412	577.5190 / 551.5034 / 577.5190
	TG 18:0_18:1_14:0	48	89.61	0.03	850.7858 (4.1)	855.7412	549.4877 / 551.5034 / 605.5503
TG 50:2	TG 18:1_18:1_14:0	46	87.34	0.03	848.7702 (4.2)	853.7256	549.4877 / 549.4877 / 603.5347
	TG 16:0_16:0_18:2	46	87.34	0.03	848.7702 (4.2)	853.7256	575.5034 / 575.5034 / 551.5034
	TG 16:0_18:1_16:1	46	87.34	0.03	848.7702 (4.2)	853.7256	575.5034 / 549.4877 / 577.5190
TG 50:3	TG 18:1_18:2_14:0	44	85.01	0.03	846.7545 (3.7)	851.7099	547.4721 / 549.4877 / 601.5190
	TG 16:1_16:0_18:2	44	85.01	0.03	846.7545 (3.7)	851.7099	575.5034 / 573.4877 / 549.4877

	TG 16:1_16:1_18:1	44	85.01	0.03	846.7545 (3.7)	851.7099	575.5034 / 575.5034 / 547.4721
TG 50:4	TG 18:2_16:1_16:1	42	82.86	0.03	844.7389 (2.8)	849.6943	547.4721 / 573.4877 / 573.4877
	TG 18:3_16:1_16:0	42	82.86	0.03	844.7389 (2.8)	849.6943	573.4877 / 571.4721 / 549.4877
TG 51:1	TG 17:0_18:1_16:0	49	90.85	0.03	864.8015 (4.5)	869.7569	565.5190 / 577.5190 / 591.5347
TG 51:2	TG 17:1_18:1_16:0	47	88.59	0.03	862.7858 (4.2)	867.7412	577.5195 / 563.5034 / 589.5190
TG 51:3	TG 18:2_15:0_18:1	45	86.47	0.03	860.7702 (4.3)	865.7256	601.5190 / 563.5034 / 561.4877
	TG 17:1_18:2_16:0	45	86.47	0.03	860.7702 (4.3)	865.7256	575.5034 / 563.5034 / 587.5034
TG 51:4	TG 18:2_18:2_15:0	43	84.22	0.03	858.7545 (4.1)	863.7099	561.4877 / 561.4877 / 599.5034
TG 52:0	TG 18:0_18:0_16:0	52	94.12	0.03	880.8328 (4.9)	885.7882	579.5347 / 579.5347 / 607.5660
TG 52:1	TG 18:0_18:1_16:0	50	91.99	0.03	878.8171 (4.4)	883.7725	577.5190 / 579.5347 / 605.5503
TG 52:2	TG 18:1_18:1_16:0	48	89.78	0.03	876.8015 (3.4)	881.7569	577.5190 / 577.5190 / 603.5347
TG 52:3	TG 18:1_18:1_16:1	46	87.70	0.03	874.7858 (4.8)	879.7412	575.5034 / 575.5034 / 603.5347
	TG 18:1_18:2_16:0	46	87.70	0.03	874.7858 (4.8)	879.7412	575.5034 / 577.5190 / 601.5190
TG 52:4	TG 18:1_18:2_16:1	44	85.53	0.03	872.7702 (2.7)	877.7256	573.4877 / 575.5034 / 601.5190
	TG 18:2_18:2_16:0	44	85.53	0.03	872.7702 (2.7)	877.7256	575.5034 / 575.5034 / 599.5034
TG 52:5	TG 18:2_18:2_16:1	42	83.44	0.03	870.7545 (3.0)	875.7099	573.4877 / 573.4877 / 599.5034
	TG 16:0_16:1_20:4	42	83.44	0.03	870.7545 (3.0)	875.7099	599.5034 / 597.4877 / 549.4877
	TG 18:3_18:2_16:0	42	83.44	0.03	870.7545 (3.0)	875.7099	575.5034 / 573.4877 / 597.4877
TG 53:1	TG 18:0_18:1_17:0	51	93.16	0.03	892.8328 (3.5)	897.7882	519.4408 / 521.4564 / 601.5190
TG 53:2	TG 18:1_18:1_17:0	49	90.99	0.03	892.8328 (3.5)	895.7725	591.5347 / 591.5347 / 603.5347
TG 53:3	TG 18:1_18:2_17:0	47	88.91	0.03	888.8015 (4.1)	893.7569	589.5190 / 591.5347 / 601.5190
TG 54:1	TG 20:0_18:1_16:0	52	94.26	0.03	906.8484 (3.0)	911.8038	577.5190 / 607.5660 / 633.5816
	TG 18:1_18:0_18:0	52	94.26	0.03	906.8484 (3.0)	911.8038	607.5660 / 605.5503 / 605.5503
	TG 18:0_20:1_16:0	52	94.26	0.03	906.8484 (3.0)	911.8038	605.5503 / 579.5347 / 633.5816
TG 54:2	TG 20:1_18:1_16:0	50	92.10	0.03	904.8328 (4.1)	909.7882	577.5190 / 605.5503 / 631.5660
	TG 18:0_18:1_18:1	50	92.10	0.03	904.8328 (4.1)	909.7882	603.5347 / 605.5503 / 605.5503
TG 54:3	TG 18:1_18:1_18:1	48	90.03	0.03	902.8171 (4.5)	907.7725	603.5347 / 603.5347 / 603.5347
	TG 18:0_18:2_18:1	48	90.03	0.03	902.8171 (4.5)	907.7725	601.5190 / 605.5503 / 603.5347
TG 54:4	TG 18:1_18:2_18:1	46	87.88	0.03	900.8015 (4.2)	905.7569	601.5190 / 603.5347 / 601.5190

	TG 16:0_18:1_20:3	46	87.88	0.03	900.8015 (4.2)	905.7569	627.5347 / 601.5190 / 577.5190
	TG 18:0_18:2_18:2	46	87.88	0.03	900.8015 (4.2)	905.7569	599.5034 / 603.5347 / 603.5347
TG 54:5	TG 16:0_18:2_20:3	44	85.80	0.03	898.7858 (2.8)	903.7412	625.5190 / 601.5190 / 575.5034
	TG 18:1_18:2_18:2	44	85.80	0.03	898.7858 (2.8)	903.7412	599.5034 / 601.5190 / 601.5190
	TG 18:1_18:1_18:3	44	85.80	0.03	898.7858 (2.8)	903.7412	599.5034 / 599.5034 / 603.5347
TG 54:6	TG 18:1_18:2_18:3	42	83.63	0.03	896.7702 (3.3)	901.7256	597.4877 / 599.5034 / 601.5190
	TG 20:4_18:1_16:1	42	83.63	0.03	896.7702 (3.3)	901.7256	575.5034 / 597.4877 / 625.5190
	TG 20:4_18:2_16:0	42	83.63	0.03	896.7702 (3.3)	901.7256	575.5034 / 599.5034 / 623.5034
TG 56:1	TG 24:1_16:0_16:0	54	96.46	0.03	934.8797 (3.6)	939.8351	551.5034 / 661.6129 / 661.6129
TG 56:2	TG 20:1_18:1_18:0	52	94.34	0.03	932.8641 (4.3)	937.8195	605.5503 / 633.5816 / 631.5660
	TG 22:0_18:2_16:0	52	94.34	0.03	932.8641 (4.3)	937.8195	575.5034 / 635.5973 / 659.5973
TG 56:3	TG 20:1_18:1_18:1	50	92.23	0.03	930.8484 (4.7)	935.8038	603.5347 / 631.5660 / 631.5660
TG 56:4	TG 16:0_18:0_22:4	48	90.33	0.03	928.8328 (3.2)	933.7882	655.5660 / 627.5347 / 579.5347
TG 56:5	TG 22:4_16:0_18:1	46	88.91	0.03	926.8171 (2.6)	931.7725	577.5190 / 653.5503 / 627.5347
	TG 20:4_18:1_18:0	46	88.91	0.03	926.8171 (2.6)	931.7725	605.5503 / 625.5190 / 627.5347
TG 56:6	TG 18:1_18:1_20:4	44	87.10	0.03	924.8015 (2.7)	929.7569	625.5190 / 625.5190 / 603.5347
	TG 18:0_18:2_20:4	44	87.10	0.03	924.8015 (2.7)	929.7569	627.5347 / 623.5034 / 603.5347
TG 56:7	TG 22:5_18:2_16:0	42	86.11	0.03	922.7858 (3.8)	927.7412	649.5190 / 625.5190 / 649.5190
TG 56:8	TG 22:6_18:2_16:0	40	83.88	0.03	920.7702 (4.2)	925.7256	575.5034 / 623.5034 / 647.5034
TG 58:3	TG 22:0_18:2_18:1	52	94.56	0.03	958.8797 (1.8)	963.8351	601.5190 / 661.6129 / 659.5973

n.d. … not detected

Table S4. Characteristic ions of individual lipid classes used for the identification of lipid species observed in positive and negative ESI mode.

**Lipid Class**	**Positive-ESI mode**	**Negative-ESI mode**
Cer	$[M+H-H_2O]^+$; sphingoid base	$[M+formate]^-$
HexCer	$[M+H]^+$; sphingoid base	$[M+formate]^-$
Hex2Cer	$[M+H]^+$	$[M+formate]^-$
GM3	n.d.	$[M-H]^-$
SM	$[M+H]^+$; *m/z* 184	$[M+formate]^-$
PC	$[M+H]^+$; *m/z* 184	$[M+formate]^-$; *sn-1/sn-2* $RCOO^-$
PC P	$[M+H]^+$; *m/z* 184	$[M+formate]^-$; *sn-2* $RCOO^-$
LPC	$[M+H]^+$; *m/z* 184	$[M+formate]^-$
PE	$[M+H]^+$; neutral loss of *m/z* 141	$[M-H]^-$; *sn-1/sn-2* $RCOO^-$
PE P	$[M+H]^+$; $[R_2COO(C_3H_5)OH]^+$	$[M-H]^-$; *sn-2* $RCOO^-$
PI	n.d.	$[M-H]^-$; *sn-1/sn-2* $RCOO^-$
Chol	$[M+H-H_2O]^+$	n.d.
CE	$[M+NH_4]^+$; *m/z* 369	n.d.
DG	$[M+NH_4]^+$; $[M+H-H_2O]^+$; Neutral loss of *sn-1/sn-2/sn-3* fatty acyl	n.d.
TG	$[M+NH_4]^+$, $[M+Na]^+$, neutral loss of *sn-1/sn-2/sn-3* fatty acyl	n.d.
FA	n.d.	$[M-H]^-$

n.d. … not detected
